# Risk factors of amyotrophic lateral sclerosis: a global meta-summary

**DOI:** 10.3389/fnins.2023.1177431

**Published:** 2023-04-24

**Authors:** Qing-Qing Duan, Zheng Jiang, Wei-Ming Su, Xiao-Jing Gu, Han Wang, Yang-Fan Cheng, Bei Cao, Xia Gao, Yi Wang, Yong-Ping Chen

**Affiliations:** ^1^Department of Neurology, West China Hospital, Sichuan University, Chengdu, Sichuan, China; ^2^Lab of Neurodegenerative Disorders, Clinical Institute of Inflammation and Immunology (CIII), Frontiers Science Center for Disease-Related Molecular Network, West China Hospital, Sichuan University, Chengdu, Sichuan, China; ^3^Centre for Rare Diseases, West China Hospital, Sichuan University, Chengdu, Sichuan, China; ^4^Mental Health Center, West China Hospital, Sichuan University, Chengdu, Sichuan, China; ^5^Department of Pathophysiology, West China College of Basic Medical Sciences & Forensic Medicine, Sichuan University, Chengdu, China; ^6^Department of Geriatrics, Dazhou Central Hospital, Dazhou, Sichuan, China

**Keywords:** amyotrophic lateral sclerosis (ALS), risk, etiology, genes, onset, odds ratio

## Abstract

**Background:**

The etiology of amyotrophic lateral sclerosis (ALS) remains largely unknown. This study aimed to summarize the relationship between ALS and its genetic and non-genetic risk factors.

**Method:**

A search of relevant literature from PubMed, Embase, and Cochrane Database from inception to December 2022 was performed. Random-effects or fixed-effects models were performed by Stata MP 15.0 to pool multivariate or adjusted ratios (OR). PROSPERO registration number: CRD42022301549.

**Results:**

230 eligible studies were included, of which 67 involved 22 non-genetic factors, and 163 involved genetic factors. Four aspects of non-genetic factors, including lifestyle, environmental and occupational exposures, pre-existing diseases/comorbidity and medical exposures, and others, were analyzed. Exposure to heavy metals (OR = 1.79), pesticides (OR = 1.46), solvents (OR = 1.37), previous head trauma (OR = 1.37), military service (OR = 1.29), stroke (OR = 1.26), magnetic field (OR = 1.22) and hypertension (OR = 1.04) are significant risk factors, but use of antidiabetics (OR = 0.52), high BMI (OR = 0.60 for obese and overweight vs. normal and underweight), living in urban (OR = 0.70), diabetes mellitus (OR = 0.83), and kidney disease (OR = 0.84) decrease the risk for ALS. In addition, eight common ALS-related genes were evaluated, the mutation frequencies of these genes were ranked from highest to lowest as *SOD1* (2.2%), *C9orf72* (2.1%), *ATXN2* (1.7%), *FUS* (1.7%), *TARDBP* (0.8%), *VCP* (0.6%), *UBQLN2*(0.6%) and *SQSTM1* (0.6%) in all the ALS patients.

**Conclusions:**

Our findings suggested that effective intervention for risk exposure and timely modification of lifestyle might prevent the occurrence of ALS. Genetic mutations are important risk factors for ALS and it is essential to detect genetic mutations correctly and scientifically.

**Systematic review registration:**

https://www.crd.york.ac.uk/PROSPERO/display_record.php?RecordID=301549, identifier: CRD42022301549.

## Introduction

Amyotrophic lateral sclerosis (ALS) is a fatal nervous system disease characterized by degeneration of upper and lower motor neurons, leading to severe muscle weakness and respiratory failure. It has a prevalence of 5 in 100,000, with an incidence of 2.6–3.0 cases per 100,000 people, reflecting a short average survival period (van Es et al., 2017). Approximately 5–10% of ALS patients have a family history (FALS), while the remaining cases are sporadic (SALS). Nowadays, although Riluzole and Edaravone approved by Food and Drug Administration, prolong survival for at least 3–5 months, no medications can halt the progression of ALS. Unfortunately, the etiology of ALS has not been fully uncovered so far.

Although cumulative evidence suggested genetic, environmental and aging factors have a potential role in the etiology of ALS, their respective part or exact mechanism in ALS was not largely unknown. To date, variants in more than 120 genes have been implicated in ALS (https://alsod.ac.uk). *C9ORF72, SOD1, FUS*, and *TARDBP* are the most common causative genes, accounting for 54.5% of FALS and 7.5% of SALS in Caucasians. However, the remaining cases, especially for most of SALS were not accounted for by variants of wellknown causative genes. As for environmental factors, many advanced epidemiologic studies have examined the potential risk factors for ALS, suggesting heavy metal exposure, pesticides, electromagnetic fields and lifestyle are also considered major associated factors for ALS (Zufiría et al., 2016). However, these studies for investigating the risk factors of ALS involved limited samples or elements, and the results usually can't be replicated with each other. Till now, the most comprehensive model for the risk factors of ALS was unavailable.

The risk factor or etiology of ALS has not yet been fully clarified. The gene-time-environment hypothesis and the multistep model suggest genetic predisposition interacts with environmental exposures over time leading to the development of ALS (Goutman et al., 2022). Therefore, analysis of genetic and non-genetic etiologies may shed light on the mechanism of diseases, provide new interventions and guidance for the prevention and prediction. Here, we will first conduct the most comprehensive analysis of genetic and non-genetic etiologies factors by a systematic review and meta-analysis of published data.

## Methods

### Systematic search

We adapt the recommendations and methods made by the Meta-analysis Of Observational Studies in Epidemiology (MOOSE) Group, the PRISMA 2009 guidelines for systematic review and meta-analysis (Stroup et al., 2000; Moher et al., 2009). We searched PubMed, Embase, and Cochrane Database for studies that reported genetic and non-genetic etiology of ALS from inception to Sep 2021. We searched the terms “Causality,” “Risk,” “cause,” “etiology,” “Superoxide Dismutase-1,” “*SOD1*,” “chromosome 9 open reading frame 72,” “*C9orf72*,” “TAR DNA binding protein,” “*TARDBP*,” “fused in sarcoma,” “*FUS*,” “Valosin-containing protein,” “*VCP*,” “Ubiquilin 2,” “*UBQLN2*,” “Sequestosome 1,” “*SQSTM1*,” “fused in sarcoma,” “*FUS*,” “ataxin-2,” “*ATXN2*,” combined with “amyotrophic lateral sclerosis” OR “ALS” OR “motor neuron(e) disease” OR “MND.” We also performed a hand search to search the bibliographies of relevant original studies more comprehensively if necessary. Two investigators (QQD, ZJ) performed literature selection, and if there were any discrepancies, the other two researchers (WMS, YPC) would determine the conclusion. A protocol was registered with PROSPERO https://www.crd.york.ac.uk/PROSPERO/display_record.php?RecordID=301549, registration number: CRD42022301549.

### Eligibility criteria and data collection

**Figure 1:**
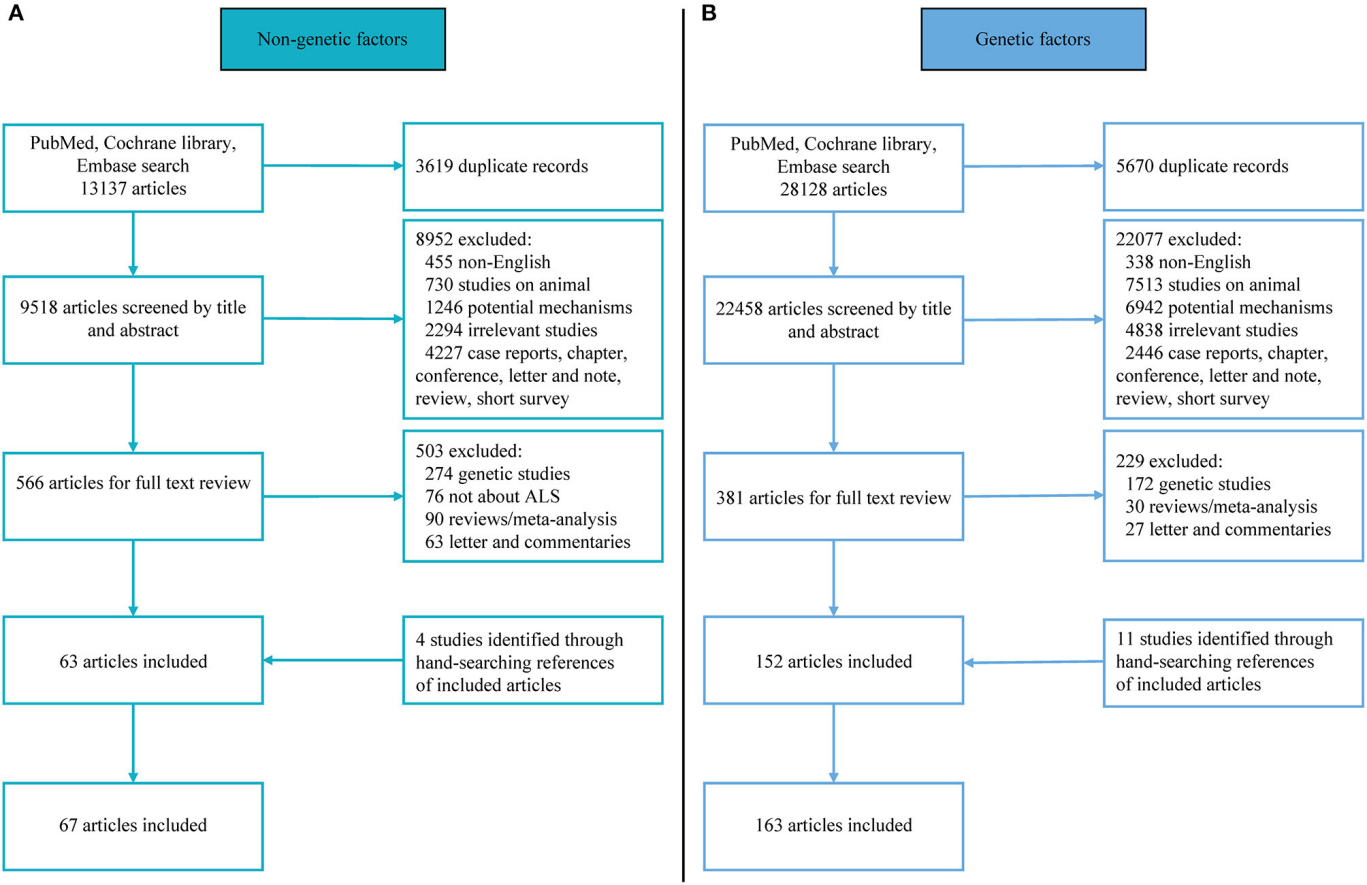
Flowchart of literature selection. **(A)** Flowchart of non-genetic literature selection; **(B)** flowchart of genetic literature selection.

Studies eligible for further review were clinical literature related to mutation/variants frequencies in *SOD1*, C9orf72, *TARDBP, FUS, UBQLN2, VCP, SQSTM1*, or *ATXN2* genes and non-genetic risk factors. Studies were included if (1) diagnostic criteria of ALS were clearly stated, either by clinical criteria, El Escorial criteria/revised El Escorial criteria or Awaji criteria; (2) the non-genetic studies reported original data concerning the odds ratio (OR) of ALS using a longitudinal cohort study or case-control study design, while the genetic study presented mutation frequencies of the candidate ALS genes using case-control studies or cohort study; (3) familial history of the patients with ALS enrolled was clear; (4) published in English. When the same patient group appeared in more than one publication, the group with the largest sample size or the latest publication date was used for the study of data extraction. When the number of articles about a particular research factor is less than three, this factor was excluded. The detailed flow chart is shown in Figure 1. All articles were carefully read by four independent review authors (QQD, ZJ, WMS or YPC) and were independently selected by each review author after inclusion on the basis of predefined criteria, with any discrepancies resolved by discussion.

### Data extraction and quality appraisal

For eligible non-genetic studies, the measurement and classification methods of exposures might be different among articles, data reporting exposures as multiple levels with a minimum level was equal to zero was qualitatively converted to dichotomy (such as maximum exposure and zero exposure/minimum exposure, once exposed and never exposed, yes and no exposure). For non-genetic studies, data were extracted independently by three authors (QQD, ZJ, and YPC) as follows: Name of the first author, publication year, country of the study, population/cohort, study design, diagnosis criteria, the sample size of ALS (FALS and/or SALS) cases and controls, numbers of different predefined exposures FALS and/or SALS cases and controls. For each eligible genetic study, data were extracted independently by three authors (QQD, WMS, and YPC) as follows: Name of the first author, publication year, country of the study, population, study design, diagnosis criteria, gene detection method, the sample size of FALS and/or SALS cases, numbers of mutation carriers in FALS and/or SALS. We use the Newcastle–Ottawa Scale (NOS) to assess the quality of articles (https://www.ohri.ca/programs/clinical_epidemiology/oxford.asp) (Stang, 2010) (Figure 1). Here, seven or more scores of NOS were considered as high quality, 5 and 6 scores as medium quality, and 4 or less as low quality. In the event of disagreement, the quality evaluators (QQD, WMS or ZJ) reached a consensus after consulting a fourth co-author (YPC).

### Statistical analysis

For eligible non-genetic studies, the primary outcome was an odds ratio (OR) with a 95% confidence interval (CI). To assess which factor had a greater impact on increasing the risk of ALS, we graded all non-genetic factors with the following criterion: OR ≥ 1.3 in risk factors or OR ≤ 0.7 in protective factors were defined as Class I and the rest was Class II for poor pathogenic factors (Su et al., 2021). Also, genetic studies resulted in the frequency of mutations in *SOD1, C9orf72, TARDBP, FUS, UBQLN2, VCP, SQSTM1* and *ATXN2* genes in all FALS or SALS cases with 95% confidence intervals (CI). Statistical heterogeneity between studies was assessed by Cochrane's Q test and I^2^ statistic, if p>0.05, it is considered that the test authenticity and reliability of this factor are high. If I^2^ <50%, we combined OR or mutation frequencies of the studies with a fixed-effects model. Otherwise, I^2^ ≥ 50% indicated substantial heterogeneity across studies, and a random-effects model was used (Higgins et al., 2003). We evaluated the robustness and credibility of the combined results by sensitivity analysis and subgroup analysis to check for potential heterogeneity. After excluding each study, the results were congruent, indicating that the result was reliable.

In addition, according to the previous study (Xu et al., 2015), we graded evidence as follows: “grade I evidence” was defined as both pooled population >5,000 and lower heterogeneity (I^2^ <50%); “grade II-A evidence” was defined as pooled population >5,000 but with higher heterogeneity (I^2^ ≥ 50%); “grade II-B evidence” was defined as lower heterogeneity (I^2^ <50%) but with pooled population <5,000; “grade III evidence” was defined as both pooled population <5,000 and higher heterogeneity.

## Results

### Systematic review

The literature search yielded 41,265 English language articles, 230 of which were eligible and entered into the meta-analysis, including 67 literature related to non-genetic factors and 163 articles that provided original information on mutation frequencies in eight candidate ALS genes. As for non-genetic risk, 22 factors were identified and pooled in analyzed. We summarized them in four sections, including lifestyle, environmental and occupational exposure, pre-existing diseases/comorbidity and medical exposure, and other non-genetic risk factors. Among these 67 included studies, 30 were from 2 or more centers and the most common study type was case-control. NOS scores indicated 47 were high-quality and 20 were medium-quality articles. As for genetic factors, about thirty-two of the studies were from two or more centers and NOS scores ranged from 5 to 8 and comprised 104 high-quality and 61 medium-quality articles.

### Meta-analysis

**Figure 2:**
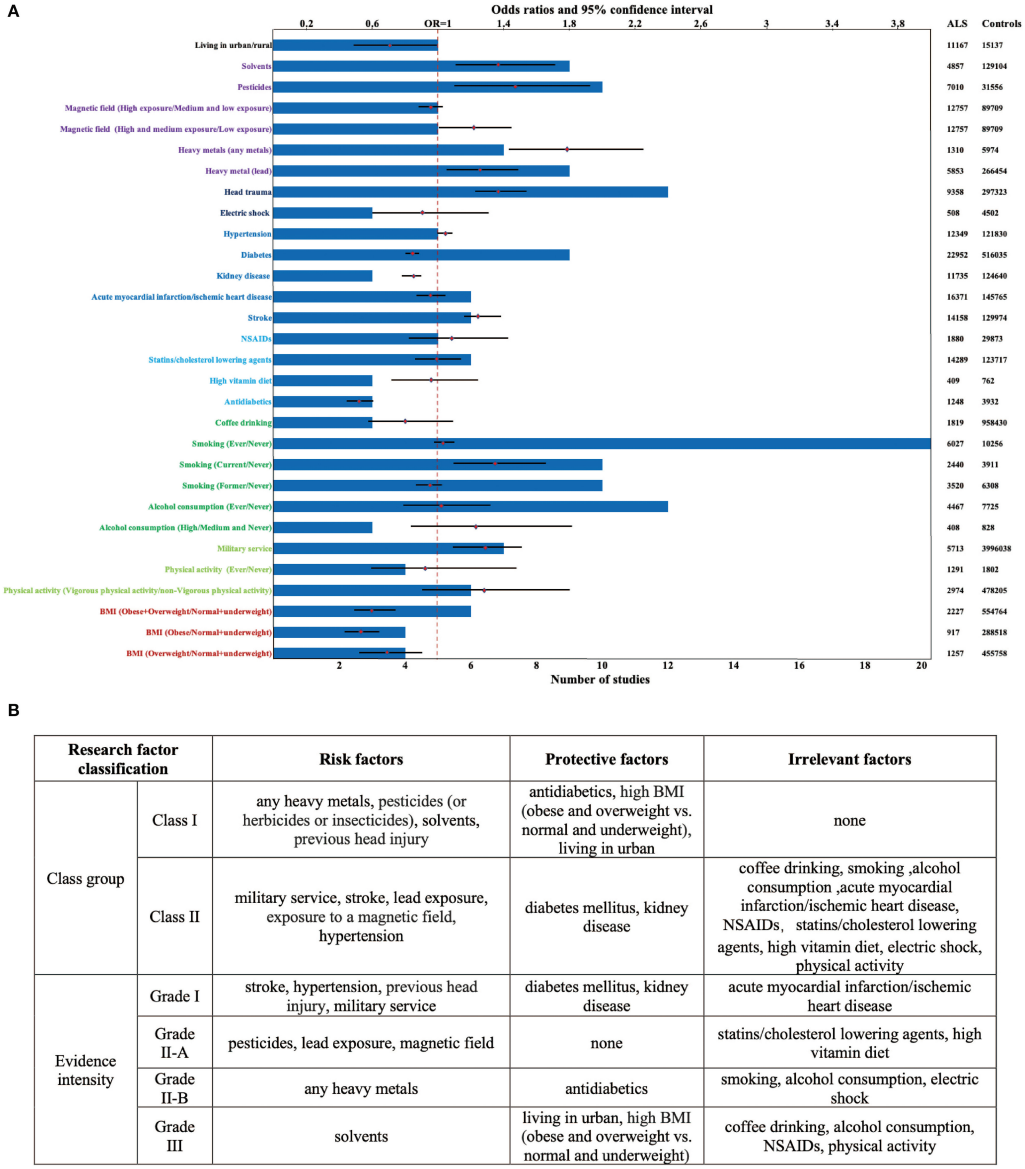
Factors showing significant positive and negative associations with ALS. **(A)** A total of 13 factors showed a trend of increasing the risk of ALS while a total of 7 factors showed a trend of decreasing the risk of ALS. 21 factors were included in more than or equal to 5 studies, and 14 research factors had the sample size of over 5,000; **(B)** the class group and evidence intensity of the ALS protective/risk factors.

For non-genetic factors, the summary findings for ALS that we found in the pooled analysis are shown in Table 1 and Figure 2A. Eight significant risk factors suggested are exposure to any heavy metals (OR = 1.79, 95% CI: 1.43 to 2.23, Class I, Grade II-B evidence), pesticides (or herbicides or insecticides) (OR = 1.46, 95% CI: 1.10 to 1.93, Class I, Grade II-A evidence), solvents (OR = 1.37, 95% CI: 1.10 to 1.70, Class I, Grade III evidence), previous head injury (OR = 1.37, 95% CI: 1.22 to 1.53, Class I, Grade I evidence), military service (OR = 1.29, 95% CI: 1.09 to 1.52, Class II, Grade I evidence), stroke (OR = 1.26, 95% CI: 1.15 to 1.38, Class II, Grade I evidence), lead exposure (OR = 1.25, 95% CI: 1.06 to 1.48, Class II, Grade II-A evidence), exposure to a magnetic field (OR = 1.22, 95% CI: 1.02 to 1.45, Class II, Grade II-A evidence) and hypertension (OR = 1.04, 95% CI: 1.00 to 1.08, Class II, Grade I evidence). However, five factors, including the use of antidiabetics (OR = 0.52, 95% CI: 0.45 to 0.61, Class I, Grade II-B evidence), high BMI (OR = 0.60 for Obese and Overweight vs. normal and underweight, 95% CI: 0.49 to 0.75, Class I, Grade III evidence), living in urban (OR = 0.70, 95% CI: 0.49 to 1.00, Class I, Grade III evidence), diabetes mellitus (OR = 0.83, 95% CI: 0.80 to 0.86, Class II, Grade I evidence), and kidney disease (OR = 0.84, 95% CI: 0.78 to 0.91, Class II, Grade I evidence), might decrease the risk of ALS (Supplementary Figures 1–4). No significant association for nine factors was found for smoking (Grade I evidence), acute myocardial infarction/ischemic heart disease (Grade I evidence), high vitamin diet (Grade II-A evidence), use of statins/cholesterol-lowering agents (Grade II-A evidence), electric shock (Grade II-A evidence), coffee drinking (Grade III evidence), alcohol consumption (Grade III evidence), use of NSAIDs (Grade III evidence) and physical activity (Grade III evidence) (Figure 2B; Supplementary Figures 1–4).

**Table 1:** Pooled odds ratio and heterogeneity statistics of positive and negative non-genetic associations with ALS.

**Protective/Risk factors**	**Number of studies**	**Case**	Mean onset age^*^	**Pooled OR(95%CI)**	**I^2^ (%)**	***P***
Living in urban	5	1,167	65.50 (NA)	0.70 (0.49, 1.00)	62.1	0.032
Diabetes	9	22,952	67.15 (61.9–74)	0.83 (0.80, 0.86)	26.1	0.212
Kidney disease	3	11,735	73.60 (66.2–74)	0.84 (0.78, 0.91)	0.0	0.643
Obese	4	917	62.53 (60–65.4)	0.53 (0.44, 0.64)	26.1	0.255
Overweight	4	1,257	65.40 (NA)	0.69 (0.52, 0.90)	63.3	0.043
Antidiabetics	3	1,248	60.71 (60–61.9)	0.52 (0.45, 0.61)	35.6	0.212
Solvents	10	4,857	63.23 (60–68)	1.37 (1.10, 1.70)	81.4	0.000
Pesticides	10	7,010	63.23 (60–68)	1.46 (1.10, 1.93)	78.9	0.000
Magnetic field^#^	5	12,757	64.4 (NA)	1.22 (1.02, 1.45)	87.7	0.000
Heavy metals	7	1,311	65.77 (63.7–68)	1.79 (1.43, 2.23)	46.3	0.083
Lead	9	5,853	62.10 (60-64.9)	1.25 (1.06, 1.48)	53.4	0.028
Head trauma	12	9,358	64.27 (62.4–68)	1.37 (1.22, 1.53)	0.0	0.629
Stroke	6	14,158	72.76 (61.9–74)	1.26 (1.15, 1.38)	42.8	0.120
Hypertension	3	12,349	73.08 (61.9–74)	1.04 (1.00, 1.08)	6.4	0.370
Smoking (current)	11	2,440	63.30 (60.0–68.0)	1.35 (1.10, 1.65)	45.5	0.049
Military service	7	5,713	62.77 (62.0–65.5)	1.29 (1.09, 1.52)	31.7	0.186

^*^ Year, range; ^#^ high and medium exposure; NA, unavailable.

For genetic factors, we calculated the mutation frequencies of *SOD1, TARDBP, FUS, C9orf72 SQSTM1, UBQLN2, VCP*, and *ATXN2* in all ALS patients, FALS and SALS separately. Forty-nine studies reported the frequency of *SOD1* mutations in 15,973 patients, in the pooled analysis, the mutation frequency was 2.2%. Thirty-nine studies reported the frequency of *C9orf72* repeat expansions in 23330 patients„ and the summarized frequency of *C9orf72* repeat expansions was 2.1%. Thirty-one studies reported the mutation frequency of *FUS* in 15,720 patients, and the calculated mutation frequency of *FUS* was 1.7%. In addition, the mutation frequency of *ATXN2* repeat expansions was 1.7% in 975,1 patients, of *TARDBP* was 0.8% in 11,744 patients, of *SQSTM1* reported in 6001 patients was 0.6%, of *UBQLN2* was 0.6% in 6,876 patients, and of *VCP* was 0.6% in 5,466 patients (Table 2; Supplementary Figure 5).

**Table 2:** Pooled mutated rate, subgroup analysis, and heterogeneity statistics of major ALS-related genes.

**Gene**	**Studies**	Mean age of onset^*^	**Mutation rate (95%CI)**	**I (%)**	**P (Q)**	**Category**	**Subgroup**	**Studies**	**Cases**	**Mutation rate (95%CI)**	**I (%)**	**P (Q)**
*SOD1*	49	49.39 ± 5.90	0.022 (0.020, 0.024)	96.0	0.000	Family history	FALS	41	3,008	0.083 (0.074, 0.092)	94.1	0.000
							SALS	39	12,965	0.014 (0.012, 0.016)	93.2	0.000
						Population	European	34	11,293	0.020 (0.017, 0.022)	97.0	0.000
							Asian	15	4,680	0.032 (0.027, 0.037)	82.7	0.000
*TARDBP*	29	57.31 ± 2.65	0.008 (0.006, 0.009)	80.9	0.000	Family history	FALS	22	1,670	0.037 (0.029, 0.046)	87.2	0.000
							SALS	25	10,065	0.006 (0.005, 0.008)	72.2	0.000
						Population	European	16	4,968	0.014 (0.011, 0.018)	82.9	0.000
							Asian	13	6,776	0.006 (0.004, 0.007)	63.1	0.000
*FUS*	31	41.90 ± 7.44	0.017 (0.013, 0.022)	88.7	0.000	Family history	FALS	28	2,678	0.032 (0.025, 0.038)	76.0	0.000
							SALS	27	13,042	0.014 (0.009, 0.018)	86.6	0.000
						Population	European	21	11,526	0.016 (0.012, 0.024)	90.8	0.000
							Asian	10	4,194	0.016 (0.012, 0.024)	80.5	0.000
*VCP*	13	56.02 ± 6.4	0.006 (0.002, 0.009)	58.8	0.019	Family history	FALS	10	1,233	0.014 (0.005, 0.023)	43.0	0.003
							SALS	11	4,233	0.006 (0.001, 0.011)	53.5	0.019
						Population	European	10	3,467	0.008 (0.003, 0.014)	73.2	0.003
							Asian	3	1,999	0.003 (0.001, 0.005)	0.0	0.016
*UBQLN2*	16	45.39 ± 12.05	0.006 (0.003, 0.008)	50.2	0.000	Family history	FALS	13	1,459	0.008 (0.003, 0.013)	0.0	0.001
							SALS	14	5,471	0.006 (0.003, 0.008)	52.0	0.000
						Population	European	12	4,400	0.007 (0.004, 0.010)	61.2	0.000
							Asian	4	2,530	0.003 (0.001, 0.005)	0.0	0.008
*SQSTM1*	14	57.58 ± 4.78	0.006 (0.004, 0.007)	83.7	0.000	Family history	FALS	11	1,429	0.010 (0.004, 0.015)	63.9	0.079
							SALS	11	4,572	0.005 (0.003, 0.007)	84.7	0.000
						Population	European	10	3,721	0.006 (0.003, 0.008)	86.9	0.000
							Asian	4	2,280	0.006 (0.003, 0.008)	53.4	0.001
*C9orf72*	39	57.46 ± 5.50	0.021 (0.019, 0.022)	97.4	0.000	Family history	FALS	33	6,328	0.059 (0.054, 0.064)	96.3	0.000
							SALS	35	17,002	0.013 (0.011, 0.014)	96.5	0.000
						Population	European	25	19,093	0.086 (0.065, 0.106)	96.3	0.000
							Asian	14	5,171	0.019 (0.011, 0.026)	80.3	0.000
*ATXN2*	14	56.02 ± 4.89	0.017 (0.015, 0.020)	79.8	0.000	Family history	FALS	12	987	0.029 (0.016, 0.041)	2.0	0.057
							SALS	18	8,764	0.017 (0.015, 0.020)	71.0	0.000
						Population	European	11	6,393	0.019 (0.016, 0.022)	79.9	0.000
							Asian	7	3,358	0.015(0.011, 0.019)	46.4	0.000

^*^Mean onset age of patients with variants in each gene, years, mean ± SD.

### Heterogeneity

Between-study heterogeneity was calculated using the I^2^ statistic. In non-genetic studies, substantial heterogeneity existed in studies exposed to solvents, pesticides, any heavy metals, lead, previous head injury, stroke, military service, magnetic field, hypertension, diabetes mellitus, use of antidiabetics, high BMI, living in urban/rural, kidney disease, use of statins/cholesterol-lowering agents, use of NSAIDs, acute myocardial infarction/ischemic heart disease, high vitamin diet, coffee drinking, smoking and electric shock. In genetic studies, substantial heterogeneity (I^2^: 50.2–96.5%, *p* < 0.05) existed in studies screening *SOD1, TARDBP, FUS, C9orf72, SQSTM1, UBQLN2, VCP*, and *ATXN2* mutations.

### Sensitivity analysis and publication bias

When the included literature was ≥5, Begg's test was used to further publication bias (Supplementary Figures 9–13). We concluded that there was no publication bias for study, as the results of Begg's test suggested that the *p*-values for all study factors were >0.05. To assess the impact of each study on the overall results, we performed a sensitivity analysis for both genetic and non-genetic factors. For studies reporting pesticide exposure, sensitivity analysis showed that when the study of Peters et al. (2017) was omitted, the study heterogeneity was greatly reduced (I^2^ decreased to 33.5%). For studies reporting living in urban, sensitivity analysis showed that when the study of Korner et al. (2019) was omitted, the study heterogeneity was greatly reduced (I^2^ decreased to 47.0%). For lead exposure, sensitivity analysis showed that when the study of Andrew et al. (2017) and Peters et al. (2017) was omitted, I^2^ was greatly decreased to 49.1 and 37.2% respectively, The above results suggest that these studies may lead to unstable results. We did not find that the heterogeneity could be significantly reduced by removing any literature on genetic studies, indicating that the result was reliable and stable (Supplementary Figures 14–18).

### Subgroup analysis

We performed a subgroup analysis to analyze whether the OR differed across populations or exposure conditions (Supplementary Figures 1, 2, 4). The subgroup analysis showed, compared with never smoking, an increased risk for current smoking (OR = 1.35, 95% CI: 1.10 to 1.65, Class I, Grade II-B evidence), rather than exposure to former (OR = 0.95, 95% CI: 0.87 to 1.03, Class II, Grade II-B evidence), suggesting quitting is a protective behavior against ALS. In addition, compared with low dose exposure to the magnetic field, high and medium dose exposure will increase the risk of ALS (OR = 1.22, 95% CI: 1.02 to 1.45, Class II, Grade II-A evidence). In contrast, compared with normal and underweight, a decreased risk for obesity (OR = 0.53, 95% CI: 0.44 to 0.64, Class I, Grade II-B evidence) and overweight (OR = 0.69, 95% CI: 0.52 to 0.90, Class I, Grade III evidence), further supported a potential protective association between high BMI and ALS risk. However, we performed a subgroup analysis on the dose of alcohol consumption and the intensity of physical activity, we did not find any significant changes in the relationship between the exposure factors mentioned above and the incidence of ALS.

For genetic factors, the pooled mutation frequencies in European were ranked from highest to lowest as *C9orf72* repeat expansions (8.6%), *SOD1* (2.0%), *ATXN2* repeat expansions (1.9%), *FUS* (1.6%),*TARDBP* (1.4%), *VCP* (0.8%), *UBQLN2* (0.7%), *SQSTM1* (0.6%), while in Asian populations was *SOD1*(3.2%), *C9orf72* repeat expansions (1.9%), *ATXN2* repeat expansions (1.5%), *FUS* (1.6%), *TARDBP* (0.6%), *SQSTM1* (0.6%), *UBQLN2* (0.3%) and *VCP* (0.3%). In addition, we summarized the mutation frequencies of FALS and SALS. In FALS, the most common gene was *SOD1* (8.3%, analyzed 3,008 patients), followed by *C9orf72* repeat expansions (5.9%, analyzed 6,328 patients), *FUS* (4.6%, analyzed 2,678 patients), *TARDBP* (3.7%, analyzed 1,670 patients), *ATXN2* repeat expansions (2.9%, analyzed 987 patients), *VCP* (1.4%, analyzed 1,233 patients), *SQSTM1* (1.0%, analyzed 1,429 patients) and *UBQLN2* (0.8%, analyzed 1,459 patients). In contrast, In SALS, the most common gene was *ATXN2* repeat expansions (1.9%, 6,975 patients), followed by *SOD1* (1.4%, 12,965 patients), *FUS* (1.4%, 13,042 patients), *C9orf72* repeat expansions (1.3%, 17,002 patients), *TARDBP* (0.6%, 10,065 patients), *UBQLN2* (0.6%, 5,471 patients) *VCP* (0.6%, 4,233 patients) and *SQSTM1* (0.5%, 4,572 patients) (Supplementary Figures 6–8).

### Age in non-genetic factors and genetic factors

**Figure 3:**
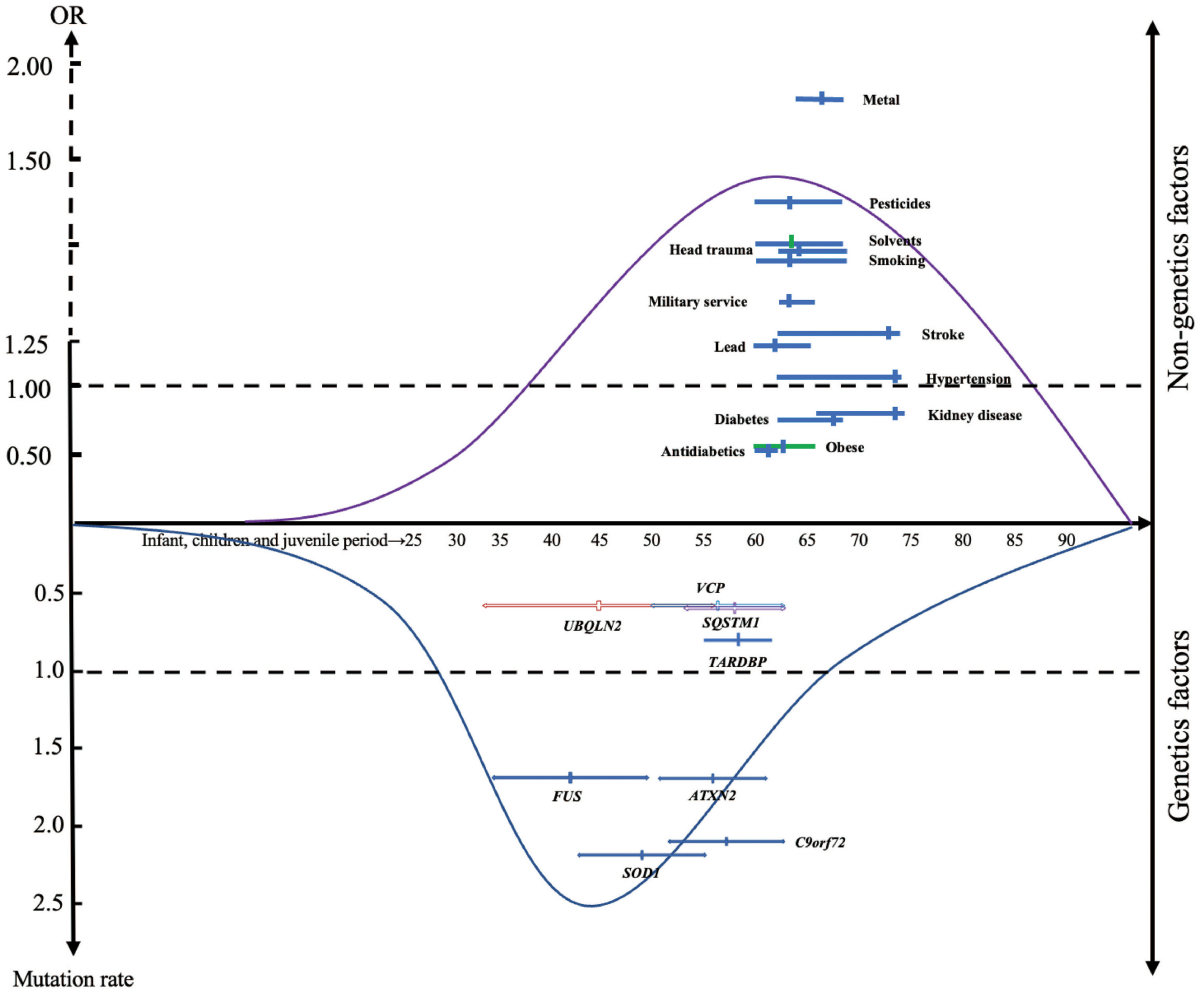
The distribution of modifiable factors throughout the course of life. Suggesting that genetic factors may have a more important causative role in patients with early onset of disease than non-genetic factors. The x-axis represents the mean age of the total sample (solid circle), including the mean age range of the included studies (short horizontal line). The y-axis represents the summarized odds ratio (OR) and mutation frequency.

We discussed the mean age of onset of the study population for each factor, results showed that the average age of onset in patients with gene mutation and exposed to environmental risk factors were 41.90 to 57.46 and 62.53 to 73.60, respectively (Figure 3). As expected, patients with ALS-related gene mutations tend to have a younger age of onset compared with those exposed to environmental factors.

## Discussion

Till now, the etiology of ALS is largely unknown. As far as we know, this is the most comprehensive meta-analysis investigating non-genetic and genetic risk factors of ALS patients. 22 non-genetic factors, including lifestyle, pre-existing diseases/comorbidity and medical exposure, environmental and occupational exposure, and other non-genetic risk factors, were analyzed. We found 8 of which significantly increase the risk of future ALS development and 5 of which decrease the incidence of ALS. In addition, we also comprehensively summarized the mutation frequencies of ALS patients, including different ethnic and whether they have family history.

### Non-genetic factors

Previous studies reported the link between lifestyle and ALS; however, conflicting evidence was found. Here, we analyzed three common lifestyles, including smoking, alcohol consumption and coffee drinking. We found alcohol consumption, coffee drinking and smoking might not be associated with a higher incidence of ALS, but the findings need to be further determined due to sample size and heterogeneity. Then, a subgroup analysis of smoking and drinking was performed to resolve this partially. The results of the subgroup analysis showed that current smoking significantly increased the risk of ALS compared with previous smoking, emphasizing the favorable role of quitting smoking in the prevention of ALS. But our subgroup analysis did not find a significant correlation between different drinking doses and ALS incidence, which was confirmed in the previous study (D'Ovidio et al., 2019).

Now, environmental and occupational exposures should be the most concern of non-genetic factors linked to ALS in the last several decades (Koeman et al., 2017; Dickerson et al., 2019; Gunnarsson and Bodin, 2019; Meng et al., 2020). Here, we focused on exposure to solvents, electromagnetic fields, pesticides, and metals. Results indicated pesticides, heavy metals and solvents, were significant positively proportional to the risk of ALS in Class I, while lead exposure was a mild risk factor in Class II. Previous studies have shown that solvents and pesticides are neurotoxic (Baker, 1988; Dick, 2006), and are associated with cognitive impairment and neurodegenerative diseases (Mostafalou and Abdollahi, 2018; Sharma et al., 2020). Our research also reached these conclusions. In terms of electromagnetic field exposure, it has begun to be evaluated as a potential risk factor for neurodegeneration (Riancho et al., 2021). By the pooled analysis, we found no significant association between electromagnetic fields and the incidence of ALS, except the dose of exposure might affect the development of ALS. Heavy metals, such as lead, mercury and selenium are epidemiologically linked with ALS risk, which was also consistent with the current finding lead increased risk for ALS, and the homeostasis of TDP-43 in neurons was disrupted might be one of the mechanisms of metals causing neurodegeneration (Ash et al., 2019; Koski et al., 2021).

Considering that ALS is one of the devastating and incurable diseases, the relationship disclosure between pre-existing disease states, comorbidity, medical exposures and ALS will provide clues whether these diseases should be treated when a patient is diagnosed with ALS, and medical exposures with modifiable risk factors would be promising approaches for ALS. Here, the common chronic conditions, such as diabetes, hypertension and stroke, and the common oral drugs, including statins/cholesterol-lowering drugs, NSAIDs and antidiabetic drugs are analyzed. In our meta-analysis, our study shows head trauma, stroke, and hypertension were risk factors. Many studies have evaluated the correlations between head trauma and ALS risk (Andrew et al., 2021; Gu et al., 2021; Liu et al., 2021), and a history of head trauma was associated with ALS development. A study investigated the effects of head trauma on pathology using rats and drosophila as research models showed that brain trauma leads to defects in nucleocytoplasmic transport, which may mediate TDP-43 pathology in neurodegenerative diseases (Anderson et al., 2021). We also found a slight but significant positive association between stroke, hypertension and ALS, which indicated stroke or hypertension might affect the development or prognosis for ALS. Conversely, diabetes was a modest protective factor, which was consistent with previous studies that demonstrated a correlation between diabetes and delayed onset of ALS (Schumacher et al., 2020; Chen et al., 2021; Ferri et al., 2021), supporting the hypothesis that metabolic disturbances may contribute to the clinical progression of ALS. Meanwhile, the use of antidiabetics was a decisive factor in the prevention of ALS, which may be influenced by the oral hypoglycemic drugs taken by diabetic patients. Kidney disease also had a protective effect on the occurrence of ALS as diabetes. However, few studies formed the relationship between nephropathy and ALS, and this field still needs to be further explored. Here, all pre-existing disease or comorbidity states analyzed in this study were Grade I evidence, use of statins/cholesterol-lowering drugs was Level II-A evidence, use of antidiabetic medications and a high vitamin diet was Grade II-B, and use of NSAID was Grade III evidence.

In addition, for other non-genetic factors, our analysis results supported the roles of military service in ALS occurrence. Our study also confirmed that high BMI was a protective factor for ALS onset, and subgroup analysis further showed obesity has a more substantial protective effect on ALS than overweight, supporting weight loss was strongly associated with shorter survival in ALS patients (Peter et al., 2017; Nakken et al., 2019; Shimizu et al., 2019; Diekmann et al., 2020; Su et al., 2021). Finally, living in urban was found to have a mild protective effect on ALS. Possible reasons include little exposure to solvents, pesticides, and metals for people living in urban areas compared with rural areas. But the pooled population of the part is relatively small, so further studies are needed to unveil possible mechanisms and clinical consequences of this finding.

Our study suggests that a history of head trauma, current smoking, exposure to solvents, pesticides and heavy metals are strong risk factors for ALS (OR ≥ 1.3), and we need to be alert to the occurrence of ALS in groups exposed to these factors over long periods of time, especially exposure to multiple risk factors. And we are in favor of better nutrition and moderate obesity in ALS patients, which may have a protective effect on the disease. Prospective studies with large samples will be necessary in the future for a more precise identification of the risk factors for ALS.

### Genetic factors

Although genetic factors play a limited role in the development of ALS, they could essentially reveal the mechanism of the disease. Here, by the pooled analysis, the candidate seven ALS causative genes accounted for about 10% of ALS in all the patients, and the mutation frequencies were more in European patients or FALS, suggesting genetic factors should not be ignored.

Our analysis showed that the overall pooled mutation frequencies of these major ALS-related genes are 2.2% for *SOD1*, 2.1% for *C9orf72*, 1.7% for *ATXN2*, 1.7% for *FUS*, 0.8% for *TARDBP*, 0.6% for *VCP*, 0.6% for *SQSTM1* and 0.6% for *UBQLN2*. Subgroup analysis of whether there was a family history of ALS, and several conclusions could be drawn from the results. The mutation frequencies of these eight major ALS-related genes are 28.6% in FALS and 8.1% in SALS. We can conclude that family ALS has a higher gene mutation rate than sporadic ALS, suggesting that gene mutation might be one of the important risk factors and gene detection is of great significance in FALS. In FALS patients, the top three mutated genes are *SOD1, C9orf72*, and *FUS*, which are *ATXN2, SOD1* and *FUS* in SALS. Our findings emphasize the distinct genetic structure between FALS and SALS, which need to be given appropriate consideration when performing genetic testing on patients with ALS.

Subgroup analysis also showed the difference in gene mutation results between European and Asian populations. In European populations, the most common mutations in ALS were the *C9orf72* repeats, followed by *SOD1, ATXN2* repeats, FUS, *TARDBP, VCP, UBQLN2* and *SQSTM1*, In Asian populations, the most common mutations in ALS were the *SOD1*, followed by *C9orf72* repeats, *FUS, ATXN2* repeats, *SQSTM1, TARDBP, UBQLN2*, and *VCP*. Our results suggested that gene mutations are more common in European than Asians, and the mutant spectrum in different populations is significant differences.

### Limitation

Several limitations in this meta-analysis should be noted. First, most of the literature included in the present meta-analysis was case-control, without prior interventions in the experimental and control groups, making it difficult to quantitatively evaluate and scientifically categorize exposure levels for monitoring. Second, it was difficult to ensure that each study patient was exposed to a single risk factor, and not all studies made adequate adjustments for confounders. Third, the number of pooled populations included was small for some risk factors, resulting in low reliability and evidence. Fourth, we only included English-language literature, and all of them were from the above-mentioned databases. Finally, we only assessed risk factors for which the number of literature searches was ≥ 3, and there may be many risk factors that we did not discuss.

## Conclusion

In conclusion, our findings emphasized the effect of lifestyle, environmental and occupational exposure, pre-existing disease/comorbidity and medical exposure, and other non-genetic risk factors. At present, the replicable and definitive non-genetic risk factors of ALS have not been identified. In addition, we found diversity in mutation rates across family histories and populations from different ethnic. Our findings support the future assessment of these non-genetic and genetic factors as potential etiologic contributors to the risk of ALS, while the relationship between phenotype, genetics, and pathology remains to be further investigated.

## Data availability statement

The original contributions presented in the study are included in the article/Supplementary material, further inquiries can be directed to the corresponding author/s.

## Author contributions

Q-QD, BC, and Y-PC conceived and designed the study. Q-QD, ZJ, W-MS, X-JG, and Y-PC selected the articles and extracted and cross-checked the data. Q-QD, HW, X-JG, and Y-FC contributed to the statistical analysis. Q-QD and Y-PC wrote the first draft of the manuscript. Q-QD, XG, YW, and Y-PC revised and discussed the final edition. All authors contributed to the article and approved the submitted version.
