# Supplementary material for: Risk factors of amyotrophic lateral sclerosis: a global meta-summary
Source: Front Neurosci. 2023 Apr 24;17:1177431. doi: 10.3389/fnins.2023.1177431 (PMC10165003; doi:10.3389/fnins.2023.1177431)
Supplement: Supplementary file 1 [file Data_Sheet_1.docx]

**Supplementary Tables**

**Catalog**

| **Result** |  |  |
| --- | --- | --- |
| 1.Frequency of causative/risk genes by subgroup | Supp. Table 1 | Page 2 |
| **Features of included literature and Quality Evaluation** | | |
| 1. Non-genetic articles’ features and their NOS score 2. Genetic articles’ features and their NOS score | Supp. Table 2-24  Supp. Table 25-32 | page 3-25  page 26-34 |
| **References** |  | page 35-45 |

**Supplementary Table 1 the mutation frequencies of the main causative/risk genes in patients by subgroups**

| Genes | Number of Studies | Cases | Mutation frequencies (95% CI) | p(Q) | I² (%) |
| --- | --- | --- | --- | --- | --- |
| **Family history** |  |  |  |  |  |
| FALS |  |  |  |  |  |
| *SOD1* | 41 | 3008 | 0.083(0.074,0.092) | 0.000 | 94.1 |
| *TARDBP* | 22 | 1670 | 0.037 (0.029,0.046) | 0.000 | 87.2 |
| *FUS* | 28 | 2678 | 0.032 (0.025,0.038) | 0.000 | 76.0 |
| *VCP* | 10 | 1233 | 0.014 (0.005,0.023) | 0.003 | 43.0 |
| *UBQLN2* | 13 | 1459 | 0.008 (0.003,0.013) | 0.001 | 0.00 |
| *SQSTM1* | 11 | 1429 | 0.010 (0.004,0.015) | 0.079 | 63.9 |
| *C9orf72* | 33 | 6328 | 0.059 (0.054,0.064) | 0.000 | 96.3 |
| *ATXN2* | 12 | 987 | 0.029 (0.016,0.041) | 0.057 | 2.0 |
| SALS |  |  |  |  |  |
| *SOD1* | 39 | 12965 | 0.014 (0.012,0.016) | 0.000 | 93.2 |
| *TARDBP* | 25 | 10065 | 0.006 (0.005,0.008) | 0.000 | 72.2 |
| *FUS* | 27 | 13042 | 0.014 (0.009,0.018) | 0.000 | 86.6 |
| *VCP* | 11 | 4233 | 0.006 (0.001,0.011) | 0.019 | 53.5 |
| *UBQLN2* | 14 | 5471 | 0.006 (0.003,0.008) | 0.000 | 52.0 |
| *SQSTM1* | 11 | 4572 | 0.005 (0.003,0.007) | 0.000 | 84.7 |
| *C9orf72* | 35 | 17002 | 0.013 (0.011,0.014) | 0.000 | 96.3 |
| *ATXN2* | 18 | 8764 | 0.017(0.015,0.020) | 0.000 | 71.0 |
| **Cohort** |  |  |  |  |  |
| European |  |  |  |  |  |
| *SOD1* | 34 | 11293 | 0.020 (0.017,0.022) | 0.000 | 97.0 |
| *TARDBP* | 16 | 4968 | 0.014 (0.011,0.018) | 0.000 | 82.9 |
| *FUS* | 21 | 11526 | 0.016 (0.012,0.024) | 0.000 | 90.8 |
| *VCP* | 10 | 3467 | 0.008 (0.003,0.014) | 0.003 | 73.2 |
| *UBQLN2* | 12 | 4400 | 0.007 (0.004,0.010) | 0.000 | 61.2 |
| *SQSTM1* | 10 | 3721 | 0.006 (0.003,0.008) | 0.000 | 86.9 |
| *C9orf72* | 25 | 19093 | 0.086 (0.065,0.106) | 0.000 | 96.3 |
| *ATXN2* | 11 | 6393 | 0.019 (0.016,0.022) | 0.000 | 79.9 |
| Asian |  |  |  |  |  |
| *SOD1* | 15 | 4680 | 0.032 (0.027,0.037) | 0.000 | 82.7 |
| *TARDBP* | 13 | 6776 | 0.006 (0.004,0.007) | 0.000 | 63.1 |
| *FUS* | 10 | 4194 | 0.016 (0.012,0.024) | 0.000 | 80.5 |
| *VCP* | 3 | 1999 | 0.003 (0.001,0.005) | 0.016 | 0.00 |
| *UBQLN2* | 4 | 2530 | 0.003 (0.001,0.005) | 0.008 | 0.00 |
| *SQSTM1* | 4 | 2280 | 0.006 (0.003,0.008) | 0.000 | 53.4 |
| *C9orf72* | 14 | 5171 | 0.019 (0.0110.026) | 0.000 | 80.8 |
| *ATXN2* | 7 | 3358 | 0.015 (0.011,0.019) | 0.000 | 46.4 |

**
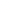
**

**Supplementary Table 2 the information of studies investigating solvents for ALS involved this study**

| First author | Publish year | Population | Study design | Diagnostic category | Number of centers | Exposure | |  | Age (years) | | NOS |
| --- | --- | --- | --- | --- | --- | --- | --- | --- | --- | --- | --- |
|  |  |  |  |  |  | Cases (Y/N） | Controls（Y/N） |  | Cases | Controls |  |
| McGuire, V ^(1)^ | 1997 | USA: WWS | Case-control study | Clinical diagnosis | 3 | 114/57 | 196/152 |  | NA | NA | 8 |
| Fang, F ^(2)^ | 2009 | USA: NE* | Case-control study | El Escorial criteria | 2 | 13/96 | 28/225 |  | Med: 60 | Med: 59 | 7 |
| Furby, A ^(3)^ | 2010 | France | Case-control study | El Escorial criteria | 1 | 19/89 | 23/99 |  | Med: 68 | Med:65 | 7 |
| Das, K ^(4)^ | 2012 | India | Case-control study | El Escorial criteria | 1 | 0/110 | 0/240 |  | NA | NA | 6 |
| Pamphlett, R. ^(5)^ | 2012 | Australia | Case-control study | El Escorial criteria | 6 | 332/282 | 287/491 |  | NA | NA | 7 |
| Andrew, A. S. ^(6)^ | 2017 | USA: NE* | Case-control study | El Escorial criteria | 2 | 64/198 | 26/169 |  | Med: 62 | Med: 61 | 7 |
| Koeman, T ^(7)^ | 2017 | Netherlands | Cohort study | ICD-9 and ICD-10 | 1 | 18/118 | 375/3791 |  | NA | NA | 7 |
| Peters, T. L. ^(8)^ | 2017 | Swedish | Case-control study | ICD-9 and ICD-10 | 1 | 489/1787 | 2496/9104 |  | NA | NA | 6 |
| Filippini, T. ^(9)^ | 2020 | Italy | Case-control study | El Escorial criteria | 4 | 44/51 | 50/85 |  | Mean:64.9 | Mean:66.5 | 7 |
| [Andrea Bellavia](https://pubmed.ncbi.nlm.nih.gov/?term=Bellavia+A&cauthor_id=33130429)^(10)^ | 2021 | Denmark | Case-control study | ICD-8 and ICD-10 | 1 | 446/640 | 42962/68545 |  | NA | NA | 6 |

WWS: western Washington State; NE: New England; Med: median; Y, yes; N, no; *different centers

**Supplementary Table 3 the information of studies investigating pesticides for ALS involved this study**

| First author | Publish year | Countries | Study design | Diagnostic category | Number of centers | Exposure | |  | Age (years) | | NOS |
| --- | --- | --- | --- | --- | --- | --- | --- | --- | --- | --- | --- |
|  |  |  |  |  |  | Cases (Y/N） | Controls（Y/N） |  | Cases | Controls |  |
| McGuire, V ^(1)^ | 1997 | USA: WWS | Case-control study | Clinical diagnosis | 3 | 33/138 | 48/300 |  | NA | NA | 8 |
| Fang, F ^(2)^ | 2009 | USA: NE | Case-control study | El Escorial criteria | 2 | 15/94 | 29/224 |  | Med: 60 | Med: 59 | 7 |
| Furby, A. ^(3)^ | 2010 | France | Case-control study | El Escorial criteria | 1 | 17/91 | 7/115 |  | Med: 68 | Med:65 | 7 |
| Das, K. ^(4)^ | 2012 | India | Case-control study | El Escorial criteria | 1 | 56/54 | 108/132 |  | NA | BA | 6 |
| Pamphlett, R. ^(5)^ | 2012 | Australia | Case-control study | El Escorial criteria | 6 | 416/198 | 432/346 |  | NA | NA | 7 |
| Peters, T. L. ^(8)^ | 2017 | Swedish | Case-control study | ICD-9 and ICD-10 | 1 | 355/4665 | 1912/23198 |  | NA | NA | 6 |
| Andrew, A. S. ^(6)^ | 2017 | USA: NE* | Case-control study | El Escorial criteria | 2 | 34/228 | 8/187 |  | Med: 62 | Med: 61 | 6 |
| Koeman, T. ^(7)^ | 2017 | Netherlands | Cohort study | ICD-9 and ICD-10 | 1 | 5/131 | 196/3970 |  | NA | NA | 7 |
| Filippini, T. ^(9)^ | 2020 | Italy | Case-control study | El Escorial criteria | 4 | 21/74 | 25/110 |  | Mean:64.9 | Mean:66.5 | 7 |
| Stephen A. Goutman^(11)^ | 2022 | USA | Case-control study | El Escorial criteria | 1 | 83/298 | 41/231 |  | Mean:63.0 | Mean:61.2 | 7 |

WWS: western Washington State; NE: New England; Med: median; Y, yes; N, no; *different centers

**Supplementary Table 4 the information of studies investigating magnetic field for ALS involved this study**

| First author | Publish year | Population | Study design | Diagnostic category | Number of centers | Exposure | |  | Age(years) | | NOS |
| --- | --- | --- | --- | --- | --- | --- | --- | --- | --- | --- | --- |
|  |  |  |  |  |  | Cases (H/M/L） | Controls (H/M/L） |  | Case | Control |  |
| Ximena Vergara ^(12)^ | 2014 | USA | Case-control study | ICD-10 and ICD-9 | 1 | 503/4748/635 | 5171/45517/6979 |  | NA | NA | 5 |
| Heidi Fischer ^(13)^ | 2015 | Sweden | Case-control study | ICD-10 and ICD-9 | 3 | 499/1938/2272 | 2688/9418/11229 |  | NA | NA | 6 |
| Koeman, T. ^(7)#^ | 2017 | Netherlands | Cohort study | ICD-9 and ICD-10 | 1 | 10/68/58 | 180/1939/2047 |  | NA | NA | 6 |
| Vinceti, M. ^(14)^ | 2017 | Italy | Case-control study | Clinic diagnose | 2 | 4/2/697 | 23/12/2702 |  | NA | NA | 8 |
| Peters.S. ^(15) #^ | 2019 | Ireland, Italy, Netherlands | Case-control study | ICD-10 and ICD-9 | 3 | 93/552/678 | 142/1015/1547 |  | Mean:64.4 | Mean:64.0 | 7 |
| Filippini, T. ^(9)^ * | 2020 | Italy | Case-control study | El Escorial criteria | 4 | 13/82 | 12/123 |  | Mean:64.9 | Mean:66.5 | 7 |

H, high exposure; M, medium exposure; L, low exposure, * exposure, yes/no. This study was excluded in meta-analysis; ^#^ part of population were overlap; low exposure was reference.

**Supplementary Table 5 the information of studies investigating heavy metals for ALS involved this study**

| First author | Publish year | Population | Study design | Diagnostic category | Number of centers | Exposure | |  | Age (Years) | | NOS |
| --- | --- | --- | --- | --- | --- | --- | --- | --- | --- | --- | --- |
|  |  |  |  |  |  | Cases (Y/N) | Controls(Y/N) |  | Cases | Controls |  |
| McGuire, V ^(1)^ | 1997 | USA: WWS | Case-control study | Clinical diagnosis | 3 | 84/87 | 139/209 |  | NA | NA | 8 |
| Alessandra Binazzi ^(16)^ | 2009 | Italy | Case-control study | The revised El Escorial criteria | 4 | 9/68 | 7/178 |  | Mean:63.7 | NA | 7 |
| Alain Furby ^(3) #^ | 2009 | France | Case-control study | The revised El Escorial criteria | 1 | 9/99 | 13/109 |  | Med: 68 | Med:65 | 7 |
| Pamphlett, R. ^(5)^ | 2012 | Australia | Case-control study | El Escorial criteria | 6 | 65/549 | 38/740 |  | NA | NA | 7 |
| Das, K. ^(4)^ | 2012 | India | Case-control study | El Escorial criteria | 1 | 3/107 | 6/234 |  | NA | NA | 6 |
| Koeman, T. ^(7)^ | 2017 | Netherlands | Cohort study | ICD-9 and ICD-10 | 1 | 13/123 | 266/3900 |  | NA | NA | 7 |
| Tommaso Filippini^(9)^ | 2020 | Italy | Case-control study | El Escorial criteria | 4 | 25/70 | 13/122 |  | Mean:64.9 | Mean:66.5 | 7 |

^#^ Mineral substances exposure; only reported single metals were excluded.

**Supplementary Table 6 the information of studies investigating heavy metal (lead) for ALS involved this study**

| First author | Publish year | Population | Study design | Diagnostic category | Number of centers | Exposure rate | |  | Age | | NOS |
| --- | --- | --- | --- | --- | --- | --- | --- | --- | --- | --- | --- |
|  |  |  |  |  |  | Cases (n/N） | Controls（n/N） |  | Cases | Controls |  |
| McGuire, V ^(1)^ | 1997 | USA: WWS | Case-control study | Clinical diagnosis | 3 | 21/153 | 24/324 |  | NA | NA | 8 |
| Fang, F ^(2)^ | 2009 | USA: NE* | Case-control study | El Escorial criteria | 2 | 35/74 | 55/198 |  | Med: 60 | Med: 59 | 7 |
| Angeline S. Andrew ^(6)^ | 2017 | USA: NE* | Case-control study | El Escorial criteria | 2 | 33/229 | 9/184 |  | Med: 62 | Med: 61 | 7 |
| Tracy L Peters ^(8)$^ | 2017 | Sweden | Case-control study | ICD-9 and ICD-10 | 1 | 611/1787 | 3052/9104 |  | NA | NA | 6 |
| A.S Dickerson ^(17)@^ | 2018 | Five Nordic countries^#^ | Case-control study | ICD-8 and ICD-10 | 5 | 171/1322 | 14462/123842 |  | NA | NA | 5 |
| Tommaso Filippini ^(9)^ | 2020 | Italy | Case-control study | El Escorial criteria | 4 | 23/72 | 23/122 |  | Mean:64.9 | Mean:66.5 | 7 |
| Susan Peters ^(18)^ | 2020 | Seven European counties^&^ | Case-control study | ICD-10 | 10 | 41/66 | 109/210 |  | Med:60.4 | Med:60.5 | 7 |
| [Andrea Bellavia](https://pubmed.ncbi.nlm.nih.gov/?term=Bellavia+A&cauthor_id=33130429)^(10)^ | 2021 | Denmark | Case-control study | ICD-8 and ICD-10 | 1 | 32/1054 | 3164/108343 |  | NA | NA | 6 |
| Hiroshi Mitsumoto^(19)^ | 2022 | USA | Case-control study | Clinical diagnosis | 1 | 27/68 | 13/52 |  | Mean:63.4 | Mean:63.0 | 7 |

WWS: western Washington State; NE: New England; Med: median; Y, yes; N, no; *different centers; ^@^ very high exposure vs no exposure; ^$^ ever exposure vs no exposure; ^#^Finland, Norway, Sweden, Denmark and Iceland; ^&^ France, Italy, Spain, United Kingdom, Netherlands, Greece and German.

**Supplementary Table 7 the information of studies investigating use of statins/cholesterol lowering agents for ALS involved this study**

| First author | Publish year | Population | Study design | Diagnostic category | Number of centers | Exposure | |  | Age (years) | | NOS |
| --- | --- | --- | --- | --- | --- | --- | --- | --- | --- | --- | --- |
|  |  |  |  |  |  | Cases (Y/N) | Controls (Y/N) |  | Cases | Controls |  |
| Sørensen, H. ^(20)^ | 2010 | Denmark | Case-control study | ICD-10 | 1 | 79/477 | 806/4754 |  | Mean:66.2 | Mean:66.2 | 7 |
| N A Sutedja ^(21)^ | 2011 | Netherlands | Case-control study | El Escorial criteria | 1 | 35/299 | 91/447 |  | Mean: 60 | Mean:59 | 6 |
| Freedman, D. M. ^(22)*^ | 2018 | USA | Case-control study | ICD-9 | 1 | 4870/5580 | 50738/53762 |  | NA | NA | 7 |
| Mariosa, D. ^(23)^ | 2020 | Sweden | Case-control study | ICD-7, ICD-8, ICD-9 and ICD-10 | 1 | 714/1761 | 3397/8978 |  | NA | NA | 7 |
| Kjetil Bjornevik ^(24)^ | 2020 | USA | Case-control study | ICD-9 | 5 | 38/236 | 55/492 |  | Mean:64.6 | Mean:64.6 | 6 |
| Kristin Diekmann ^(25)^ | 2020 | German | Case-control study | The El Escorial criteria | 1 | 35/165 | 26/171 |  | Mean:61.9 | Mean:59.4 | 7 |

*any statins;

**Supplementary Table 8 the information of studies investigating use of NSAIDs for ALS involved this study**

| First author | Publish year | Population | Study design | Diagnostic category | Number of centers | Exposure rate | |  | Age(Years) | | NOS |
| --- | --- | --- | --- | --- | --- | --- | --- | --- | --- | --- | --- |
|  |  |  |  |  |  | Cases (Y/N) | Controls (Y/N) |  | Cases | Controls |  |
| RITA A. POPAT ^(26)*^ | 2007 | USA | Case-control study | El Escorial criteria | 14 | 39/72 | 89/169 |  | Mean：62.6 | Mean：61.6 | 8 |
| RITA A. POPAT ^(26)&^ | 2007 | USA | Case-control study | El Escorial criteria | 14 | 41/70 | 90/168 |  | Mean：62.6 | Mean：61.6 | 8 |
| Lin, F. C ^(27)*^ | 2015 | Taiwanese | Case-control study | El Escorial criteria | 1 | 578/151 | 11457/3123 |  | NA | NA | 7 |
| Lin, F. C ^(27)&^ | 2015 | Taiwanese | Case-control study | El Escorial criteria | 1 | 111/618 | 2734/11846 |  | NA | NA | 7 |
| Kristin Diekmann ^(25)^ | 2020 | German | Case-control study | El Escorial criteria | 1 | 42/158 | 20/177 |  | Mean:61.9 | Mean：59.4 | 7 |

* Non-aspirin NSAID use, ever exposure vs. never exposure; ^&^Aspirin use

**Supplementary Table 9 the information of studies investigating diabetes mellitus for ALS involved this study**

| First author | Publish year | Population | Study design | Diagnostic category | Number of centers | Exposure | |  | Age (years) | | NOS |
| --- | --- | --- | --- | --- | --- | --- | --- | --- | --- | --- | --- |
|  |  |  |  |  |  | Cases (Y/N) | Controls(Y/N) |  | Cases | Controls |  |
| D. Mariosa ^(28)^ | 2014 | Sweden | Case-control study | ICD | 1 | 224/4884 | 1437/24103 |  | Mean:68.2 | Mean：68.2 | 8 |
| Meinie Seelen ^(29)^ | 2014 | Netherlands | Case-control study | The revised El Escorial | 1 | 49/673 | 184/2084 |  | Med:62.8 | Med:63.1 | 7 |
| Lin, F. C ^(27)^ | 2015 | Taiwanese | Case-control study | El Escorial criteria | 1 | 119/201 | 2430/12150 |  | NA | NA | 7 |
| Marianthi-Anna Kioumourtzoglou ^(30)^ | 2015 | Denmark | Case-control study | ICD | 1 | 55/3595 | 9239/355761 |  | NA | NA | 8 |
| Freedman, D. M. ^(22)^ | 2018 | USA | Case-control study | ICD-9 | 1 | 2665/7785 | 30393/74107 |  | NA | NA | 7 |
| Fabrizio D’Ovidio^(31)^ | 2018 | Euro-MOTOR | Case-control study | Revised El Escorial Criteria | 3 | 95/1439 | 204/2707 |  | NA | NA | 7 |
| Kjetil Bjornevik ^(24)^ | 2020 | USA | Case-control study | ICD-9 | 5 | 14/260 | 35/512 |  | Mean:64.6 | Mean:64.6 | 6 |
| Kristin Diekmann ^(25)^ | 2020 | German* | Case-control study | The El Escorial criteria | 1 | 18/182 | 16/181 |  | Mean:61.9 | Mean:59.4 | 7 |
| Gabriele nagel ^(32)^ | 2020 | German* | Case-control study | The revised El Escorial | 1 | 26/259 | 54/438 |  | Mean:65.4 | Mean:66.3 | 6 |

*different centers

**Supplementary Table 10 the information of studies investigating head trauma for ALS involved this study**

| First author | Publish year | Population | Study design | Diagnostic category | Number of centers | Exposure | |  | Age (years) | | NOS |
| --- | --- | --- | --- | --- | --- | --- | --- | --- | --- | --- | --- |
|  |  |  |  |  |  | Cases (Y/N) | Controls (Y/N) |  | Cases | Controls |  |
| Chen, H. ^(33)^ | 2007 | USA: NE# | Case-control study | El Escorial criteria | 2 | 24/85 | 42/213 |  | NA | NA | 7 |
| Binazzi, A. ^(16)^ | 2009 | Italy$ | Case-control study | El Escorial criteria | 4 | 16/61 | 23/162 |  | Mean: 65.0 | Mean: 57.5 | 7 |
| Furby, A. ^(3)^ | 2010 | France | Case-control study | El Escorial criteria | 1 | 19/89 | 14/108 |  | Med: 68 | Med:65 | 7 |
| Schmidt, S. ^(34)^ | 2010 | USA# | Case-control study | El Escorial criteria | 1 | 84/157 | 185/412 |  | Mean:62.4 | Mean: 61.7 | 7 |
| Peters, T. L. ^(35) &^ | 2013 | Swedish | Case-control study | ICD-9, ICD-10 | 1 | 63/3888 | 274/19632 |  | NA | NA | 7 |
| Seelen, M. ^(29)^ | 2014 | Netherlands | Case-control study | El Escorial criteria | 1 | 20/702 | 35/2233 |  | Med:62.8 | Med:63.1 | 7 |
| Seals, R. M. ^(36)^ | 2016 | Denmark | Case-control study | ICD-8, ICD-10 | 1 | 41/2459 | 3133/268220 |  | NA | NA | 6 |
| Pupillo, E. ^(37)^ | 2018 | EURALS consortium* | Case-control study | El Escorial criteria | 1 | 66/509 | 113/1035 |  | Mean:66 | 67 | 8 |
| Filippini, T. ^(38)^ | 2020 | Italy& | Case-control study | El Escorial Criteria | 4 | 19/76 | 13/122 |  | Mean:64.9 | Mean:66.5 | 6 |
| Andrew, A. S. ^(39)^ | 2021 | USA# | Case-control study | El Escorial criteria | 3 | 53/131 | 73/298 |  | NA | NA | 6 |
| Marie Beaudin^(40)^ | 2022 | Canada and France | Case-control study | Revised El Escorial criteria | 1 | 98/305 | 62/316 |  | NA | NA | 7 |
| G. X. Chen^(41)^ | 2022 | New Zealand | Case-control study | ICD-10 | 1 | 105/288 | 136/469 |  | NA | NA | 6 |

NE, New england; * five European countries (Italy, Ireland, France, United Kingdom, Serbia); ^#^ different cohorts; ^$^ different cohorts; ^&^ Repeated head injury >3 years before index date

**Supplementary Table 11 the information of studies investigating acute myocardial infarction/ischemic heart disease for ALS involved this study**

| First author | Publish year | Population | Study design | Diagnostic category | Number of centers | Exposure | |  | Age (years) | | NOS |
| --- | --- | --- | --- | --- | --- | --- | --- | --- | --- | --- | --- |
|  |  |  |  |  |  | Cases (Y/N) | Controls (Y/N) |  | Cases | Controls |  |
| Sørensen, H. T. ^(20)^ | 2010 | Denmark | Case-control study | ICD-10 | 1 | 44/512 | 422/5238 |  | Mean:66.2 | Mean:66.2 | 7 |
| Seelen, M. ^(29)^ | 2014 | Netherlands | Case-control study | El Escorial criteria | 1 | 31/691 | 108/2150 |  | Med:62.8 | Med:63.1 | 7 |
| Lin, F. C ^(27)#^ | 2015 | Taiwanese | Case-control study | El Escorial criteria | 1 | 120/609 | 2482/12098 |  | NA | NA | 7 |
| Freedman, D. M. ^(22)^ | 2018 | USA | Case-control study | ICD-9 | 1 | 284/10166 | 2963/101537 |  | Med:74 | Med:74 | 7 |
| Kristin Diekmann ^(25)^ | 2020 | German | Case-control study | El Escorial criteria | 1 | 14/186 | 7/190 |  | Mean:61.9 | Mean:59.4 | 7 |
| H. S. Abdel Magid^(42)^ | 2022 | USA | Case-control study | The El Escorial criteria | 1 | 41/3673 | 242/18328 |  | NA | NA | 7 |

^#^ Ischemic heart disease; other heart problem including coronary disease, cardiac arrhythmia, ceart failure were not analyzed

**Supplementary Table 12 the information of studies investigating stroke for ALS involved this study**

| First author | Publish year | Population | Study design | Diagnostic category | Number of centers | Exposure | |  | Age (Years) | | NOS |
| --- | --- | --- | --- | --- | --- | --- | --- | --- | --- | --- | --- |
|  |  |  |  |  |  | Cases (Y/N) | Controls(Y/N) |  | Cases | Controls |  |
| Sørensen, H. ^(20)^ | 2010 | Denmark | Case-control study | ICD-10 | 1 | 60/496 | 450/5110 |  | Mean:66.2 | Mean:66.2 | 7 |
| Seelen, M. ^(29)^ | 2014 | Netherlands | Case-control study | El Escorial criteria | 1 | 10/712 | 30/2238 |  | Med:62.8 | Med:63.1 | 6 |
| Lin, F. C ^(27)^ | 2015 | Taiwanese | Case-control study | El Escorial criteria | 1 | 157/572 | 2804/11776 |  | NA | NA | 7 |
| Freedman, D. M. ^(22)^ | 2018 | USA | Case-control study | ICD-9 | 1 | 341/10109 | 2666/101834 |  | Med:74 | Med:74 | 7 |
| F. D'Ovidio ^(31)^ | 2019 | Ireland, Italy, Netherland | Case-control study | El Escorial Criteria | 5 | 12/1489 | 28/2841 |  | NA | NA | 7 |
| Diekmann, K. ^(25)^ | 2020 | Germany | Case-control study | El Escorial criteria | 1 | 16/184 | 3/194 |  | Mean:61.9 | Mean:59.4 | 7 |

**Supplementary Table 13 the information of studies investigating high vitamin diet for ALS involved this study**

| First author | Publish year | Population | Study design | Diagnostic category | Number of centers | Exposure | |  | Age (Years) | | NOS |
| --- | --- | --- | --- | --- | --- | --- | --- | --- | --- | --- | --- |
|  |  |  |  |  |  | Cases (Y/N) | Controls (Y/N) |  | Case | controls |  |
| L. M. Nelson ^(43)^ | 2000 | USA | Case-control study | Clinic diagnose | 3 | 43/118 | 81/240 |  | NA | NA | 6 |
| K. Okamoto ^(44)^ | 2009 | Japan | Case-control study | El Escorial criteria | 2 | 36/117 | 78/228 |  | Mean:63.7 | Mean:63.4 | 7 |
| Tommaso Filippini ^(38)*^ | 2020 | Italy | Case-control study | El Escorial revised criteria | 2 | 17/78 | 29/106 |  | Mean:64.9 | Mean:66.5 | 6 |

*Vitamin supplements;

**Supplementary Table 14 the information of studies investigating coffee drinking for ALS involved this study**

| First author | Publish year | Population | Study design | Diagnostic category | Number of centers | Exposure | |  | Age (years) | | NOS |
| --- | --- | --- | --- | --- | --- | --- | --- | --- | --- | --- | --- |
|  |  |  |  |  |  | Cases (Y/N) | Controls (Y/N) |  | Case | Control |  |
| N. Morozova^(45)^ | 2008 | USA | Case-control study | El Escorial Criteria | 3 | 591/347 | 608387/ 348531 |  | NA | NA | 8 |
| Ettore Beghi ^(46)^ | 2011 | Italy | Case-control study | El Escorial criteria | 4 | 242/82 | 315/53 |  | NA | NA | 7 |
| Pupillo, E. ^(37)^ | 2018 | European | Case-control study | El Escorial criteria | 7 | 420/137 | 883/261 |  | Mean: 66 | Mean:67 | 8 |

***** Italy, Ireland, France, Serbia, England;

**Supplementary Table 15 the information of studies investigating smoking for ALS involved this study**

| First author | Publish year | Population | Study design | Diagnostic category | Number of centers | Exposure | |  | Age (Years) | | NOS |
| --- | --- | --- | --- | --- | --- | --- | --- | --- | --- | --- | --- |
|  |  |  |  |  |  | Cases (Y/N) | Controls(Y/N) |  | Cases | Controls |  |
| Fang, F ^(2)^ | 2009 | USA: NE* | Case-control study | El Escorial criteria | 2 | 77/32^ | 148/105^ | | Med: 60 | Med: 59 | 7 |
| Binazzi, A. ^(16)^ | 2009 | Italy: Rome | Case-control study | El Escorial criteria | 4 | 33/17/27 | 47/51/87 |  | Mean: 65.0 | Mean:67.5 | 7 |
| Okamoto, K. ^(44)^ | 2009 | Japan | Case-control study | El Escorial Criteria | 1 | 89/64^ | 166/140^ |  | Mean:63.7 | Mean:63.4 | 7 |
| Furby, A. ^(3)^ | 2010 | France | Case-control study | El Escorial criteria | 1 | 8/36/63 | 23/31/68 |  | Med: 68 | Med:65 | 7 |
| Schmidt, S. ^(34)^ | 2010 | US veterans | Case-control study | El Escorial criteria and ICD-9 | 1 | 39/121/81 | 102/281/214 |  | Mean: 62.4 | Mean:61.7 | 7 |
| Beghi, E. ^(46)^ | 2011 | Italy^#^ | Case-control study | El Escorial criteria | 4 | 174/203^ | 201/176^ |  | NA | NA | 7 |
| de Jong, S. W. ^(47)^ | 2012 | Netherlands | Case-control study | El Escorial Criteria | 1 | 187/412/337 | 216/830/553 |  | Mean::62.9 | Mean:62.7 | 7 |
| Das, K. ^(4)^ | 2012 | India | Case-control study | El Escorial criteria | 1 | 63/57^ | 100/140^ | | NA | NA | 6 |
| Jin, Y. ^(48)^ | 2014 | Korea | Case-control study | El Escorial Criteria | 1 | 31/41^ | 30/42^ |  | Mean:53.9 | Mean:53.4 | 8 |
| Malek, A. M. ^(49)^ | 2015 | USA: Pittsburgh | Case-control study | El Escorial Criteria | 3 | 39/27^ | 42/24^ |  | Mean:57.1 | Mean:56.4 | 8 |
| Andrew, A. S. ^(6)^ | 2017 | USA: NE* | Case-control study | El Escorial criteria | 2 | 25/131/131 | 10/91/110 |  | Med: 62 | Med: 61 | 7 |
| O'Reilly É, J. ^(50)^ | 2018 | USA | Case-control study | El Escorial criteria and ICD-9 | 5 | 18/128/123 | 36/240/264 |  | NA | NA | 6 |
| Rosenbohm, A. ^(51)^ | 2018 | Germany: Swabia | Case-control study | El Escorial Criteria | 1 | 132/154^ | 242/262^ |  | Mean: 65.7 | Mean:66.3 | 7 |
| Visser, A. E. ^(52)^ | 2018 | euro-MOTOR^$^ | Case-control study | El Escorial Criteria | 6 | 322/617/618 | 422/1351/1149 |  | NA | NA | 5 |
| Pupillo, E. ^(37)^ | 2018 | European^@^ | Case-control study | El Escorial criteria | 1 | 278/297^ | 535/613^ | | Mean:66 | Mean:67 | 8 |
| Diekmann, K. ^(25)^ | 2020 | Germany: Hannover | Case-control study | El Escorial criteria | 1 | 36/78/82 | 24/82/91 |  | Mean：61.9 | Mean:53.4 | 6 |
| Opie-Martin, S. ^(53)^ | 2020 | UK | Case-control study | El Escorial Criteria | 3 | 18/90/94 | 7/88/105 |  | Mean:63.1 | Mean:64.5 | 6 |
| Filippini, T. ^(38)^ | 2020 | Italy^&^ | Case-control study | El Escorial Criteria | 4 | 11/38/46 | 15/50/70 | | Mean: 64.9 | Mean:66.5 | 7 |
| Wismayer, M. F. ^(54)^ | 2021 | Malta | Case-control study | El Escorial criteria | 1 | 14/20^ | 10/35^ | | Med: 65.5 | Med:68 | 6 |
| Bjornevik, K. ^(24)^ | 2021 | USA: Michigan | Case-control study | El Escorial criteria and ICD-9 | 5 | 18/127/123 | 35/239/263 |  | Mean: 64.6 | Mean:64.6 | 7 |

NE: new England; * different centers; ^#^Lombardia, Piemonte and Valle D’Aosta, Puglia, and Liguria; ^&^ four provinces: Catania, Modena, Reggio Emilia, and Novara; ^$^Netherlands, Ireland and Italy; ^@^ five European countries: Italy, Ireland, France, United Kingdom, Serbia; ^ ever/never or yes/no; other, Current/ Former/never

**Supplementary Table 16 the information of studies investigating** **alcohol consumption for ALS involved this study**

| First author | Publish year | Population | Study design | Diagnostic category | Number of centers | Exposure | |  | Age (years) | | NOS |
| --- | --- | --- | --- | --- | --- | --- | --- | --- | --- | --- | --- |
|  |  |  |  |  |  | Cases (Y/N) | Controls (Y/N) |  | Case | Controls |  |
| Freya Kamel ^(55)^ | 2002 | USA: NE | Case-control study | El Escorial criteria | 2 | 31/61/17^@^ | 53/162/41^@^ |  | NA | NA | 6 |
| K. Okamoto ^(44)^ | 2009 | Japan | Case-control study | El Escorial criteria | 1 | 54/99^ | 96/210^ |  | Mean:63.7 | Mean63.4 | 8 |
| Ettore Beghi ^(46)^ | 2011 | Italy | Case-control study | El Escorial criteria | 4 | 166/211^ | 204/173^ |  | NA | NA | 7 |
| Sonja W. de Jong ^(47)^ | 2012 | Netherlands | Case-control study | El Escorial Criteria | 1 | 706/57/174^&^ | 1362/82/155^&^ |  | Mean:62.9 | Mean:62.7 | 6 |
| Youri Jin ^(48)^ | 2014 | Korea | Case-control study | El Escorial criteria | 1 | 44/28^ | 41/31^ |  | Mean:53.9 | Mean:53.4 | 7 |
| Pupillo, E. ^(37)^ | 2018 | European^#^ | Case-control study | El Escorial criteria | 1 | 241/334^ | 429/719^ |  | Mean:66 | Mean:67 | 8 |
| Anne E Visser ^(52)^ | 2018 | European* | Case-control study | El Escorial Criteria | 3 | 1082/125/350^&^ | 2295/124/503^&^ |  | NA | NA | 5 |
| Ling Lian ^(56)^ | 2019 | China | Case-control study | El Escorial criteria | 1 | 49/70^ | 68/171^ |  | NA | NA | 7 |
| Kjetil Bjornevik ^(24)^ | 2020 | USA | Case-control study | ICD-9 | 5 | 16/186/60^@^ | 35/383/109^@^ |  | Mean:64.6 | Mean:64.6 | 6 |
| Sarah Opie-Martin ^(53)^ | 2020 | UK | Case-control study | El Escorial criteria | 3 | 184/17^ | 176/24^ |  | Mean:63.1 | Mean:64.5 | 6 |
| Filippini, T. ^(38)^ | 2020 | Italy | Case-control study | El Escorial Criteria | 4 | 10/58^ | 21/113^ |  | Mean:64.9 | Mean:66.5 | 6 |
| Maia Farrudia Wismayer ^(54)$^ | 2021 | Malta | Case-control study | El Escorial criteria | 1 | 2/35 | 2/43 |  | Med: 65.5 | Med:68 | 6 |

^#^five European countries: Italy, Ireland, France, United Kingdom, Serbia; *Netherlands, Ireland and Italy; ^ ever/never; ^&^ current/former/never; NE, new England; ^@^high(>30gr/d)/ medium (1-30gr/d)/ never (0gr/d); ^$^ excessive/never or moderate

**Supplementary Table 17 the information of studies investigating military service for ALS involved this study**

| First author | Publish year | Population | Study design | Diagnostic category | Number of centers | Exposure | |  | Age (years) | | NOS |
| --- | --- | --- | --- | --- | --- | --- | --- | --- | --- | --- | --- |
|  |  |  |  |  |  | Cases(Y/N) | Controls(Y/N) |  | Cases | Controls |  |
| Weisskopf M. G.^(57)^ | 2005 | US | Cohort study | ICD-9 | 1 | 217/63 | 2587642/1100603 |  | NA | NA |  |
| Schmidt, S. ^(34)^ | 2010 | US veterans | Case-control study | El Escorial criteria | 1 | 99/142* | 200/397* |  | Mean:62.4 | Mean:61.7 | 7 |
| Tracy L Peters ^(8)^ | 2016 | Sweden | Case-control study | ICD-9 and ICD-10 | 1 | 31/4536 | 195/22207 |  | NA | NA | 6 |
| F. D’Ovidio ^(58)^ | 2017 | Italy | Case-control study | Clinic diagnose | 1 | 1/207 | 1249/ 283157 |  | NA | NA | 7 |
| Angeline S. Andrew ^(6)^ | 2017 | USA: NE | Case-control study | El Escorial criteria | 2 | 79/205 | 42/166 |  | Med: 62 | Med: 61 | 7 |
| Tommaso Filippini ^(9)&^ | 2020 | Italy^&^ | Case-control study | El Escorial criteria | 4 | 2/93 | 2/133 |  | Mean:64.9 | Mean:66.5 | 7 |
| Maia Farrudia Wismayer ^(54)^ | 2021 | Malta | Case-control study | El Escorial criteria | 1 | 1/37 | 0/45 |  | Med: 65.5 | Med:68 | 6 |

*army; ^&^ four provinces: Catania, Modena, Reggio Emilia, and Novara; NE, new England

**Supplementary Table 18 the information of studies investigating physical activity for ALS involved this study**

| First author | Publish year | Population | Study design | Diagnostic category | Number of centers | Exposure | |  | Age (years) | | NOS |
| --- | --- | --- | --- | --- | --- | --- | --- | --- | --- | --- | --- |
|  |  |  |  |  |  | Cases (Y/N) | Controls (Y/N) |  | Cases | Controls |  |
| Ettore Beghi ^(46)^ | 2011 | Italy | Case-control study | El Escorial criteria | 4 | 233/144* | 271/106* |  | NA | NA | 6 |
| Mark H B Huisman ^(59)^ | 2013 | Netherlands | Case-control study | El Escorial criteria | 1 | 103/510^#^ | 296/1815^#^ |  | Med:62 | Med:63 | 7 |
| Elisabetta Pupillo ^(60)^ | 2014 | European^&^ | Case-control study | El-Escorial criteria | 9 | 449/203* | 867/297* |  | NA | NA | 8 |
| Yu Yu ^(61)^ | 2014 | USA: Michigan | Case-control study | El Escorial criteria | 1 | 61/5* | 61/5* |  | NA | NA | 7 |
| Valentina Gallo ^(62)^ | 2016 | European^@^ | Cohort study | ICD-10 | 23 | 29/190^ | 86795/385305^ |  | NA | NA | 8 |
| Anne E Visser ^(52)^ | 2018 | European^￥^ | Case-control study | El Escorial Criteria | 3 | 76/1481^ | 130/2792^ |  | NA | NA | 5 |
| Gabriele nagel ^(32)^ | 2020 | Germany: Swabia | Case-control study | El Escorial criteria | 1 | 65/218^#^ | 64/434^#^ |  | Mean: 65.4 | Mean: 66.3 | 6 |
| Diekmann, K. ^(25)^ | 2020 | Germany: Hannover | Case-control study | El Escorial criteria | 1 | 156/40* | 132/63* |  | Mean: 61.9 | Mean: 59.4 | 7 |
| Maia Farrudia Wismayer ^(54)^ | 2021 | Malta | Case-control study | El Escorial criteria | 1 | 18/20^#^ | 5/40^#^ |  | Med: 65.5 | Med:68 | 6 |
| Bjornevik, K. ^(24)^ | 2021 | USA | Case-control study | El Escorial criteria and ICD-9 | 5 | 99/165^#^ | 198/331^#^ |  | Mean: 64.6 | Mean:64.6 | 7 |

^&^five European countries: Italy, Ireland, France, United Kingdom, Serbia; ^@^ten Western European countries: Norway, Sweden, Denmark, United Kingdom, Netherlands, Germany, France, Spain, Italy, and Greece; ^￥^Netherlands, Ireland, Italy; *ever/never; ^#^Vigorous physical activity; ^active/inactive, or active/ Moderately active and inactive

**Supplementary Table 19 the information of studies investigating high BMI for ALS involved this study**

| First author | Publish year | Population | Study design | Diagnostic category | Number of centers | Exposure rate | |  | Age | | NOS |
| --- | --- | --- | --- | --- | --- | --- | --- | --- | --- | --- | --- |
|  |  |  |  |  |  | Cases (n/N） | Controls（n/N） |  | Case | Controls |  |
| Lorene M. Nelson ^(43)^ | 2000 | USA: WWS | Case-control study | Clinic diagnose | 3 | 52/73/36^#^ | 127/155/41^#^ |  | NA | NA | 6 |
| N. Scarmeas ^(63)^ | 2002 | USA | Case-control study | El Escorial Criteria | 1 | 27/95/148^$^ | 29/46/64^$^ |  | NA | NA | 7 |
| N A Sutedja ^(21)^ | 2011 | Netherlands | Case-control study | El Escorial Criteria | 1 | 140/194* | 287/251* |  | Mean:60 | Mean:59 | 6 |
| Éilis J O'Reilly^(64)^ | 2013 | USA | Cohort study | ICD-9 | 11 | 86/307/276^$^ | 97341/264513/190601^$^ |  | NA | NA | 8 |
| Elisabetta Pupillo ^(60)^ | 2014 | European^￥^ | Case-control study | El Escorial criteria | 5 | 235/386^&^ | 627/506^&^ |  | NA | NA | 8 |
| Elisabetta Pupillo ^(65)^ | 2017 | Italy | Case-control study | El Escorial criteria | 3 | 20/63/129^$^ | 28/80/104^$^ |  | NA | NA | 6 |
| Gabriele nagel ^(32)^ | 2020 | German | Case-control study | El Escorial criteria | 1 | 55/63/176^ | 152/151/199^ |  | Mean: 65.4 | Mean:66.3 | 6 |

WWS, western Washington State; ^￥^five European countries: Italy, Ireland, France, United Kingdom, Serbia; ^#^BMI>26/21<BMI<26/BMI<21; ^$^ Obese (>30)/Overweight (25-30)/Normal or underweight (<25); * obese/non-obese; ^&^ Overweight (>25)/Normal or underweight (<25); ^ Obese (>28)/Overweight (25-28)/Normal or underweight (<25);

**Supplementary Table 20 the information of studies investigating electric shock for ALS involved this study**

| First author | Publish year | Population | Study design | Diagnostic category | Number of centers | Exposure rate | |  | Age | | NOS |
| --- | --- | --- | --- | --- | --- | --- | --- | --- | --- | --- | --- |
|  |  |  |  |  |  | Cases (n/N） | Controls（n/N） |  | Case | Controls |  |
| Ximena Vergara ^(12)^ | 2014 | USA | Case-control study | ICD-9 and ICD-10 | 1 | 69/91^1^ | 693/467 |  | NA | NA | 5 |
| Angeline S Andrew ^(6)^ | 2017 | USA: WWS | Case-control study | El Escorial Criteria | 2 | 19/258 | 12/189 |  | Med: 62 | Med: 61 | 7 |
| Koeman, T. ^(7)^ | 2017 | Netherlands | Cohort study | ICD-9 and ICD-10 | 1 | 18/98/20^2^ | 524/3201/441 |  | NA | NA | 7 |
| Susan Peters ^(15)^ | 2019 | Ireland, Italy, Netherlands | Case-control study | ICD-9 and ICD-10 | 3 | 270/261/792^3^ | 453/452/1799 |  | Mean:64.4 | Mean:64.0 | 7 |
| Tommaso Filippini ^(38)^ | 2020 | Italy | Case-control study | El Escoria lrevised criteria | 2 | 7/88 | 5/130 |  | Mean: 64.9 | Mean:66.5 | 7 |

WWS: western Washington State；1. High/Not high；2. Ever high/Background/Only ever low；3. Ever high/Only medium/Background

**Supplementary Table 21 the information of studies investigating living in urban for ALS involved this study**

| First author | Publish year | Population | Study design | Diagnostic category | Number of centers | Exposure rate | |  | Age | | NOS |
| --- | --- | --- | --- | --- | --- | --- | --- | --- | --- | --- | --- |
|  |  |  |  |  |  | Cases (n/N） | Controls（n/N） |  | Case | Controls |  |
| Julia M. Morahan ^(66)^ | 2006 | Australia | Case-control study | El Escorial criteria | 1 | 74/105 | 91/88 |  | NA | NA | 8 |
| Kamalesh Das ^(4)^ | 2012 | India | Case-control study | El Escorial criteria | 2 | 12/98 | 47/193 |  | NA | NA | 7 |
| Feng-Cheng Lin ^(27)^ | 2015 | Taiwan | Cohort study | El Escorial criteria | 1 | 575/154 | 11500/3038 |  | NA | NA | 7 |
| Sonja Korner ^(67)^ | 2019 | German | Case-control study | El Escorial criteria | 3 | 50/61 | 59/34 |  | NA | NA | 8 |
| Maia Farrugia Wismayer ^(54)^ | 2021 | Malta | Case-control study | El Escorial criteria | 2 | 33/5 | 40/5 |  | Med: 65.5 | Med:68 | 6 |

**Supplementary Table 22 the information of studies investigating hypertension for ALS involved this study**

| First author | Publish year | Population | Study design | Diagnostic category | Number of centers | Exposure rate | |  | Age | | NOS |
| --- | --- | --- | --- | --- | --- | --- | --- | --- | --- | --- | --- |
|  |  |  |  |  |  | Cases (n/N） | Controls（n/N） |  | Case | Controls |  |
| Meinie Seelen ^(29)^ | 2014 | Netherlands | Case-control study | El Escorial criteria | 1 | 233/489 | 784/1484 |  | Med:62.8 | Med:63.1 | 6 |
| Feng-Cheng Lin ^(27)^ | 2015 | Taiwan | Cohort study | El Escorial criteria | 1 | 254/475 | 113910/691718 |  | NA | NA | 7 |
| Fabrizio D’Ovidio ^(31)^ | 2019 | Ireland, Italy, Netherland | Case-control study | El Escorial Criteria | 5 | 105/143 | 129/156 |  | NA | NA | 7 |
| D. Michal Freedman ^(22)^ | 2018 | USA | Case-control study | ICD-9 | 3 | 7122/3328 | 70114/34386 |  | Med:74 | Med:74 | 7 |
| Kristin Diekmann^(25)^ | 2020 | Germany: Hannover | Case-control study | El Escorial criteria | 1 | 98/102 | 89/108 |  | Mean: 61.9 | Mean: 59.4 | 7 |

**Supplementary Table 23 the information of studies investigating kidney disease for ALS involved this study**

| First author | Publish year | Population | Study design | Diagnostic category | Number of centers | Exposure rate | |  | Age | | NOS |
| --- | --- | --- | --- | --- | --- | --- | --- | --- | --- | --- | --- |
|  |  |  |  |  |  | Cases (n/N） | Controls（n/N） |  | Case | Controls |  |
| Henrik Toft Sørensen ^(20)^ | 2010 | Denmark | Case-control study | ICD-10 | 1 | 3/553 | 61/5499 |  | Mean:66.2 | Mean:66.2 | 7 |
| Feng-Cheng Lin ^(27)^ | 2015 | Taiwan | Cohort study | El Escorial criteria | 1 | 8/721 | 176/14404 |  | NA | NA | 7 |
| D. Michal Freedman ^(22)^ | 2018 | USA | Case-control study | ICD-9 | 1 | 734/9716 | 8589/95911 |  | Med:74 | Med:74 | 7 |

**Supplementary Table 24 the information of studies investigating antidiabetics for ALS involved this study**

| First author | Publish year | Population | Study design | Diagnostic category | Number of centers | Exposure rate | |  | Age | | NOS |
| --- | --- | --- | --- | --- | --- | --- | --- | --- | --- | --- | --- |
|  |  |  |  |  |  | Cases (n/N） | Controls（n/N） |  | Case | Controls |  |
| N A Sutedja ^(21)^ | 2011 | Netherlands | Case-control study | El Escorial criteria | 1 | 15/319 | 29/309 |  | Mean: 60 | Mean:59 | 6 |
| D. Mariosa ^(23)^ | 2020 | Sweden | Case-control study | ICD-7, ICD-8, ICD-9 and ICD-10 | 1 | 241/473 | 1705/1692 |  | NA | NA | 7 |
| Kristin Diekmann ^(25)^ | 2020 | Germany: Hannover | Case-control study | El Escorial criteria | 1 | 16/184 | 16/181 |  | Mean: 61.9 | Mean: 59.4 | 7 |

**Supplementary Table 25 the information of studies investigating *SOD1* gene mutation involved this study**

| First author | Publish year | Population | Study design | Diagnostic category | Number of centers | Detection methods | Mutation rate | | NOS |
| --- | --- | --- | --- | --- | --- | --- | --- | --- | --- |
|  |  |  |  |  |  |  | FALS(n/N) | SALS(n/N) |  |
| Cheryl T Jones^(68)^ | 1994 | The Scotland | Cohort study | Clinic diagnose | 1 | PCR | 5/10 | 4/57 | 6 |
| A. Pramatarova^(69)^ | 1994 | Canada | Cohort study | Clinic diagnose | 1 | PCR，SSCP | 15/114 | / | 6 |
| M. E. Cudkowicz^(70)^ | 1996 | USA | Case-control | Clinic diagnose | 1 | Sanger sequencing | 194/340 | / | 6 |
| P.M. Andersen^(71)^ | 1997 | Denmark, Finland, Norway and Sweden | Case-control | Clinic diagnose | 1 | PCR,SSCP | 27/72 | 14/355 | 7 |
| Mandy Jackson^(72)^ | 1997 | UK | Cohort study | El Escorial criteria | 1 | SSCP | / | 4/155 | 7 |
| Y. Boukaftane^(73)^ | 1998 | Canada, France | Case-control | El Escorial criteria | 2 | SSCP | 10/70 | / | 8 |
| Tania Aguirre^(74)^ | 1999 | Belgium | Case-control | El Escorial criteria | 1 | SSCP | 7/11 | 1/69 | 7 |
| V. I. Skvortsova^(75)^ | 2000 | Russia | Case-control | El Escorial criteria | 1 | SSCP，PCR | / | 2/20 | 5 |
| Barry A Chioza^(76)^ | 2001 | UK | Case-control | El Escorial criteria | 1 | SSCP | 0/31 | 8/161 | 7 |
| Cinzia Gellera^(77)^ | 2001 | Italy | Cohort study | Clinic diagnose | 1 | NA | 7/36 | 3/48 | 5 |
| A. García-Redondo^(78)^ | 2002 | Spain | Cohort study | El Escorial criteria | 1 | PCR、SSCP | 2/11 | 1/87 | 7 |
| Takako Sato^(79)^ | 2003 | Japan | Cohort study | El Escorial criteria | 1 | PCR | 10/33 | / | 6 |
| Stefania Battistini^(80)^ | 2005 | Italy | Case-control | The revised El Escorial criteria | 5 | SSCP | 7/39 | 14/225 | 8 |
| L. Corrado^(81)^ | 2006 | Italy | Case-control | The revised El Escorial criteria | 1 | PCR | 0/4 | 3/66 | 6 |
| Josep Gamez^(82)^ | 2006 | Italy | Cohort study | Clinic diagnose | 1 | PCR | 5/30 | 2/94 | 6 |
| Andrew Eis en^(83)^ | 2007 | British, USA, Canada | Case-control | Clinic diagnose | 1 | SSCP | 10/47 | 3/159 | 7 |
| Natasha Luquin^(84)^ | 2008 | Australia | Case-control | The revised El Escorial criteria | 1 | PCR | / | 21/23 | 5 |
| Michael A van Es  ^(85)^ | 2009 | The Netherlands | Cohort study | The revised El Escorial criteria | 1 | PCR | 1/55 | 2/451 | 7 |
| Malessa Rabe^(86)^ | 2010 | Germany, Austria，Switzerland | Cohort study | Clinic diagnose | 3 | PCR | 28/217 | / | 8 |
| Hannu Laaksovirta^(87)^ | 2010 | Finland | Case-control | El Escorial criteria | 1 | GWAS | 27/93 | 13/312 | 7 |
| Ste´phanie Millecamps^(88)^ | 2010 | France | Case-control | El Escorial criteria | 1 | PCR | 20/162 | / | 7 |
| C. Akimoto^(89)^ | 2011 | Japan | Case-control | The revised El Escorial criteria | 1 | HRM | 4/10 | 7/439 | 6 |
| Jeffrey A. Brown^(90)^ | 2011 | USA | Case-control | Clinic diagnose | 1 | HRM, Sanger sequencing | / | 92/1220 | 6 |
| Min-Jung Kwon^(91)^ | 2011 | Korea | Cohort study | The revised El Escorial criteria | 1 | PCR | 7/9 | 3/29 | 6 |
| Piaceri I^(92)^ | 2011 | Italy | Cohort study | El Escorial criteria | 1 | PCR | 0/6 | 1/61 | 7 |
| Adriano Chiò^(93)^ | 2012 | Italy | Case-control | The revised El Escorial criteria | 1 | WES | 4/11 | 6/429 | 7 |
| Serena Lattante^(94)^ | 2012 | Italy | Case-control | El Escorial criteria | 1 | PCR | 7/48 | 10/480 | 8 |
| Marka van Blitterswijk^(95)^ | 2012 | the Netherlands | Case-control | El Escorial criteria | 1 | PCR | 1/97 | 2/451 | 6 |
| Elena Pasini^(96)^ | 2012 | Italy | Case-control | The revised El Escorial criteria | 1 | WES | 5/23 | 0/2 | 5 |
| Kevin P kenna ^(97)^ | 2013 | Ireland | Case-control | The revised El Escorial criteria | 1 | WES | / | 17/1447 | 8 |
| Lee, Y. C.^(98)^ | 2013 | China | Case-control | El Escorial criteria | 1 | PCR | 8/30 | 4/131 | 7 |
| Lysogorskaia, E.^(99)^ | 2013 | Rusia | Case-control | El Escorial criteria | 1 | PCR | 4/8 | 6/199 | 6 |
| Julien Couthouis  ^(100)^ | 2014 | USA | Case-control | Clinic diagnose | 1 | Sanger sequencing | / | 134/276 | 6 |
| Bing-Wen Soong^(101)^ | 2014 | China | Cohort study | The revised El Escorial criteria | 1 | PCR | 8/30 | 4/131 | 7 |
| Aslıhan Özoguz^(102)^ | 2014 | Turkey | Cohort study | El Escorial criteria | 1 | PCR，sanger sequencing | 12/82 | / | 6 |
| S Niemann^(103)^ | 2015 | Germany， Austria and Switzerland | Case-control | Clinic diagnose | 1 | PCR | 9/75 | / | 7 |
| Zhang-Yu Zou^(104)^ | 2015 | China | Case-control | Clinic diagnose | 1 | PCR | 6/20 | 4/324 | 6 |
| Janet Cady^(105)^ | 2015 | USA | Case-control | El Escorial criteria | 1 | Sanger sequencing | 4/84 | 4/349 | 7 |
| Gerson Chadi^(106)^ | 2016 | Brazil | Case-control | The revised El Escorial criteria | 1 | PCR | 3/39 | 0/189 | 5 |
| Emily P. McCann^(107)^ | 2017 | Australia | Cohort study | El Escorial criteria | 1 | PCR | 29/267 | 97/746 | 7 |
| Morgan, S.^(108)^ | 2017 | Britain | Case-control | El Escorial criteria | 1 | NGS | / | 8/1126 | 8 |
| Müller, K.^(109)^ | 2018 | Germany | Cohort study | El Escorial criteria | 9 | fragment length analysis, PCR, Southern blotting, Sanger sequencing and WES | 37/301 | / | 8 |
| Hang Zhang^(110)^ | 2018 | China | Case-control | The revised El Escorial criteria | 1 | Sanger sequencing | / | 7/311 | 7 |
| Zhi-Jun Liu^(111)^ | 2019 | China | Cohort study | El Escorial criteria | 1 | NGS | / | 12/24 | 6 |
| Maurizio Grassano^(112)^ | 2020 | Germany | Case-control | The revised El Escorial criteria | 1 | PCR | 1/107 | 16/852 | 7 |
| W. Chen^(113)^ | 2020 | China | Case-control | El Escorial criteria | 1 | Sanger sequencing | 1/15 | 4/253 | 6 |
| Yong-Ping Chen^(114)^ | 2021 | China | Case-control | El Escorial criteria | 1 | WES, PCR | 14/64 | 29/1523 | 8 |
| Suzanna Edgar^(115)^ | 2021 | Malaya | Case-control | The revised El Escorial criteria | 1 | Sanger sequencing | 1/3 | 2/98 | 7 |
| W. Liu^(116)^ | 2021 | China | Case-control | The revised El Escorial criteria | 1 | PCR | 19/242 | / | 7 |
| Zhen Liu^(117)^ | 2021 | China | Case-control | The revised El Escorial criteria | 1 | PCR | 8/62 | 19/691 | 7 |

PCR：polymerase chain reaction；SSCP：single strand conformation polymorphism；WES:Whole exome sequencing；HRM:High-resolution melting curve；GWAS：genome wide association study；NGS:Next-generation sequencin

**Supplementary Table 26 the information of studies investigating *SQSTM1* gene mutation involved this study**

| First author | Publish year | Population | Study design | Diagnostic  category | Number of centers | Detection methods | Mutation rate | | NOS |
| --- | --- | --- | --- | --- | --- | --- | --- | --- | --- |
|  |  |  |  |  |  |  | FALS(n/N) | SALS(n/N) |  |
| Faisal Fecto^(118)^ | 2011 | USA | Case-control | The revised El Escorial criteria | 1 | PCR | 6/340 | 9/206 | 7 |
| Makito Hirano^(119)^ | 2013 | Japan | Case-control | The revised El Escorial criteria | 1 | PCR | 0/7 | 2/54 | 5 |
| Elisa Teyssou^(120)^ | 2013 | France | Case-control | Clinic diagnose | 1 | PCR | 1/90 | 3/74 | 8 |
| Lee, Y. C.^(98)^ | 2013 | China | Case-control | El Escorial criteria | 1 | PCR | 0/30 | 0/131 | 7 |
| Yi Yang ^(121)^ | 2015 | China | Case-control | The revised El Escorial criteria | 1 | PCR | 0/35 | 6/436 | 7 |
| Chun Tak Kwok^(122)^ | 2015 | UK | Case-control | Clinic diagnose | 1 | PCR | 5/61 | / | 6 |
| Morgan, S.^(108)^ | 2017 | Britain | Case-control | El Escorial criteria | 1 | NGS | / | 3/1126 | 8 |
| Müller, K.^(109)^ | 2018 | Germany | case series | El Escorial criteria | 9 | fragment length analysis, PCR, Southern blotting, Sanger sequencing and WES | 1/301 | / | 8 |
| Oriol Dols-Icardo ^(123)^ | 2018 | Spain | Cohort study | El Escorial criteria | 1 | WES | 0/10 | 3/44 | 6 |
| Rüstem Yilmaz^(124)^ | 2020 | Germany and Sweden | Cohort study | Clinic diagnose | 2 | WES | 12/486 | / | 6 |
| Priyam Narain^(125)^ | 2018 | India | Cohort study | The revised El Escorial criteria | 1 | NGS | 0/5 | 2/149 | 6 |
| Viviana Pensato^(126)^ | 2020 | Italy | Cohort study | Clinic diagnose | 1 | NGS | / | 2/213 | 7 |
| Emily P McCann^(127)^ | 2020 | Australia | Cohort study | El Escorial criteria | 1 | WGS | / | 41/616 | 7 |
| YongPing Chen(114) | 2021 | China | Case-control | The revised El Escorial criteria | 1 | PCR | 1/64 | 5/1523 | 7 |

WGS:whole-genome shotgun

**Supplementary Table 27 the information of studies investigating *TARDBP* gene mutation involved this study**

| First author | Publish year | Population | Study design | Diagnostic  category | Number of centers | Detection methods | Mutation rate | | NOS |
| --- | --- | --- | --- | --- | --- | --- | --- | --- | --- |
|  |  |  |  |  |  |  | FALS(n/N) | SALS(n/N) |  |
| Nicola J. Rutherford^(128)^ | 2008 | USA | Case-control | Clinic diagnose | 1 | PCR | 3/92 | 0/24 | 6 |
| Jemeen Sreedharan^(129)^ | 2008 | UK，Australia | Cohort study | El Escorial criteria | 2 | GWAS | 5/154 | / | 6 |
| Edor Kabashi ^(130)^ | 2008 | France，Canada | Cohort study | Clinic diagnose | 2 | WES | 4/80 | 6/120 | 8 |
| Rita J. Guerreiro^(131)^ | 2008 | USA | Case-control | Clinic diagnose | 1 | PCR | / | 5/279 | 7 |
| Akio Yokoseki^(132)^ | 2008 | Japan | Cohort study | Clinic diagnose | 1 | PCR | / | 0/112 | 6 |
| R. Del Bo^(133)^ | 2009 | Italy，Greece，Belgium | Case-control | the El Escorial criteria | 3 | PCR | 0/16 | 14/298 | 8 |
| Masaki Kamada^(134)^ | 2009 | Japan | Case-control | Clinic diagnose | 1 | PCR | 1/30 | 0/220 | 6 |
| Nicola Ticozzi^(135)^ | 2009 | USA | Case-control | The revised El Escorial criteria | 2 | PCR | 13/209 | 1/188 | 7 |
| Lucia Corrado^(136)^ | 2009 | Italy | Case-control | The revised El Escorial criteria | 1 | PCR | 6/135 | 12/531 | 6 |
| F. L. Conforti ^(137)^ | 2010 | Italy | Case-control | the El Escorial criteria | 1 | WES | 3/12 | 3/298 | 7 |
| Hui-Ling Xiong^(138)^ | 2010 | China | Case-control | the Airlie House criteria | 1 | PCR | 1/5 | 2/71 | 5 |
| Orru S^(139)^ | 2011 | Italy | Cohort study | The revised El Escorial criteria | 1 | PCR | 24/30 | 6/67 | 5 |
| Rui Huang^(140)^ | 2012 | China | Case-control | The revised El Escorial criteria | 1 | PCR | / | 6/165 | 6 |
| Aritoshi Iida^(141)^ | 2012 | Japan | Cohort study | The revised El Escorial criteria | 1 | PCR | 0/21 | 3/700 | 7 |
| D. Czell^(142)^ | 2012 | Switzerland | prospective study | The revised El Escorial criteria | 1 | WES | 4/43 | 0/182 | 7 |
| Cheng-hui Ye^(143)^ | 2013 | China | Case-control | The revised El Escorial criteria | 1 | PCR | / | 0/207 | 7 |
| Lee, Y. C.^(98)^ | 2013 | China | Case-control | El Escorial criteria | 1 | PCR | 7/30 | 0/131 | 7 |
| H Daoud^(144)^ | 2014 | France | Case-control | El Escorial criteria | 1 | PCR | / | 9/285 | 6 |
| Morgan, S.^(108)^ | 2017 | Britain | Case-control | El Escorial criteria | 1 | NGS | / | 7/1126 | 8 |
| Guo-rong Xu^(145)^ | 2018 | China | Case-control | The revised El Escorial criteria | 1 | Sanger sequencing | 5/7 | 5/215 | 7 |
| Müller, K.^(109)^ | 2018 | Germany | case series | El Escorial criteria | 9 | fragment length analysis, PCR, Southern blotting, Sanger sequencing and WES | 12/301 | / | 8 |
| Marzieh Khani^(146)^ | 2019 | Iranian | Cohort study | El Escorial criteria | 1 | PCR | 1/19 | 0/107 | 7 |
| Feng Wang ^(147)^ | 2020 | China | Cohort study | El Escorial criteria | 1 | high-resolution melting analysis | / | 3/384 | 7 |
| M. Gromicho^(148)^ | 2020 | Portugal | Cohort study | The revised El Escorial criteria | 1 | Sanger sequencing | 2/19 | 0/246 | 6 |
| Feng Feng^(149)^ | 2021 | China | Cohort study | The revised El Escorial criteria | 1 | PCR | / | 3/384 | 6 |
| YongPing Chen^(114)^ | 2021 | China | Case-control | The revised El Escorial criteria | 1 | PCR | 2/64 | 13/1523 | 7 |
| W. Liu^(116)^ | 2021 | China | Case-control | The revised El Escorial criteria | 1 | PCR | 4/242 | / | 7 |
| J. P. Nunes Gonçalves^(150)^ | 2021 | Brazil | Cohort study | El Escorial criteria | 3 | WES | 4/107 | / | 6 |
| J. Li^(151)^ | 2022 | China | Case-control | The revised El Escorial criteria | 1 | WES | 1/54 | 3/1240 | 7 |

**Supplementary Table 28 the information of studies investigating *ATNX2* gene mutation involved this study**

| First author | Publish year | Population | Study design | Diagnostic  category | Number of centers | Detection methods | Mutation rate | | NOS |
| --- | --- | --- | --- | --- | --- | --- | --- | --- | --- |
|  |  |  |  |  |  |  | FALS(n/N) | SALS(n/N) |  |
| Corrado, L^.(152)^ | 2011 | Italy | Case-control | The revised El Escorial criteria | 1 | PCR | 0/13 | 7/219 | 7 |
| Daoud, H.^(153)^ | 2011 | France and Quebec, Canada | Case-control | El Escorial criteria | 2 | PCR | 2/95 | 9/461 | 8 |
| Chen, Y.^(154)^ | 2011 | China | Case-control | El Escorial criteria | 1 | PCR | / | 15/345 | 7 |
| Ross, O. A.^(155)^ | 2011 | Britain,USA | Case-control | El Escorial criteria | 4 | PCR | / | 8/532 | 5 |
| Lahut, S.^(156)^ | 2012 | Turkish | Case-control | El Escorial criteria | 1 | PCR | 0/78 | 4/158 | 7 |
| Conforti, F. L.^(157)^ | 2012 | Italy | Case-control | The revised El Escorial criteria | 1 | PCR,SSCP | 13/13 | 11/405 | 6 |
| Tim Van Langenhove^(158)^ | 2012 | Belgian | Case-control | Clinic diagnose | 1 | PCR | 0/18 | 1/54 | 7 |
| Liu, X.^(159)^ | 2013 | China | Case-control | The revised El Escorial criteria | 1 | PCR | 0/6 | 14/1061 | 7 |
| Lee, Y. C.^(98)^ | 2013 | China | Case-control | El Escorial criteria | 1 | PCR | 0/30 | 2/131 | 7 |
| Serena Lattante^(160)^ | 2014 | France | Case-control | El Escorial criteria | 1 | PCR | 11/409 | 11/735 | 6 |
| Lu, H. P.^(161)^ | 2015 | China | Case-control | The revised El Escorial criteria | 1 | PCR | 2/15 | 17/379 | 6 |
| Borghero, G.^(162)^ | 2015 | Sardinian ancestry | Case-control | The revised El Escorial criteria | 1 | PCR | 0/1 | 0/4 | 5 |
| Chiò, A.^(163)^ | 2016 | Italian and Sardinian | Case-control | El Escorial criteria | 18 | PCR | / | 66/1639 | 7 |
| Kim, Y. E.^(164)^ | 2018 | Korea | Case-control | The revised El Escorial criteria | 1 | PCR | / | 7/464 | 6 |
| Tavares de Andrade, H. M.^(165)^ | 2018 | Brazilian | Case-control | El Escorial criteria | 6 | PCR | 7/87 | 22/372 | 7 |
| Zhen Liu^(166)^ | 2021 | China | Case-control | The revised El Escorial criteria | 1 | WES | / | 7/691 | 7 |
| Borg, R.^(167)^ | 2021 | Malta | Case-control | El Escorial criteria | 1 | WGS | 0/3 | 1/21 | 6 |

**Supplementary Table 29 the information of studies investigating *C9orf72* gene mutation involved this study**

| First author | Publish year | Population | Study design | Diagnostic  category | Number of centers | Detection methods | Mutation rate | | NOS |
| --- | --- | --- | --- | --- | --- | --- | --- | --- | --- |
|  |  |  |  |  |  |  | FALS(n/N) | SALS(n/N) |  |
| Chiò, A.^(168)^ | 2012 | Italy，German | Case-control | El Escorial criteria | 2 | PCR | 66/182 | / | 5 |
| Majounie, E^.(169)^ | 2012 | Finland,Dutch, English | Case series | El Escorial criteria | 17 | PCR | 244/3860 | 221/588 | 8 |
| Ishiura, H^.(170)^ | 2012 | Japan | Case series | El Escorial criteria | 2 | PCR,SSCP | 1/7 | 1/47 | 5 |
| Ogaki, K^.(171)^ | 2012 | Japan | Case series | The revised El Escorial criteria | 1 | PCR | 0/11 | 2/552 | 7 |
| Ratti, A.^(172)^ | 2012 | Italy | Cohort study | The revised El Escorial criteria | 6 | PCR,SSCP | 62/259 | 66/1275 | 8 |
| Sabatelli, M.^(173)^ | 2012 | Italy | Case series | The revised El Escorial criteria | 3 | PCR | 69/1523 | / | 7 |
| Stewart, H^.(174)^ | 2012 | Canada | Case series | The revised El Escorial criteria | 2 | PCR,SSCP | 17/62 | 6/169 | 7 |
| Tsai, C. P.^(175)^ | 2012 | China | Case-control | The revised El Escorial criteria | 1 | PCR | 4/22 | 2/102 | 5 |
| van Rheenen, W^.(176)^ | 2012 | Dutch | Case-control | El Escorial criteria | 3 | PCR | 33/78 | 87/1422 | 8 |
| Xi, Z^.(177)^ | 2012 | European | Case-control | established clinical criteria | 4 | PCR | 18/47 | 18/342 | 8 |
| Marka van Blitterswijk^(95)^ | 2012 | the Netherlands | Case-control | El Escorial criteria | 1 | PCR | 5/97 | 3/451 | 6 |
| Serena Lattante^(94)^ | 2012 | Italy | Case-control | El Escorial criteria | 1 | PCR | 0/48 | 12/480 | 8 |
| Debray, S.^(178)^ | 2013 | Belgium | Case-control | El Escorial criteria or Awaji criteria | 1 | PCR，TP-PCR | 32/62 | 45/471 | 8 |
| Jang, J. H.^(179)^ | 2013 | Korea | Case series | El Escorial criteria | 1 | PCR | 0/246 | 0/8 | 6 |
| Smith, B. N.^(180)^ | 2013 | Northern European Caucasian | Case series | The revised El Escorial criteria | 1 | PCR | 89/229 | 75/979 | 8 |
| Lee, Y. C.^(98)^ | 2013 | China | Case-control | El Escorial criteria | 1 | PCR | 5/30 | 2/131 | 7 |
| Lysogorskaia, E.^(99)^ | 2013 | Rusia | Case-control | El Escorial criteria | 1 | PCR | 0/8 | 5/199 | 6 |
| Aslıhan Özoguz^(102)^ | 2014 | Turkey | Cohort study | El Escorial criteria | 1 | PCR，sanger sequencing | 17/82 | 16/361 | 6 |
| Jiao, B.^(181)^ | 2014 | China | Case-control | El Escorial criteria | 1 | PCR | 1/10 | 0/100 | 7 |
| He, J.^(182)^ | 2015 | China | Case-control | The revised El Escorial criteria | 1 | PCR，SSCP | / | 3/1092 | 7 |
| Van Es, M. A.^(183)^ | 2015 | the Netherlands | Case-control | El Escorial criteria | 1 | PCR | 13/755 | / | 7 |
| Vrabec, K^.(184)^ | 2015 | Slovenia | Case series | El Escorial criteria | 1 | PCR，SSCP | / | 5/85 | 6 |
| Zhang-Yu Zou^(104)^ | 2015 | China | Case-control | Clinic diagnose | 1 | PCR | 1/20 | 1/324 | 6 |
| Janet Cady^(105)^ | 2015 | USA | Case-control | El Escorial criteria | 1 | Sanger sequencing | 11/84 | 21/698 | 7 |
| Itzcovich, T^.(185)^ | 2016 | Argentina | Case-control | Established criteria | 2 | PCR | 1/3 | 1/47 | 7 |
| Gerson Chadi^(106)^ | 2016 | Brazil | Case-control | The revised El Escorial criteria | 1 | PCR | 5/39 | 5/189 | 5 |
| Gibson, S. B.^(186)^ | 2017 | USA(European) | Case-control | El Escorial criteria | 1 | PCR | / | 5/87 | 6 |
| Narain, P^.(187)^ | 2017 | India | Case-control | El Escorial criteria | 1 | PCR | 1/4 | 3/127 | 7 |
| Vats, A.^(188)^ | 2017 | India | Case-control | El Escorial criteria | 1 | PCR | 0/2 | 0/73 | 7 |
| Emily P. McCann^(107)^ | 2017 | Australia | Cohort study | El Escorial criteria | 1 | PCR | 86/267 | 121/746 | 7 |
| C. Kartanou^(189)^ | 2018 | Greek | Case-control | El Escorial criteria | 2 | PCR，sanger sequencing | 14/25 | 19/306 | 6 |
| Müller, K.^(109)^ | 2018 | Germany | case series | El Escorial criteria | 9 | fragment length analysis, PCR, Southern blotting, Sanger sequencing and WES | 75/301 | / | 8 |
| Cintra, V. P.^(190)^ | 2018 | Brazil | Case-control | El Escorial criteria | 7 | PCR | 8/68 | 11/309 | 7 |
| Tripolszki, K.^(191)^ | 2019 | Hungary | Case series | The revised El Escorial criteria | 1 | PCR,SSCP | 2/10 | 8/97 | 6 |
| Trojsi, F^.(192)^ | 2019 | Italy | Case series | The revised El Escorial criteria | 13 | PCR | / | 84/1417 | 8 |
| Roggenbuck, J^.(193)^ | 2020 | USA | Cohort study | El Escorial criteria | 1 | PCR | 2/142 | 3/25 | 7 |
| Sokratous, M.^(194)^ | 2020 | Greek | Case series | El Escorial criteria | 2 | PCR-SSCP | 9/178 | / | 7 |
| W. Chen^(113)^ | 2020 | China | Case-control | El Escorial criteria | 1 | Sanger sequencing | 0/15 | 4/253 | 6 |
| W. Liu^(116)^ | 2021 | China | Case-control | The revised El Escorial criteria | 1 | PCR | 1/242 | / | 7 |
| Yong-Ping Chen^(114)^ | 2021 | China | Case-control | El Escorial criteria | 1 | WES,PCR | 0/64 | 20/1523 | 8 |
| Suzanna Edgar^(115)^ | 2021 | Malaya | Case-control | The revised El Escorial criteria | 1 | Sanger sequencing | 1/3 | 2/98 | 7 |

TP-PCR：Triplet repeat primed- Polymerase chain reaction

**Supplementary Table 30 the information of studies investigating *FUS* gene mutation involved this study**

| First author | Publish year | Population | Study design | Diagnostic  category | Number of centers | Detection methods | Mutation rate | | NOS |
| --- | --- | --- | --- | --- | --- | --- | --- | --- | --- |
|  |  |  |  |  |  |  | FALS(n/N) | SALS(n/N) |  |
| Belzil, V. V.^(195)^ | 2009 | French and French Canadian | Case-control | El Escorial criteria | 2 | PCR | 1/80 | 3/405 | 8 |
| Kwiatkowski, T. J., Jr.^(196)^ | 2009 | USA | Case-control | El Escorial criteria | 1 | WES | 12/290 | 0/293 | 6 |
| Ticozzi, N.^(197)^ | 2009 | Italy | Case-control | The revised El Escorial criteria | 1 | PCR,SSCP | 5/94 | / | 6 |
| Corrado, L^.(198)^ | 2010 | Italy | Case-control | The revised El Escorial criteria | 1 | PCR | 2/45 | 7/964 | 6 |
| Damme, P. V.^(199)^ | 2010 | Belgium | case series | The revised El Escorial criteria | 1 | PCR | 4/28 | / | 7 |
| DeJesusHernandez, M.^(200)^ | 2010 | USA | case series | El Escorial criteria | 1 | PCR | 0/17 | 2/99 | 6 |
| Hewitt, C.^(201)^ | 2010 | Britain | Case-control | El Escorial criteria | 2 | PCR | 3/42 | 3/117 | 7 |
| Yan, J.^(202)^ | 2010 | USA | Case-control | El Escorial criteria | 1 | WES | 23/393 | 0/41 | 7 |
| Min-Jung Kwon^(91)^ | 2011 | Korea | Cohort study | The revised El Escorial criteria | 1 | PCR | 1/9 | 6/29 | 6 |
| Drepper, C.^(203)^ | 2011 | Germany | Case-control | El Escorial criteria | 1 | PCR | 1/41 | 1/594 | 7 |
| Rutherford, N. J.^(204)^ | 2012 | USA | Case-control | The revised El Escorial criteria | 1 | PCR, RT-PCR | 2/148 | 0/482 | 5 |
| Sproviero, W.^(205)^ | 2012 | Italy | case series | El Escorial criteria | 1 | PCR | / | 4/327 | 7 |
| Marka van Blitterswijk^(95)^ | 2012 | the Netherlands | HB | El Escorial criteria | 1 | PCR | 35/97 | 87/1192 | 6 |
| Serena Lattante^(94)^ | 2012 | Italy | Case-control | El Escorial criteria | 1 | PCR | 0/48 | 3/480 | 8 |
| Zou, Z. Y.^(206)^ | 2013 | China | Case-control | The revised El Escorial criteria | 1 | PCR | 6/10 | 8/210 | 6 |
| Lee, Y. C.^(98)^ | 2013 | China | Case-control | El Escorial criteria | 1 | PCR | 2/30 | 2/131 | 7 |
| Sabatelli, M.^(207)^ | 2013 | Italy | case series | El Escorial criteria | 1 | PCR | 1/40 | 4/380 | 8 |
| Aslıhan Özoguz^(102)^ | 2014 | Turkey | Cohort study | El Escorial criteria | 1 | PCR,sanger sequencing | 4/82 | 0/361 | 6 |
| Tarlarini, C^.(208)^ | 2015 | Italy | case series | El Escorial criteria | 1 | PCR | 2/40 | 6/500 | 7 |
| Janet Cady^(105)^ | 2015 | USA | Case-control | El Escorial criteria | 1 | Sanger sequencing | 1/84 | 3/698 | 7 |
| Akiyama, T.^(209)^ | 2016 | Japan | case series | El Escorial criteria | 1 | PCR | 12/80 | / | 7 |
| Morgan, S.^(108)^ | 2017 | Britain | Case-control | El Escorial criteria | 2 | NGS | / | 5/1126 | 8 |
| Emily P. McCann^(107)^ | 2017 | Australia | Cohort study | El Escorial criteria | 1 | PCR | 5/267 | 27/746 | 7 |
| Müller, K.^(109)^ | 2018 | Germany | case series | El Escorial criteria | 9 | fragment length analysis, PCR, Southern blotting, Sanger sequencing and WES | 13/301 | / | 8 |
| Maurizio Grassano^(112)^ | 2020 | Germany | Case-control | The revised El Escorial criteria | 1 | PCR | 0/107 | 2/852 | 7 |
| W. Chen^(113)^ | 2020 | China | Case-control | El Escorial criteria | 1 | Sanger sequencing | 1/15 | 3/253 | 6 |
| Suzanna Edgar^(115)^ | 2021 | Malaya | Case-control | The revised El Escorial criteria | 1 | Sanger sequencing | 0/3 | 0/98 | 7 |
| W. Liu^(116)^ | 2021 | China | Case-control | The revised El Escorial criteria | 1 | PCR | 5/242 | / | 7 |
| Yong-Ping Chen^(114)^ | 2021 | China | Case-control | El Escorial criteria | 1 | WES and PCR | 4/64 | 15/1523 | 8 |
| Zhen Liu^(117)^ | 2021 | China | Case-control | The revised El Escorial criteria | 1 | PCR | 3/62 | 0/691 | 7 |

RT-PCR:Reverse transcription- Polymerase chain reaction

**Supplementary Table 31 the information of studies investigating *UBQLN2* gene mutation involved this study**

| First author | Publish year | Population | Study design | Diagnostic  category | Number of centers | Detection methods | Mutation rate | | NOS |
| --- | --- | --- | --- | --- | --- | --- | --- | --- | --- |
|  |  |  |  |  |  |  | FALS(n/N) | SALS(n/N) |  |
| Daoud, H.^(210)^ | 2012 | French and French Canadian | Case-control | El Escorial criteria | 1 | PCR | 1/190 | 6/475 | 7 |
| van Doormaal, P. T.^(211)^ | 2012 | Netherland | Case series | El Escorial criteria | 1 | PCR | 0/92 | / | 7 |
| Millecamps, S.^(212)^ | 2012 | French | Case-control | The revised El Escorial criteria | 1 | NA | 1/130 | 0/240 | 7 |
| Dillen, L.^(213)^ | 2013 | Belgium,Bulgarian | Case-control | The revised El Escorial criteria | 1 | Sanger sequencing | 0/33 | 4/124 | 6 |
| Gellera, C.^(214)^ | 2013 | Italy | Case-control | The revised El Escorial criteria | 6 | PCR | 4/226 | 1/819 | 7 |
| Lattante, S.^(215)^ | 2013 | French | Case series | El Escorial criteria | 1 | PCR | 0/28 | 0/10 | 5 |
| Lee, Y. C.^(98)^ | 2013 | China | HB | El Escorial criteria |  | PCR | 0/30 | 0/131 | 7 |
| Kim, H. J.^(216)^ | 2014 | Korea | Case-control | El Escorial criteria | 1 | PCR | 0/9 | 2/258 | 6 |
| McLaughlin, R. L.^(217)^ | 2014 | Ireland | Case series | The revised El Escorial criteria | 1 | PCR | 0/7 | 2/143 | 6 |
| Özoğuz, A.^(218)^ | 2015 | Turkey | Case-control | El Escorial criteria | 1 | PCR and Sanger sequencing | 2/82 | 2/361 | 6 |
| Huang, X.^(219)^ | 2017 | China | Case-control | El Escorial criteria | 1 | PCR | / | 2/515 | 7 |
| McCann, E. P.^(220)^ | 2017 | Australia | Case-control | El Escorial criteria | 3 | TP-PCR | 2/267 | 8/746 | 7 |
| Scotter, E. L.^(221)^ | 2017 | New Zealand | Case-control | The revised El Escorial criteria | 1 | TP-PCR and Sanger sequencing | / | 2/19 | 5 |
| Morgan, S.^(108)^ | 2017 | Britain | Case-control | El Escorial criteria | 2 | NGS | / | 1/1126 | 8 |
| Müller, K.^(109)^ | 2018 | Germany | case series | El Escorial criteria | 9 | fragment length analysis, PCR, Southern blotting, Sanger sequencing and WES | 2/301 | / | 8 |
| Tripolszki, K.^(191)^ | 2019 | Hungary | Case series | The revised El Escorial criteria | 1 | PCR-SSCP | 0/10 | 2/107 | 6 |
| Yong-Ping Chen^(114)^ | 2021 | China | Case-control | El Escorial criteria | 1 | WES and PCR | 1/64 | 3/1523 | 8 |

**Supplementary Table 32 the information of studies investigating *VCP* gene mutation involved this study**

| First author | Publish year | Population | Study design | Diagnostic  category | Number of centers | Detection methods | Mutation rate | | NOS |
| --- | --- | --- | --- | --- | --- | --- | --- | --- | --- |
|  |  |  |  |  |  |  | FALS(n/N) | SALS(n/N) |  |
| Johnson, J. O.^(222)^ | 2010 | USA,Italy | Case-control | El Escorial criteria | 2 | WES | 9/210 | 8/78 | 7 |
| Koppers, M.^(223)^ | 2012 | Dutch | Case-control | El Escorial criteria | 1 | PCR | 2/80 | 4/377 | 8 |
| Miller, J. W.^(224)^ | 2012 | British individuals and of Northern European origin | Case series | El Escorial criteria | 1 | PCR | 0/75 | 0/251 | 6 |
| Abramzon, Y.^(225)^ | 2012 | USA,Italy | Case-control | El Escorial criteria | 2 | PCR | / | 3/701 | 6 |
| Tiloca, C.^(226)^ | 2012 | Italy | Case-control | The revised El Escorial criteria | 6 | PCR | 2/166 | / | 6 |
| Williams, K. L.(227) | 2012 | European origin | Case series | El Escorial criteria | 1 | PCR | 1/131 | 0/48 | 7 |
| Zou, Z. Y.^(228)^ | 2013 | China | Case-control | The revised El Escorial criteria | 1 | PCR | 0/20 | 2/324 | 7 |
| Lee, Y. C.^(98)^ | 2013 | China | Case-control | El Escorial criteria | 1 | PCR | 0/30 | 0/131 | 7 |
| Hirano, M.^(229)^ | 2015 | Japan | Case-control | El Escorial criteria | 1 | WES | / | 1/75 | 5 |
| Morgan, S.^(108)^ | 2017 | Britain | Case-control | El Escorial criteria | 2 | NGS | / | 2/1126 | 8 |
| Naruse, H.^(230)^ | 2018 | Japan | Case-control | The revised El Escorial criteria | 1 | PCR | / | 1/68 | 7 |
| Müller, K.^(109)^ | 2018 | Germany | case series | El Escorial criteria | 9 | fragment length analysis, PCR, Southern blotting, Sanger sequencing and WES | 1/301 | / | 8 |
| Yong-Ping Chen^(114)^ | 2021 | China | Case-control | El Escorial criteria | 1 | WES and PCR | 2/64 | 2/1523 | 8 |

**References**

1. McGuire V, Longstreth WT, Jr., Nelson LM, Koepsell TD, Checkoway H, Morgan MS, et al. Occupational exposures and amyotrophic lateral sclerosis. A population-based case-control study. Am J Epidemiol. 1997;145(12):1076-88.

2. Fang F, Quinlan P, Ye W, Barber MK, Umbach DM, Sandler DP, et al. Workplace exposures and the risk of amyotrophic lateral sclerosis. Environ Health Perspect. 2009;117(9):1387-92.

3. Furby A, Beauvais K, Kolev I, Rivain JG, Sébille V. Rural environment and risk factors of amyotrophic lateral sclerosis: a case-control study. J Neurol. 2010;257(5):792-8.

4. Das K, Nag C, Ghosh M. Familial, environmental, and occupational risk factors in development of amyotrophic lateral sclerosis. N Am J Med Sci. 2012;4(8):350-5.

5. Pamphlett R. Exposure to environmental toxins and the risk of sporadic motor neuron disease: an expanded Australian case-control study. Eur J Neurol. 2012;19(10):1343-8.

6. Andrew AS, Caller TA, Tandan R, Duell EJ, Henegan PL, Field NC, et al. Environmental and Occupational Exposures and Amyotrophic Lateral Sclerosis in New England. Neurodegener Dis. 2017;17(2-3):110-6.

7. Koeman T, Slottje P, Schouten LJ, Peters S, Huss A, Veldink JH, et al. Occupational exposure and amyotrophic lateral sclerosis in a prospective cohort. Occup Environ Med. 2017;74(8):578-85.

8. Peters TL, Kamel F, Lundholm C, Feychting M, Weibull CE, Sandler DP, et al. Occupational exposures and the risk of amyotrophic lateral sclerosis. Occup Environ Med. 2017;74(2):87-92.

9. Filippini T, Tesauro M, Fiore M, Malagoli C, Consonni M, Violi F, et al. Environmental and Occupational Risk Factors of Amyotrophic Lateral Sclerosis: A Population-Based Case-Control Study. Int J Environ Res Public Health. 2020;17(8).

10. Bellavia A, Dickerson AS, Rotem RS, Hansen J, Gredal O, Weisskopf MG. Joint and interactive effects between health comorbidities and environmental exposures in predicting amyotrophic lateral sclerosis. Int J Hyg Environ Health. 2021;231:113655.

11. Goutman SA, Boss J, Godwin C, Mukherjee B, Feldman EL, Batterman SA. Associations of self-reported occupational exposures and settings to ALS: a case-control study. Int Arch Occup Environ Health. 2022;95(7):1567-86.

12. Vergara X, Mezei G, Kheifets L. Case-control study of occupational exposure to electric shocks and magnetic fields and mortality from amyotrophic lateral sclerosis in the US, 1991-1999. J Expo Sci Environ Epidemiol. 2015;25(1):65-71.

13. Fischer H, Kheifets L, Huss A, Peters TL, Vermeulen R, Ye W, et al. Occupational Exposure to Electric Shocks and Magnetic Fields and Amyotrophic Lateral Sclerosis in Sweden. Epidemiology. 2015;26(6):824-30.

14. Vinceti M, Malagoli C, Fabbi S, Kheifets L, Violi F, Poli M, et al. Magnetic fields exposure from high-voltage power lines and risk of amyotrophic lateral sclerosis in two Italian populations. Amyotroph Lateral Scler Frontotemporal Degener. 2017;18(7-8):583-9.

15. Peters S, Visser AE, D'Ovidio F, Beghi E, Chiò A, Logroscino G, et al. Associations of Electric Shock and Extremely Low-Frequency Magnetic Field Exposure With the Risk of Amyotrophic Lateral Sclerosis. Am J Epidemiol. 2019;188(4):796-805.

16. Binazzi A, Belli S, Uccelli R, Desiato MT, Talamanca IF, Antonini G, et al. An exploratory case-control study on spinal and bulbar forms of amyotrophic lateral sclerosis in the province of Rome. Amyotroph Lateral Scler. 2009;10(5-6):361-9.

17. Dickerson AS, Hansen J, Specht AJ, Gredal O, Weisskopf MG. Population-based study of amyotrophic lateral sclerosis and occupational lead exposure in Denmark. Occup Environ Med. 2019;76(4):208-14.

18. Peters S, Broberg K, Gallo V, Levi M, Kippler M, Vineis P, et al. Blood Metal Levels and Amyotrophic Lateral Sclerosis Risk: A Prospective Cohort. Ann Neurol. 2021;89(1):125-33.

19. Mitsumoto H, Garofalo DC, Gilmore M, Andrews L, Santella RM, Andrews H, et al. Case-control study in ALS using the National ALS Registry: lead and agricultural chemicals are potential risk factors. Amyotroph Lateral Scler Frontotemporal Degener. 2022;23(3-4):190-202.

20. Sørensen HT, Riis AH, Lash TL, Pedersen L. Statin use and risk of amyotrophic lateral sclerosis and other motor neuron disorders. Circ Cardiovasc Qual Outcomes. 2010;3(4):413-7.

21. Sutedja NA, van der Schouw YT, Fischer K, Sizoo EM, Huisman MH, Veldink JH, et al. Beneficial vascular risk profile is associated with amyotrophic lateral sclerosis. J Neurol Neurosurg Psychiatry. 2011;82(6):638-42.

22. Freedman DM, Kuncl RW, Cahoon EK, Rivera DR, Pfeiffer RM. Relationship of statins and other cholesterol-lowering medications and risk of amyotrophic lateral sclerosis in the US elderly. Amyotroph Lateral Scler Frontotemporal Degener. 2018;19(7-8):538-46.

23. Mariosa D, Kamel F, Bellocco R, Ronnevi LO, Almqvist C, Larsson H, et al. Antidiabetics, statins and the risk of amyotrophic lateral sclerosis. Eur J Neurol. 2020;27(6):1010-6.

24. Bjornevik K, O'Reilly É J, Cortese M, Furtado JD, Kolonel LN, Le Marchand L, et al. Pre-diagnostic plasma lipid levels and the risk of amyotrophic lateral sclerosis. Amyotroph Lateral Scler Frontotemporal Degener. 2021;22(1-2):133-43.

25. Diekmann K, Kuzma-Kozakiewicz M, Piotrkiewicz M, Gromicho M, Grosskreutz J, Andersen PM, et al. Impact of comorbidities and co-medication on disease onset and progression in a large German ALS patient group. J Neurol. 2020;267(7):2130-41.

26. Popat RA, Tanner CM, van den Eeden SK, Bernstein AL, Bloch DA, Leimpeter A, et al. Effect of non-steroidal anti-inflammatory medications on the risk of amyotrophic lateral sclerosis. Amyotroph Lateral Scler. 2007;8(3):157-63.

27. Lin FC, Tsai CP, Kuang-Wu Lee J, Wu MT, Tzu-Chi Lee C. Angiotensin-converting enzyme inhibitors and amyotrophic lateral sclerosis risk: a total population-based case-control study. JAMA Neurol. 2015;72(1):40-8.

28. Mariosa D, Kamel F, Bellocco R, Ye W, Fang F. Association between diabetes and amyotrophic lateral sclerosis in Sweden. Eur J Neurol. 2015;22(11):1436-42.

29. Seelen M, van Doormaal PT, Visser AE, Huisman MH, Roozekrans MH, de Jong SW, et al. Prior medical conditions and the risk of amyotrophic lateral sclerosis. J Neurol. 2014;261(10):1949-56.

30. Kioumourtzoglou MA, Rotem RS, Seals RM, Gredal O, Hansen J, Weisskopf MG. Diabetes Mellitus, Obesity, and Diagnosis of Amyotrophic Lateral Sclerosis: A Population-Based Study. JAMA Neurol. 2015;72(8):905-11.

31. D'Ovidio F, Rooney JPK, Visser AE, Manera U, Beghi E, Logroscino G, et al. Association between alcohol exposure and the risk of amyotrophic lateral sclerosis in the Euro-MOTOR study. J Neurol Neurosurg Psychiatry. 2019;90(1):11-9.

32. Nagel G, Peter RS, Rosenbohm A, Koenig W, Dupuis L, Rothenbacher D, et al. Association of Insulin-like Growth Factor 1 Concentrations with Risk for and Prognosis of Amyotrophic Lateral Sclerosis - Results from the ALS Registry Swabia. Sci Rep. 2020;10(1):736.

33. Chen H, Richard M, Sandler DP, Umbach DM, Kamel F. Head injury and amyotrophic lateral sclerosis. Am J Epidemiol. 2007;166(7):810-6.

34. Schmidt S, Kwee LC, Allen KD, Oddone EZ. Association of ALS with head injury, cigarette smoking and APOE genotypes. J Neurol Sci. 2010;291(1-2):22-9.

35. Peters TL, Fang F, Weibull CE, Sandler DP, Kamel F, Ye W. Severe head injury and amyotrophic lateral sclerosis. Amyotroph Lateral Scler Frontotemporal Degener. 2013;14(4):267-72.

36. Seals RM, Hansen J, Gredal O, Weisskopf MG. Physical Trauma and Amyotrophic Lateral Sclerosis: A Population-Based Study Using Danish National Registries. Am J Epidemiol. 2016;183(4):294-301.

37. Pupillo E, Poloni M, Bianchi E, Giussani G, Logroscino G, Zoccolella S, et al. Trauma and amyotrophic lateral sclerosis: a european population-based case-control study from the EURALS consortium. Amyotroph Lateral Scler Frontotemporal Degener. 2018;19(1-2):118-25.

38. Filippini T, Fiore M, Tesauro M, Malagoli C, Consonni M, Violi F, et al. Clinical and Lifestyle Factors and Risk of Amyotrophic Lateral Sclerosis: A Population-Based Case-Control Study. Int J Environ Res Public Health. 2020;17(3).

39. Andrew AS, Bradley WG, Peipert D, Butt T, Amoako K, Pioro EP, et al. Risk factors for amyotrophic lateral sclerosis: A regional United States case-control study. Muscle Nerve. 2021;63(1):52-9.

40. Beaudin M, Salachas F, Pradat PF, Dupré N. Environmental risk factors for amyotrophic lateral sclerosis: a case-control study in Canada and France. Amyotroph Lateral Scler Frontotemporal Degener. 2022;23(7-8):592-600.

41. Chen GX, Douwes J, van den Berg LH, Glass B, McLean D, t Mannetje AM. Sports and trauma as risk factors for Motor Neurone Disease: New Zealand case-control study. Acta Neurol Scand. 2022;145(6):770-85.

42. Abdel Magid HS, Topol B, McGuire V, Hinman JA, Kasarskis EJ, Nelson LM. Cardiovascular Diseases, Medications, and ALS: A Population-Based Case-Control Study. Neuroepidemiology. 2022;56(6):423-32.

43. Nelson LM, Matkin C, Longstreth WT, Jr., McGuire V. Population-based case-control study of amyotrophic lateral sclerosis in western Washington State. II. Diet. Am J Epidemiol. 2000;151(2):164-73.

44. Okamoto K, Kihira T, Kobashi G, Washio M, Sasaki S, Yokoyama T, et al. Fruit and vegetable intake and risk of amyotrophic lateral sclerosis in Japan. Neuroepidemiology. 2009;32(4):251-6.

45. Morozova N, Weisskopf MG, McCullough ML, Munger KL, Calle EE, Thun MJ, et al. Diet and amyotrophic lateral sclerosis. Epidemiology. 2008;19(2):324-37.

46. Beghi E, Pupillo E, Messina P, Giussani G, Chiò A, Zoccolella S, et al. Coffee and amyotrophic lateral sclerosis: a possible preventive role. Am J Epidemiol. 2011;174(9):1002-8.

47. de Jong SW, Huisman MH, Sutedja NA, van der Kooi AJ, de Visser M, Schelhaas HJ, et al. Smoking, alcohol consumption, and the risk of amyotrophic lateral sclerosis: a population-based study. Am J Epidemiol. 2012;176(3):233-9.

48. Jin Y, Oh K, Oh SI, Baek H, Kim SH, Park Y. Dietary intake of fruits and beta-carotene is negatively associated with amyotrophic lateral sclerosis risk in Koreans: a case-control study. Nutr Neurosci. 2014;17(3):104-8.

49. Malek AM, Barchowsky A, Bowser R, Heiman-Patterson T, Lacomis D, Rana S, et al. Exposure to hazardous air pollutants and the risk of amyotrophic lateral sclerosis. Environ Pollut. 2015;197:181-6.

50. O'Reilly É J, Bjornevik K, Schwarzschild MA, McCullough ML, Kolonel LN, Le Marchand L, et al. Pre-diagnostic plasma urate and the risk of amyotrophic lateral sclerosis. Amyotroph Lateral Scler Frontotemporal Degener. 2018;19(3-4):194-200.

51. Rosenbohm A, Nagel G, Peter RS, Brehme T, Koenig W, Dupuis L, et al. Association of Serum Retinol-Binding Protein 4 Concentration With Risk for and Prognosis of Amyotrophic Lateral Sclerosis. JAMA Neurol. 2018;75(5):600-7.

52. Visser AE, Rooney JPK, D'Ovidio F, Westeneng HJ, Vermeulen RCH, Beghi E, et al. Multicentre, cross-cultural, population-based, case-control study of physical activity as risk factor for amyotrophic lateral sclerosis. J Neurol Neurosurg Psychiatry. 2018;89(8):797-803.

53. Opie-Martin S, Jones A, Iacoangeli A, Al-Khleifat A, Oumar M, Shaw PJ, et al. UK case control study of smoking and risk of amyotrophic lateral sclerosis. Amyotroph Lateral Scler Frontotemporal Degener. 2020;21(3-4):222-7.

54. Farrugia Wismayer M, Borg R, Farrugia Wismayer A, Bonavia K, Vella M, Pace A, et al. Occupation and amyotrophic lateral sclerosis risk: a case-control study in the isolated island population of Malta. Amyotroph Lateral Scler Frontotemporal Degener. 2021;22(7-8):528-34.

55. Kamel F, Umbach DM, Munsat TL, Shefner JM, Hu H, Sandler DP. Lead exposure and amyotrophic lateral sclerosis. Epidemiology. 2002;13(3):311-9.

56. Lian L, Liu M, Cui L, Guan Y, Liu T, Cui B, et al. Environmental risk factors and amyotrophic lateral sclerosis (ALS): A case-control study of ALS in China. J Clin Neurosci. 2019;66:12-8.

57. Weisskopf MG, Cudkowicz ME, Johnson N. Military Service and Amyotrophic Lateral Sclerosis in a Population-based Cohort. Epidemiology. 2015;26(6):831-8.

58. D'Ovidio F, d'Errico A, Calvo A, Costa G, Chiò A. Occupations and amyotrophic lateral sclerosis: are jobs exposed to the general public at higher risk? Eur J Public Health. 2017;27(4):643-7.

59. Huisman MH, Seelen M, de Jong SW, Dorresteijn KR, van Doormaal PT, van der Kooi AJ, et al. Lifetime physical activity and the risk of amyotrophic lateral sclerosis. J Neurol Neurosurg Psychiatry. 2013;84(9):976-81.

60. Pupillo E, Messina P, Giussani G, Logroscino G, Zoccolella S, Chiò A, et al. Physical activity and amyotrophic lateral sclerosis: a European population-based case-control study. Ann Neurol. 2014;75(5):708-16.

61. Yu Y, Su FC, Callaghan BC, Goutman SA, Batterman SA, Feldman EL. Environmental risk factors and amyotrophic lateral sclerosis (ALS): a case-control study of ALS in Michigan. PLoS One. 2014;9(6):e101186.

62. Gallo V, Vanacore N, Bueno-de-Mesquita HB, Vermeulen R, Brayne C, Pearce N, et al. Physical activity and risk of Amyotrophic Lateral Sclerosis in a prospective cohort study. Eur J Epidemiol. 2016;31(3):255-66.

63. Scarmeas N, Shih T, Stern Y, Ottman R, Rowland LP. Premorbid weight, body mass, and varsity athletics in ALS. Neurology. 2002;59(5):773-5.

64. O'Reilly É J, Wang H, Weisskopf MG, Fitzgerald KC, Falcone G, McCullough ML, et al. Premorbid body mass index and risk of amyotrophic lateral sclerosis. Amyotroph Lateral Scler Frontotemporal Degener. 2013;14(3):205-11.

65. Pupillo E, Bianchi E, Chiò A, Casale F, Zecca C, Tortelli R, et al. Amyotrophic lateral sclerosis and food intake. Amyotroph Lateral Scler Frontotemporal Degener. 2018;19(3-4):267-74.

66. Morahan JM, Pamphlett R. Amyotrophic lateral sclerosis and exposure to environmental toxins: an Australian case-control study. Neuroepidemiology. 2006;27(3):130-5.

67. Korner S, Kammeyer J, Zapf A, Kuzma-Kozakiewicz M, Piotrkiewicz M, Kuraszkiewicz B, et al. Influence of Environment and Lifestyle on Incidence and Progress of Amyotrophic Lateral Sclerosis in A German ALS Population. Aging Dis. 2019;10(2):205-16.

68. Jones CT, Swingler RJ, Simpson SA, Brock DJ. Superoxide dismutase mutations in an unselected cohort of Scottish amyotrophic lateral sclerosis patients. J Med Genet. 1995;32(4):290-2.

69. Pramatarova A, Figlewicz DA, Krizus A, Han FY, Ceballos-Picot I, Nicole A, et al. Identification of new mutations in the Cu/Zn superoxide dismutase gene of patients with familial amyotrophic lateral sclerosis. Am J Hum Genet. 1995;56(3):592-6.

70. Cudkowicz ME, McKenna-Yasek D, Sapp PE, Chin W, Geller B, Hayden DL, et al. Epidemiology of mutations in superoxide dismutase in amyotrophic lateral sclerosis. Ann Neurol. 1997;41(2):210-21.

71. Andersen PM, Nilsson P, Keränen ML, Forsgren L, Hägglund J, Karlsborg M, et al. Phenotypic heterogeneity in motor neuron disease patients with CuZn-superoxide dismutase mutations in Scandinavia. Brain. 1997;120 ( Pt 10):1723-37.

72. Jackson M, Al-Chalabi A, Enayat ZE, Chioza B, Leigh PN, Morrison KE. Copper/zinc superoxide dismutase 1 and sporadic amyotrophic lateral sclerosis: analysis of 155 cases and identification of a novel insertion mutation. Ann Neurol. 1997;42(5):803-7.

73. Boukaftane Y, Khoris J, Moulard B, Salachas F, Meininger V, Malafosse A, et al. Identification of six novel SOD1 gene mutations in familial amyotrophic lateral sclerosis. Can J Neurol Sci. 1998;25(3):192-6.

74. Aguirre T, Matthijs G, Robberecht W, Tilkin P, Cassiman JJ. Mutational analysis of the Cu/Zn superoxide dismutase gene in 23 familial and 69 sporadic cases of amyotrophic lateral sclerosis in Belgium. Eur J Hum Genet. 1999;7(5):599-602.

75. Skvortsova VI, Limborska SA, Slominsky PA, Levitskaya NI, Levitsky GN, Shadrina MI, et al. Sporadic ALS associated with the D90A Cu,Zn superoxide dismutase mutation in Russia. Eur J Neurol. 2001;8(2):167-72.

76. Chioza BA, Ujfalusy A, Csiszar K, Leigh PN, Powell JF, Radunović A. Mutations in the lysyl oxidase gene are not associated with amyotrophic lateral sclerosis. Amyotroph Lateral Scler Other Motor Neuron Disord. 2001;2(2):93-7.

77. Gellera C. Genetics of ALS in Italian families. Amyotroph Lateral Scler Other Motor Neuron Disord. 2001;2 Suppl 1:S43-6.

78. García-Redondo A, Bustos F, Juan YSB, Del Hoyo P, Jiménez S, Campos Y, et al. Molecular analysis of the superoxide dismutase 1 gene in Spanish patients with sporadic or familial amyotrophic lateral sclerosis. Muscle Nerve. 2002;26(2):274-8.

79. Sato T, Yamamoto Y, Nakanishi T, Fukada K, Sugai F, Zhou Z, et al. Identification of two novel mutations in the Cu/Zn superoxide dismutase gene with familial amyotrophic lateral sclerosis: mass spectrometric and genomic analyses. J Neurol Sci. 2004;218(1-2):79-83.

80. Battistini S, Giannini F, Greco G, Bibbò G, Ferrera L, Marini V, et al. SOD1 mutations in amyotrophic lateral sclerosis. Results from a multicenter Italian study. J Neurol. 2005;252(7):782-8.

81. Corrado L, D'Alfonso S, Bergamaschi L, Testa L, Leone M, Nasuelli N, et al. SOD1 gene mutations in Italian patients with Sporadic Amyotrophic Lateral Sclerosis (ALS). Neuromuscul Disord. 2006;16(11):800-4.

82. Gamez J, Corbera-Bellalta M, Nogales G, Raguer N, García-Arumí E, Badia-Canto M, et al. Mutational analysis of the Cu/Zn superoxide dismutase gene in a Catalan ALS population: should all sporadic ALS cases also be screened for SOD1? J Neurol Sci. 2006;247(1):21-8.

83. Eisen A, Mezei MM, Stewart HG, Fabros M, Gibson G, Andersen PM. SOD1 gene mutations in ALS patients from British Columbia, Canada: clinical features, neurophysiology and ethical issues in management. Amyotroph Lateral Scler. 2008;9(2):108-19.

84. Luquin N, Yu B, Trent RJ, Morahan JM, Pamphlett R. An analysis of the entire SOD1 gene in sporadic ALS. Neuromuscul Disord. 2008;18(7):545-52.

85. van Es MA, Dahlberg C, Birve A, Veldink JH, van den Berg LH, Andersen PM. Large-scale SOD1 mutation screening provides evidence for genetic heterogeneity in amyotrophic lateral sclerosis. J Neurol Neurosurg Psychiatry. 2010;81(5):562-6.

86. Rabe M, Felbecker A, Waibel S, Steinbach P, Winter P, Müller U, et al. The epidemiology of CuZn-SOD mutations in Germany: a study of 217 families. J Neurol. 2010;257(8):1298-302.

87. Laaksovirta H, Peuralinna T, Schymick JC, Scholz SW, Lai SL, Myllykangas L, et al. Chromosome 9p21 in amyotrophic lateral sclerosis in Finland: a genome-wide association study. Lancet Neurol. 2010;9(10):978-85.

88. Millecamps S, Salachas F, Cazeneuve C, Gordon P, Bricka B, Camuzat A, et al. SOD1, ANG, VAPB, TARDBP, and FUS mutations in familial amyotrophic lateral sclerosis: genotype-phenotype correlations. J Med Genet. 2010;47(8):554-60.

89. Akimoto C, Morita M, Atsuta N, Sobue G, Nakano I. High-Resolution Melting (HRM) Analysis of the Cu/Zn Superoxide Dismutase (SOD1) Gene in Japanese Sporadic Amyotrophic Lateral Sclerosis (SALS) Patients. Neurol Res Int. 2011;2011:165415.

90. Brown JA, Min J, Staropoli JF, Collin E, Bi S, Feng X, et al. SOD1, ANG, TARDBP and FUS mutations in amyotrophic lateral sclerosis: a United States clinical testing lab experience. Amyotroph Lateral Scler. 2012;13(2):217-22.

91. Kwon MJ, Baek W, Ki CS, Kim HY, Koh SH, Kim JW, et al. Screening of the SOD1, FUS, TARDBP, ANG, and OPTN mutations in Korean patients with familial and sporadic ALS. Neurobiol Aging. 2012;33(5):1017.e17-23.

92. Piaceri I, Del Mastio M, Tedde A, Bagnoli S, Latorraca S, Massaro F, et al. Clinical heterogeneity in Italian patients with amyotrophic lateral sclerosis. Clin Genet. 2012;82(1):83-7.

93. Chiò A, Calvo A, Mazzini L, Cantello R, Mora G, Moglia C, et al. Extensive genetics of ALS: a population-based study in Italy. Neurology. 2012;79(19):1983-9.

94. Lattante S, Conte A, Zollino M, Luigetti M, Del Grande A, Marangi G, et al. Contribution of major amyotrophic lateral sclerosis genes to the etiology of sporadic disease. Neurology. 2012;79(1):66-72.

95. van Blitterswijk M, van Es MA, Hennekam EA, Dooijes D, van Rheenen W, Medic J, et al. Evidence for an oligogenic basis of amyotrophic lateral sclerosis. Hum Mol Genet. 2012;21(17):3776-84.

96. Pasini E, De Biase D, Visani M, Morandi L, Danesi F, Boschetti E, et al. Activity of the novel T137A SOD1 mutation in amyotrophic lateral sclerosis patients. Future Neurology. 2012;7(4):499-503.

97. Kenna KP, McLaughlin RL, Byrne S, Elamin M, Heverin M, Kenny EM, et al. Delineating the genetic heterogeneity of ALS using targeted high-throughput sequencing. J Med Genet. 2013;50(11):776-83.

98. Lee YC, Tsai CP, Soong BS. Extensive genetic analysis in a Taiwanese cohort with amyotrophic lateral sclerosis. Amyotrophic Lateral Sclerosis and Frontotemporal Degeneration. 2013;14:160.

99. Lysogorskaia E, Abramycheva N, Rossokhin A, Zakharova M, Illarioshkin S. Molecular genetic analysis in Russian patients with amyotrophic lateral sclerosis. Amyotrophic Lateral Sclerosis and Frontotemporal Degeneration. 2013;14:161.

100. Couthouis J, Raphael AR, Daneshjou R, Gitler AD. Targeted exon capture and sequencing in sporadic amyotrophic lateral sclerosis. PLoS Genet. 2014;10(10):e1004704.

101. Soong BW, Lin KP, Guo YC, Lin CC, Tsai PC, Liao YC, et al. Extensive molecular genetic survey of Taiwanese patients with amyotrophic lateral sclerosis. Neurobiol Aging. 2014;35(10):2423.e1-6.

102. Özoğuz A, Uyan T, Birdal G, Iskender C, Kartal E, Lahut S, et al. The distinct genetic pattern of ALS in Turkey and novel mutations. Neurobiology of Aging. 2015;36(4):1764.e9-.e18.

103. Niemann S, Joos H, Meyer T, Vielhaber S, Reuner U, Gleichmann M, et al. Familial ALS in Germany: origin of the R115G SOD1 mutation by a founder effect. J Neurol Neurosurg Psychiatry. 2004;75(8):1186-8.

104. Zou ZY, Liu MS, Li XG, Cui LY. The distinctive genetic architecture of ALS in mainland China. J Neurol Neurosurg Psychiatry. 2016;87(8):906-7.

105. Cady J, Allred P, Bali T, Pestronk A, Goate A, Miller TM, et al. Amyotrophic lateral sclerosis onset is influenced by the burden of rare variants in known amyotrophic lateral sclerosis genes. Ann Neurol. 2015;77(1):100-13.

106. Chadi G, Maximino JR, Jorge FMH, Borba FC, Gilio JM, Callegaro D, et al. Genetic analysis of patients with familial and sporadic amyotrophic lateral sclerosis in a Brazilian Research Center. Amyotroph Lateral Scler Frontotemporal Degener. 2017;18(3-4):249-55.

107. McCann EP, Williams KL, Fifita JA, Tarr IS, O'Connor J, Rowe DB, et al. The genotype–phenotype landscape of familial amyotrophic lateral sclerosis in Australia. Clinical Genetics. 2017;92(3):259-66.

108. Morgan S, Shatunov A, Sproviero W, Jones AR, Shoai M, Hughes D, et al. A comprehensive analysis of rare genetic variation in amyotrophic lateral sclerosis in the UK. Brain. 2017;140(6):1611-8.

109. Müller K, Brenner D, Weydt P, Meyer T, Grehl T, Petri S, et al. Comprehensive analysis of the mutation spectrum in 301 German ALS families. J Neurol Neurosurg Psychiatry. 2018;89(8):817-27.

110. Zhang H, Cai W, Chen S, Liang J, Wang Z, Ren Y, et al. Screening for possible oligogenic pathogenesis in Chinese sporadic ALS patients. Amyotroph Lateral Scler Frontotemporal Degener. 2018;19(5-6):419-25.

111. Liu ZJ, Lin HX, Wei Q, Zhang QJ, Chen CX, Tao QQ, et al. Genetic spectrum and variability in Chinese patients with amyotrophic lateral sclerosis. Aging and Disease. 2019;10(6):1199-206.

112. Grassano M, Calvo A, Moglia C, Brunetti M, Barberis M, Sbaiz L, et al. Mutational Analysis of Known ALS Genes in an Italian Population-Based Cohort. Neurology. 2021;96(4):e600-e9.

113. Chen W, Xie Y, Zheng M, Lin J, Huang P, Pei Z, et al. Clinical and genetic features of patients with amyotrophic lateral sclerosis in southern China. Eur J Neurol. 2020;27(6):1017-22.

114. Chen YP, Yu SH, Wei QQ, Cao B, Gu XJ, Chen XP, et al. Role of genetics in amyotrophic lateral sclerosis: a large cohort study in Chinese mainland population. J Med Genet. 2021.

115. Edgar S, Ellis M, Abdul-Aziz NA, Goh KJ, Shahrizaila N, Kennerson ML, et al. Mutation analysis of SOD1, C9orf72, TARDBP and FUS genes in ethnically-diverse Malaysian patients with amyotrophic lateral sclerosis (ALS). Neurobiology of Aging. 2021.

116. Liu W, Li X, Sun Y, Yu X, Wang Y, Liu N, et al. Genotype-phenotype correlations in a chinese population with familial amyotrophic lateral sclerosis. Neurol Res. 2021:1-11.

117. Liu Z, Yuan Y, Wang M, Ni J, Li W, Huang L, et al. Mutation spectrum of amyotrophic lateral sclerosis in Central South China. Neurobiology of Aging. 2021.

118. Fecto F, Yan J, Vemula SP, Liu E, Yang Y, Chen W, et al. SQSTM1 mutations in familial and sporadic amyotrophic lateral sclerosis. Arch Neurol. 2011;68(11):1440-6.

119. Hirano M, Nakamura Y, Saigoh K, Sakamoto H, Ueno S, Isono C, et al. Mutations in the gene encoding p62 in Japanese patients with amyotrophic lateral sclerosis. Neurology. 2013;80(5):458-63.

120. Teyssou E, Takeda T, Lebon V, Boillée S, Doukouré B, Bataillon G, et al. Mutations in SQSTM1 encoding p62 in amyotrophic lateral sclerosis: genetics and neuropathology. Acta Neuropathol. 2013;125(4):511-22.

121. Yang Y, Tang L, Zhang N, Pan L, Hadano S, Fan D. Six SQSTM1 mutations in a Chinese amyotrophic lateral sclerosis cohort. Amyotroph Lateral Scler Frontotemporal Degener. 2015;16(5-6):378-84.

122. Kwok CT, Wang HY, Morris AG, Smith B, Shaw C, de Belleroche J. VCP mutations are not a major cause of familial amyotrophic lateral sclerosis in the UK. J Neurol Sci. 2015;349(1-2):209-13.

123. Dols-Icardo O, García-Redondo A, Rojas-García R, Borrego-Hernández D, Illán-Gala I, Muñoz-Blanco JL, et al. Analysis of known amyotrophic lateral sclerosis and frontotemporal dementia genes reveals a substantial genetic burden in patients manifesting both diseases not carrying the C9orf72 expansion mutation. J Neurol Neurosurg Psychiatry. 2018;89(2):162-8.

124. Yilmaz R, Müller K, Brenner D, Volk AE, Borck G, Hermann A, et al. SQSTM1/p62 variants in 486 patients with familial ALS from Germany and Sweden. Neurobiol Aging. 2020;87:139.e9-.e15.

125. Narain P, Pandey A, Gupta S, Gomes J, Bhatia R, Vivekanandan P. Targeted next-generation sequencing reveals novel and rare variants in Indian patients with amyotrophic lateral sclerosis. Neurobiol Aging. 2018;71:265.e9-.e14.

126. Pensato V, Magri S, Bella ED, Tannorella P, Bersano E, Sorarù G, et al. Sorting Rare ALS Genetic Variants by Targeted Re-Sequencing Panel in Italian Patients: OPTN, VCP, and SQSTM1 Variants Account for 3% of Rare Genetic Forms. J Clin Med. 2020;9(2).

127. McCann EP, Henden L, Fifita JA, Zhang KY, Grima N, Bauer DC, et al. Evidence for polygenic and oligogenic basis of Australian sporadic amyotrophic lateral sclerosis. J Med Genet. 2020.

128. Rutherford NJ, Zhang YJ, Baker M, Gass JM, Finch NA, Xu YF, et al. Novel mutations in TARDBP (TDP-43) in patients with familial amyotrophic lateral sclerosis. PLoS Genet. 2008;4(9):e1000193.

129. Sreedharan J, Blair IP, Tripathi VB, Hu X, Vance C, Rogelj B, et al. TDP-43 mutations in familial and sporadic amyotrophic lateral sclerosis. Science. 2008;319(5870):1668-72.

130. Kabashi E, Valdmanis PN, Dion P, Spiegelman D, McConkey BJ, Vande Velde C, et al. TARDBP mutations in individuals with sporadic and familial amyotrophic lateral sclerosis. Nat Genet. 2008;40(5):572-4.

131. Guerreiro RJ, Schymick JC, Crews C, Singleton A, Hardy J, Traynor BJ. TDP-43 is not a common cause of sporadic amyotrophic lateral sclerosis. PLoS One. 2008;3(6):e2450.

132. Yokoseki A, Shiga A, Tan CF, Tagawa A, Kaneko H, Koyama A, et al. TDP-43 mutation in familial amyotrophic lateral sclerosis. Ann Neurol. 2008;63(4):538-42.

133. Del Bo R, Ghezzi S, Corti S, Pandolfo M, Ranieri M, Santoro D, et al. TARDBP (TDP-43) sequence analysis in patients with familial and sporadic ALS: identification of two novel mutations. Eur J Neurol. 2009;16(6):727-32.

134. Kamada M, Maruyama H, Tanaka E, Morino H, Wate R, Ito H, et al. Screening for TARDBP mutations in Japanese familial amyotrophic lateral sclerosis. J Neurol Sci. 2009;284(1-2):69-71.

135. Ticozzi N, LeClerc AL, van Blitterswijk M, Keagle P, McKenna-Yasek DM, Sapp PC, et al. Mutational analysis of TARDBP in neurodegenerative diseases. Neurobiol Aging. 2011;32(11):2096-9.

136. Corrado L, Ratti A, Gellera C, Buratti E, Castellotti B, Carlomagno Y, et al. High frequency of TARDBP gene mutations in Italian patients with amyotrophic lateral sclerosis. Hum Mutat. 2009;30(4):688-94.

137. Conforti FL, Sproviero W, Simone IL, Mazzei R, Valentino P, Ungaro C, et al. TARDBP gene mutations in south Italian patients with amyotrophic lateral sclerosis. J Neurol Neurosurg Psychiatry. 2011;82(5):587-8.

138. Xiong HL, Wang JY, Sun YM, Wu JJ, Chen Y, Qiao K, et al. Association between novel TARDBP mutations and Chinese patients with amyotrophic lateral sclerosis. BMC Med Genet. 2010;11:8.

139. Orrù S, Manolakos E, Orrù N, Kokotas H, Mascia V, Carcassi C, et al. High frequency of the TARDBP p.Ala382Thr mutation in Sardinian patients with amyotrophic lateral sclerosis. Clin Genet. 2012;81(2):172-8.

140. Huang R, Fang DF, Ma MY, Guo XY, Zhao B, Zeng Y, et al. TARDBP gene mutations among Chinese patients with sporadic amyotrophic lateral sclerosis. Neurobiol Aging. 2012;33(5):1015.e1-6.

141. Iida A, Kamei T, Sano M, Oshima S, Tokuda T, Nakamura Y, et al. Large-scale screening of TARDBP mutation in amyotrophic lateral sclerosis in Japanese. Neurobiol Aging. 2012;33(4):786-90.

142. Czell D, Andersen PM, Morita M, Neuwirth C, Perren F, Weber M. Phenotypes in Swiss patients with familial ALS carrying TARDBP mutations. Neurodegener Dis. 2013;12(3):150-5.

143. Ye CH, Lu XL, Zheng MY, Zhen J, Li ZP, Shi L, et al. Absence of mutations in exon 6 of the TARDBP gene in 207 Chinese patients with sporadic amyotrohic lateral sclerosis. PLoS One. 2013;8(7):e68106.

144. Daoud H, Valdmanis PN, Kabashi E, Dion P, Dupré N, Camu W, et al. Contribution of TARDBP mutations to sporadic amyotrophic lateral sclerosis. J Med Genet. 2009;46(2):112-4.

145. Xu GR, Hu W, Zhan LL, Wang C, Xu LQ, Lin MT, et al. High frequency of the TARDBP p.M337V mutation among south-eastern Chinese patients with familial amyotrophic lateral sclerosis. BMC Neurology. 2018;18(1).

146. Khani M, Alavi A, Shamshiri H, Zamani B, Hassanpour H, Kazemi MH, et al. Mutation screening of SLC52A3, C19orf12, and TARDBP in Iranian ALS patients. Neurobiol Aging. 2019;75:225.e9-.e14.

147. Wang F, Fu S, Lei J, Wu H, Shi S, Chen K, et al. Identification of novel FUS and TARDBP gene mutations in Chinese amyotrophic lateral sclerosis patients with HRM analysis. Aging (Albany NY). 2020;12(22):22859-68.

148. Gromicho M, Coutinho AM, Pronto-Laborinho AC, Raposeiro R, Tavares J, Antunes D, et al. Targeted next-generation sequencing study in familial ALS-FTD Portuguese patients negative for C9orf72 HRE. J Neurol. 2020;267(12):3578-92.

149. Feng F, Wang H, Liu J, Wang Z, Xu B, Zhao K, et al. Genetic and clinical features of Chinese sporadic amyotrophic lateral sclerosis patients with TARDBP mutations. Brain Behav. 2021;11(8):e2312.

150. Nunes Gonçalves JP, Leoni TB, Martins MP, Peluzzo TM, Dourado MET, Jr., Saute JAM, et al. Genetic epidemiology of familial ALS in Brazil. Neurobiol Aging. 2021;102:227.e1-.e4.

151. Li J, Liu Q, Sun X, Zhang K, Liu S, Wang Z, et al. Genotype-phenotype association of TARDBP mutations in Chinese patients with amyotrophic lateral sclerosis: a single-center study and systematic review of published literature. J Neurol. 2022.

152. Corrado L, Mazzini L, Oggioni GD, Luciano B, Godi M, Brusco A, et al. ATXN-2 CAG repeat expansions are interrupted in ALS patients. Hum Genet. 2011;130(4):575-80.

153. Daoud H, Belzil V, Martins S, Sabbagh M, Provencher P, Lacomblez L, et al. Association of long ATXN2 CAG repeat sizes with increased risk of amyotrophic lateral sclerosis. Arch Neurol. 2011;68(6):739-42.

154. Chen Y, Huang R, Yang Y, Chen K, Song W, Pan P, et al. Ataxin-2 intermediate-length polyglutamine: a possible risk factor for Chinese patients with amyotrophic lateral sclerosis. Neurobiol Aging. 2011;32(10):1925.e1-5.

155. Ross OA, Rutherford NJ, Baker M, Soto-Ortolaza AI, Carrasquillo MM, DeJesus-Hernandez M, et al. Ataxin-2 repeat-length variation and neurodegeneration. Hum Mol Genet. 2011;20(16):3207-12.

156. Lahut S, Ömür Ö, Uyan Ö, Ağım ZS, Özoğuz A, Parman Y, et al. ATXN2 and its neighbouring gene SH2B3 are associated with increased ALS risk in the Turkish population. PLoS One. 2012;7(8):e42956.

157. Conforti FL, Spataro R, Sproviero W, Mazzei R, Cavalcanti F, Condino F, et al. Ataxin-1 and ataxin-2 intermediate-length PolyQ expansions in amyotrophic lateral sclerosis. Neurology. 2012;79(24):2315-20.

158. Van Langenhove T, van der Zee J, Engelborghs S, Vandenberghe R, Santens P, Van den Broeck M, et al. Ataxin-2 polyQ expansions in FTLD-ALS spectrum disorders in Flanders-Belgian cohorts. Neurobiol Aging. 2012;33(5):1004.e17-20.

159. Liu X, Lu M, Tang L, Zhang N, Chui D, Fan D. ATXN2 CAG repeat expansions increase the risk for Chinese patients with amyotrophic lateral sclerosis. Neurobiol Aging. 2013;34(9):2236.e5-8.

160. Lattante S, Millecamps S, Stevanin G, Rivaud-Péchoux S, Moigneu C, Camuzat A, et al. Contribution of ATXN2 intermediary polyQ expansions in a spectrum of neurodegenerative disorders. Neurology. 2014;83(11):990-5.

161. Lu HP, Gan SR, Chen S, Li HF, Liu ZJ, Ni W, et al. Intermediate-length polyglutamine in ATXN2 is a possible risk factor among Eastern Chinese patients with amyotrophic lateral sclerosis. Neurobiol Aging. 2015;36(3):1603.e11-4.

162. Borghero G, Pugliatti M, Marrosu F, Marrosu MG, Murru MR, Floris G, et al. ATXN2 is a modifier of phenotype in ALS patients of Sardinian ancestry. Neurobiol Aging. 2015;36(10):2906.e1-5.

163. Chiò A, Mora G, Sabatelli M, Caponnetto C, Lunetta C, Traynor BJ, et al. ATNX2 is not a regulatory gene in Italian amyotrophic lateral sclerosis patients with C9ORF72 GGGGCC expansion. Neurobiol Aging. 2016;39:218.e5-8.

164. Kim YE, Oh KW, Noh MY, Park J, Kim HJ, Park JE, et al. Analysis of ATXN2 trinucleotide repeats in Korean patients with amyotrophic lateral sclerosis. Neurobiol Aging. 2018;67:201.e5-.e8.

165. Tavares de Andrade HM, Cintra VP, de Albuquerque M, Piccinin CC, Bonadia LC, Duarte Couteiro RE, et al. Intermediate-length CAG repeat in ATXN2 is associated with increased risk for amyotrophic lateral sclerosis in Brazilian patients. Neurobiol Aging. 2018;69:292.e15-.e18.

166. Liu Z, Yuan Y, Wang M, Ni J, Li W, Huang L, et al. Mutation spectrum of amyotrophic lateral sclerosis in Central South China. Neurobiol Aging. 2021;107:181-8.

167. Borg R, Farrugia Wismayer M, Bonavia K, Farrugia Wismayer A, Vella M, van Vugt J, et al. Genetic analysis of ALS cases in the isolated island population of Malta. Eur J Hum Genet. 2021;29(4):604-14.

168. Chiò A, Borghero G, Restagno G, Mora G, Drepper C, Traynor BJ, et al. Clinical characteristics of patients with familial amyotrophic lateral sclerosis carrying the pathogenic GGGGCC hexanucleotide repeat expansion of C9ORF72. Brain. 2012;135(Pt 3):784-93.

169. Majounie E, Renton AE, Mok K, Dopper EGP, Waite A, Rollinson S, et al. Frequency of the C9orf72 hexanucleotide repeat expansion in patients with amyotrophic lateral sclerosis and frontotemporal dementia: A cross-sectional study. The Lancet Neurology. 2012;11(4):323-30.

170. Ishiura H, Takahashi Y, Mitsui J, Yoshida S, Kihira T, Kokubo Y, et al. C9ORF72 repeat expansion in amyotrophic lateral sclerosis in the Kii peninsula of Japan. Archives of Neurology. 2012;69(9):1154-8.

171. Ogaki K, Li Y, Atsuta N, Tomiyama H, Funayama M, Watanabe H, et al. Analysis of C9orf72 repeat expansion in 563 Japanese patients with amyotrophic lateral sclerosis. Neurobiol Aging. 2012;33(10):2527.e11-6.

172. Ratti A, Corrado L, Castellotti B, Del Bo R, Fogh I, Cereda C, et al. C9ORF72 repeat expansion in a large Italian ALS cohort: evidence of a founder effect. Neurobiol Aging. 2012;33(10):2528.e7-14.

173. Sabatelli M, Conforti FL, Zollino M, Mora G, Monsurrò MR, Volanti P, et al. C9ORF72 hexanucleotide repeat expansions in the Italian sporadic ALS population. Neurobiol Aging. 2012;33(8):1848.e15-20.

174. Stewart H, Rutherford NJ, Briemberg H, Krieger C, Cashman N, Fabros M, et al. Clinical and pathological features of amyotrophic lateral sclerosis caused by mutation in the C9ORF72 gene on chromosome 9p. Acta Neuropathologica. 2012;123(3):409-17.

175. Tsai CP, Soong BW, Tu PH, Lin KP, Fuh JL, Tsai PC, et al. A hexanucleotide repeat expansion in C9ORF72 causes familial and sporadic ALS in Taiwan. Neurobiol Aging. 2012;33(9):2232.e11-.e18.

176. van Rheenen W, van Blitterswijk M, Huisman MH, Vlam L, van Doormaal PT, Seelen M, et al. Hexanucleotide repeat expansions in C9ORF72 in the spectrum of motor neuron diseases. Neurology. 2012;79(9):878-82.

177. Xi Z, Zinman L, Grinberg Y, Moreno D, Sato C, Bilbao JM, et al. Investigation of C9orf72 in 4 neurodegenerative disorders. Archives of Neurology. 2012;69(12):1583-90.

178. Debray S, Race V, Crabbé V, Herdewyn S, Matthijs G, Goris A, et al. Frequency of C9orf72 repeat expansions in amyotrophic lateral sclerosis: a Belgian cohort study. Neurobiol Aging. 2013;34(12):2890.e7-.e12.

179. Jang JH, Kwon MJ, Choi WJ, Oh KW, Koh SH, Ki CS, et al. Analysis of the C9orf72 hexanucleotide repeat expansion in Korean patients with familial and sporadic amyotrophic lateral sclerosis. Neurobiol Aging. 2013;34(4):1311.e7-9.

180. Smith BN, Newhouse S, Shatunov A, Vance C, Topp S, Johnson L, et al. The C9ORF72 expansion mutation is a common cause of ALS+/-FTD in Europe and has a single founder. Eur J Hum Genet. 2013;21(1):102-8.

181. Jiao B, Tang B, Liu X, Yan X, Zhou L, Yang Y, et al. Identification of C9orf72 repeat expansions in patients with amyotrophic lateral sclerosis and frontotemporal dementia in mainland China. Neurobiol Aging. 2014;35(4):936.e19-22.

182. He J, Tang L, Benyamin B, Shah S, Hemani G, Liu R, et al. C9orf72 hexanucleotide repeat expansions in Chinese sporadic amyotrophic lateral sclerosis. Neurobiol Aging. 2015;36(9):2660.e1-8.

183. Van Es MA, Seelen M, Van Rheenen W, Van Doormaal P, Van Der Kooi A, De Visser M, et al. Large scale genetic screening in sporadic ALS identifies modifiers in C9orf72 repeat carriers. Amyotrophic Lateral Sclerosis and Frontotemporal Degeneration. 2015;16:32.

184. Vrabec K, Koritnik B, Leonardis L, Dolenc-Grošelj L, Zidar J, Smith B, et al. Genetic analysis of amyotrophic lateral sclerosis in the Slovenian population. Neurobiol Aging. 2015;36(3):1601.e17-20.

185. Itzcovich T, Xi Z, Martinetto H, Chrem-Méndez P, Russo MJ, de Ambrosi B, et al. Analysis of C9orf72 in patients with frontotemporal dementia and amyotrophic lateral sclerosis from Argentina. Neurobiology of Aging. 2016;40:192.e13-.e15.

186. Gibson SB, Downie JM, Tsetsou S, Feusier JE, Figueroa KP, Bromberg MB, et al. The evolving genetic risk for sporadic ALS. Neurology. 2017;89(3):226-33.

187. Narain P, Gomes J, Bhatia R, Singh I, Vivekanandan P. C9orf72 hexanucleotide repeat expansions and Ataxin 2 intermediate length repeat expansions in Indian patients with amyotrophic lateral sclerosis. Neurobiol Aging. 2017;56:211.e9-.e14.

188. Vats A, Gourie-Devi M, Suroliya V, Verma S, Faruq M, Sharma A, et al. Analysis of C9orf72 repeat expansion in amyotrophic lateral sclerosis patients from North India. J Neurol Sci. 2017;373:55-7.

189. Kartanou C, Koutsis G, Breza M, Rentzos M, Zouvelou V, Papageorgiou SG, et al. The C9ORF72 repeat expansion in Greek patients with neurodegenerative disorders. European Journal of Neurology. 2018;25:265.

190. Cintra VP, Bonadia LC, Andrade HMT, de Albuquerque M, Eusébio MF, de Oliveira DS, et al. The frequency of the C9orf72 expansion in a Brazilian population. Neurobiol Aging. 2018;66:179.e1-.e4.

191. Tripolszki K, Gampawar P, Schmidt H, Nagy ZF, Nagy D, Klivényi P, et al. Comprehensive genetic analysis of a Hungarian amyotrophic lateral sclerosis cohort. Frontiers in Genetics. 2019;10(JUL).

192. Trojsi F, Siciliano M, Femiano C, Santangelo G, Lunetta C, Calvo A, et al. Comparative analysis of C9Orf72 and sporadic disease in a large multicenter ALS population: The effect of Male sex on survival of C9Orf72 positive patients. Frontiers in Neuroscience. 2019;13(MAY).

193. Roggenbuck J, Palettas M, Patel R, Quick A, Kolb S. Genetic testing for als: The incidence of pathogenic, likely pathogenic, and uncertain variants in a clinic-based population. Neurology. 2020;94(15).

194. Sokratous M, Lucia S, Bourinaris T, Marogianni C, Arnaoutoglou M, Patrikiou E, et al. Prevalence of C9orf72 hexanucleotide repeat expansion in Greek patients with sporadic ALS. Amyotroph Lateral Scler Frontotemporal Degener. 2020;21(5-6):470-2.

195. Belzil VV, Valdmanis PN, Dion PA, Daoud H, Kabashi E, Noreau A, et al. Mutations in FUS cause FALS and SALS in French and French Canadian populations. Neurology. 2009;73(15):1176-9.

196. Kwiatkowski TJ, Jr., Bosco DA, Leclerc AL, Tamrazian E, Vanderburg CR, Russ C, et al. Mutations in the FUS/TLS gene on chromosome 16 cause familial amyotrophic lateral sclerosis. Science. 2009;323(5918):1205-8.

197. Ticozzi N, Silani V, LeClerc AL, Keagle P, Gellera C, Ratti A, et al. Analysis of FUS gene mutation in familial amyotrophic lateral sclerosis within an Italian cohort. Neurology. 2009;73(15):1180-5.

198. Corrado L, Del Bo R, Castellotti B, Ratti A, Cereda C, Penco S, et al. Mutations of FUS gene in sporadic amyotrophic lateral sclerosis. J Med Genet. 2010;47(3):190-4.

199. Damme PV, Goris A, Race V, Hersmus N, Dubois B, Van Den Bosch L, et al. The occurrence of mutations in FUS in a Belgian cohort of patients with familial ALS. European Journal of Neurology. 2010;17(5):754-6.

200. DeJesus-Hernandez M, Kocerha J, Finch N, Crook R, Baker M, Desaro P, et al. De novo truncating FUS gene mutation as a cause of sporadic amyotrophic lateral sclerosis. Hum Mutat. 2010;31(5):E1377-89.

201. Hewitt C, Kirby J, Highley JR, Hartley JA, Hibberd R, Hollinger HC, et al. Novel FUS/TLS mutations and pathology in familial and sporadic amyotrophic lateral sclerosis. Arch Neurol. 2010;67(4):455-61.

202. Yan J, Deng HX, Siddique N, Fecto F, Chen W, Yang Y, et al. Frameshift and novel mutations in FUS in familial amyotrophic lateral sclerosis and ALS/dementia. Neurology. 2010;75(9):807-14.

203. Drepper C, Herrmann T, Wessig C, Beck M, Sendtner M. C-terminal FUS/TLS mutations in familial and sporadic ALS in Germany. Neurobiol Aging. 2011;32(3):548.e1-4.

204. Rutherford NJ, Finch NA, DeJesus-Hernandez M, Crook RJ, Lomen-Hoerth C, Wszolek ZK, et al. Pathogenicity of exonic indels in fused in sarcoma in amyotrophic lateral sclerosis. Neurobiol Aging. 2012;33(2):424.e23-4.

205. Sproviero W, La Bella V, Mazzei R, Valentino P, Rodolico C, Simone IL, et al. FUS mutations in sporadic amyotrophic lateral sclerosis: clinical and genetic analysis. Neurobiol Aging. 2012;33(4):837.e1-5.

206. Zou ZY, Cui LY, Sun Q, Li XG, Liu MS, Xu Y, et al. De novo FUS gene mutations are associated with juvenile-onset sporadic amyotrophic lateral sclerosis in China. Neurobiol Aging. 2013;34(4):1312.e1-8.

207. Sabatelli M, Moncada A, Conte A, Lattante S, Marangi G, Luigetti M, et al. Mutations in the 3' untranslated region of FUS causing FUS overexpression are associated with amyotrophic lateral sclerosis. Hum Mol Genet. 2013;22(23):4748-55.

208. Tarlarini C, Lunetta C, Mosca L, Avemaria F, Riva N, Mantero V, et al. Novel FUS mutations identified through molecular screening in a large cohort of familial and sporadic amyotrophic lateral sclerosis. Eur J Neurol. 2015;22(11):1474-81.

209. Akiyama T, Warita H, Kato M, Nishiyama A, Izumi R, Ikeda C, et al. Genotype-phenotype relationships in familial amyotrophic lateral sclerosis with FUS/TLS mutations in Japan. Muscle Nerve. 2016;54(3):398-404.

210. Daoud H, Suhail H, Szuto A, Camu W, Salachas F, Meininger V, et al. UBQLN2 mutations are rare in French and French-Canadian amyotrophic lateral sclerosis. Neurobiol Aging. 2012;33(9):2230.e1-.e5.

211. van Doormaal PT, van Rheenen W, van Blitterswijk M, Schellevis RD, Schelhaas HJ, de Visser M, et al. UBQLN2 in familial amyotrophic lateral sclerosis in The Netherlands. Neurobiol Aging. 2012;33(9):2233.e7-.e8.

212. Millecamps S, Corcia P, Cazeneuve C, Boillée S, Seilhean D, Danel-Brunaud V, et al. Mutations in UBQLN2 are rare in French amyotrophic lateral sclerosis. Neurobiol Aging. 2012;33(4):839.e1-3.

213. Dillen L, Van Langenhove T, Engelborghs S, Vandenbulcke M, Sarafov S, Tournev I, et al. Explorative genetic study of UBQLN2 and PFN1 in an extended Flanders-Belgian cohort of frontotemporal lobar degeneration patients. Neurobiol Aging. 2013;34(6):1711.e1-5.

214. Gellera C, Tiloca C, Del Bo R, Corrado L, Pensato V, Agostini J, et al. Ubiquilin 2 mutations in Italian patients with amyotrophic lateral sclerosis and frontotemporal dementia. J Neurol Neurosurg Psychiatry. 2013;84(2):183-7.

215. Lattante S, Le Ber I, Camuzat A, Pariente J, Brice A, Kabashi E. Screening UBQLN-2 in French frontotemporal lobar degeneration and frontotemporal lobar degeneration-amyotrophic lateral sclerosis patients. Neurobiol Aging. 2013;34(8):2078.e5-6.

216. Kim HJ, Kwon MJ, Choi WJ, Oh KW, Oh SI, Ki CS, et al. Mutations in UBQLN2 and SIGMAR1 genes are rare in Korean patients with amyotrophic lateral sclerosis. Neurobiol Aging. 2014;35(8):1957.e7-8.

217. McLaughlin RL, Kenna KP, Vajda A, Byrne S, Bradley DG, Hardiman O. UBQLN2 mutations are not a frequent cause of amyotrophic lateral sclerosis in Ireland. Neurobiol Aging. 2014;35(1):267.e9-11.

218. Özoğuz A, Uyan Ö, Birdal G, Iskender C, Kartal E, Lahut S, et al. The distinct genetic pattern of ALS in Turkey and novel mutations. Neurobiol Aging. 2015;36(4):1764.e9-.e18.

219. Huang X, Shen S, Fan D. No Evidence for Pathogenic Role of UBQLN2 Mutations in Sporadic Amyotrophic Lateral Sclerosis in the Mainland Chinese Population. PLoS One. 2017;12(1):e0170943.

220. McCann EP, Williams KL, Fifita JA, Tarr IS, O'Connor J, Rowe DB, et al. The genotype-phenotype landscape of familial amyotrophic lateral sclerosis in Australia. Clin Genet. 2017;92(3):259-66.

221. Scotter EL, Smyth L, Bailey J, Wong CH, de Majo M, Vance CA, et al. C9ORF72 and UBQLN2 mutations are causes of amyotrophic lateral sclerosis in New Zealand: a genetic and pathologic study using banked human brain tissue. Neurobiol Aging. 2017;49:214.e1-.e5.

222. Johnson JO, Mandrioli J, Benatar M, Abramzon Y, Van Deerlin VM, Trojanowski JQ, et al. Exome sequencing reveals VCP mutations as a cause of familial ALS. Neuron. 2010;68(5):857-64.

223. Koppers M, van Blitterswijk MM, Vlam L, Rowicka PA, van Vught PW, Groen EJ, et al. VCP mutations in familial and sporadic amyotrophic lateral sclerosis. Neurobiol Aging. 2012;33(4):837.e7-13.

224. Miller JW, Smith BN, Topp SD, Al-Chalabi A, Shaw CE, Vance C. Mutation analysis of VCP in British familial and sporadic amyotrophic lateral sclerosis patients. Neurobiol Aging. 2012;33(11):2721.e1-2.

225. Abramzon Y, Johnson JO, Scholz SW, Taylor JP, Brunetti M, Calvo A, et al. Valosin-containing protein (VCP) mutations in sporadic amyotrophic lateral sclerosis. Neurobiol Aging. 2012;33(9):2231.e1-.e6.

226. Tiloca C, Ratti A, Pensato V, Castucci A, Sorarù G, Del Bo R, et al. Mutational analysis of VCP gene in familial amyotrophic lateral sclerosis. Neurobiol Aging. 2012;33(3):630.e1-2.

227. Williams KL, Solski JA, Nicholson GA, Blair IP. Mutation analysis of VCP in familial and sporadic amyotrophic lateral sclerosis. Neurobiol Aging. 2012;33(7):1488.e15-6.

228. Zou ZY, Liu MS, Li XG, Cui LY. Screening of VCP mutations in Chinese amyotrophic lateral sclerosis patients. Neurobiol Aging. 2013;34(5):1519.e3-4.

229. Hirano M, Nakamura Y, Saigoh K, Sakamoto H, Ueno S, Isono C, et al. VCP gene analyses in Japanese patients with sporadic amyotrophic lateral sclerosis identify a new mutation. Neurobiol Aging. 2015;36(3):1604.e1-6.

230. Naruse H, Ishiura H, Mitsui J, Date H, Takahashi Y, Matsukawa T, et al. Molecular epidemiological study of familial amyotrophic lateral sclerosis in Japanese population by whole-exome sequencing and identification of novel HNRNPA1 mutation. Neurobiol Aging. 2018;61:255.e9-.e16.
