# Supplementary material for: Risk factors of amyotrophic lateral sclerosis: a global meta-summary
Source: Front Neurosci. 2023 Apr 24;17:1177431. doi: 10.3389/fnins.2023.1177431 (PMC10165003; doi:10.3389/fnins.2023.1177431)

**Catalog**

| **Forest plot** |  |  |
| --- | --- | --- |
| 1.Pool analysis of environmental toxicity | Supp. Fig. 1 | Page 2 |
| 2.Pool analysis of lifestyle | Supp. Fig. 2 | Page 3 |
| 3.Pool analysis of pre-existing diseases/comorbidity and medical exposure | Supp. Fig. 3 | Page 4 |
| 4.Pool analysis of other non-genetic factors | Supp. Fig. 4 | Page 5 |
| 5. Pool analysis of frequency of causative/risk genes in ALS | Supp. Fig. 5 | Page 6 |
| 6. Pool analysis of frequency of causative/risk genes in SALS  7. Pool analysis of frequency of causative/risk genes in FALS  8. Pool analysis of frequency of causative/risk genes in European/Asian | Supp. Fig. 6  Supp. Fig. 7  Supp. Fig. 8 | Page 7  Page 8  Page 9 |
| **Publication bias** |  |  |
| 1.begg’s plot of environmental toxicity | Supp. Fig. 9 | page 10 |
| 2.begg’s plot of comorbidity and treatment | Supp. Fig. 10 | page 11-12 |
| 3.begg’s plot of lifestyle | Supp. Fig. 11 | page 13 |
| 4.begg’ plot of other non-genetic factors | Supp. Fig. 12 | page 14-15 |
| 5.begg’ plot of genes | Supp. Fig. 13 | page 16-17 |
| **Sensitivity analysis** |  |  |
| 1.Sensitivity analysis of environmental toxicity | Supp. Fig. 14 | Page 18 |
| 2.Sensitivity analysis of pre-existing disease/  comorbidity and medical exposure | Supp. Fig. 15 | Page 19 |
| 3.Sensitivity analysis of lifestyle | Supp. Fig. 16 | Page 20 |
| 4.Sensitivity analysis of other non-genetic factors | Supp. Fig. 17 | Page 21 |
| 5.Sensitivity analysis of genes | Supp. Fig. 18 | Page 22 |

**Supp. Fig. 1 Forest plot of environmental toxicity**

**
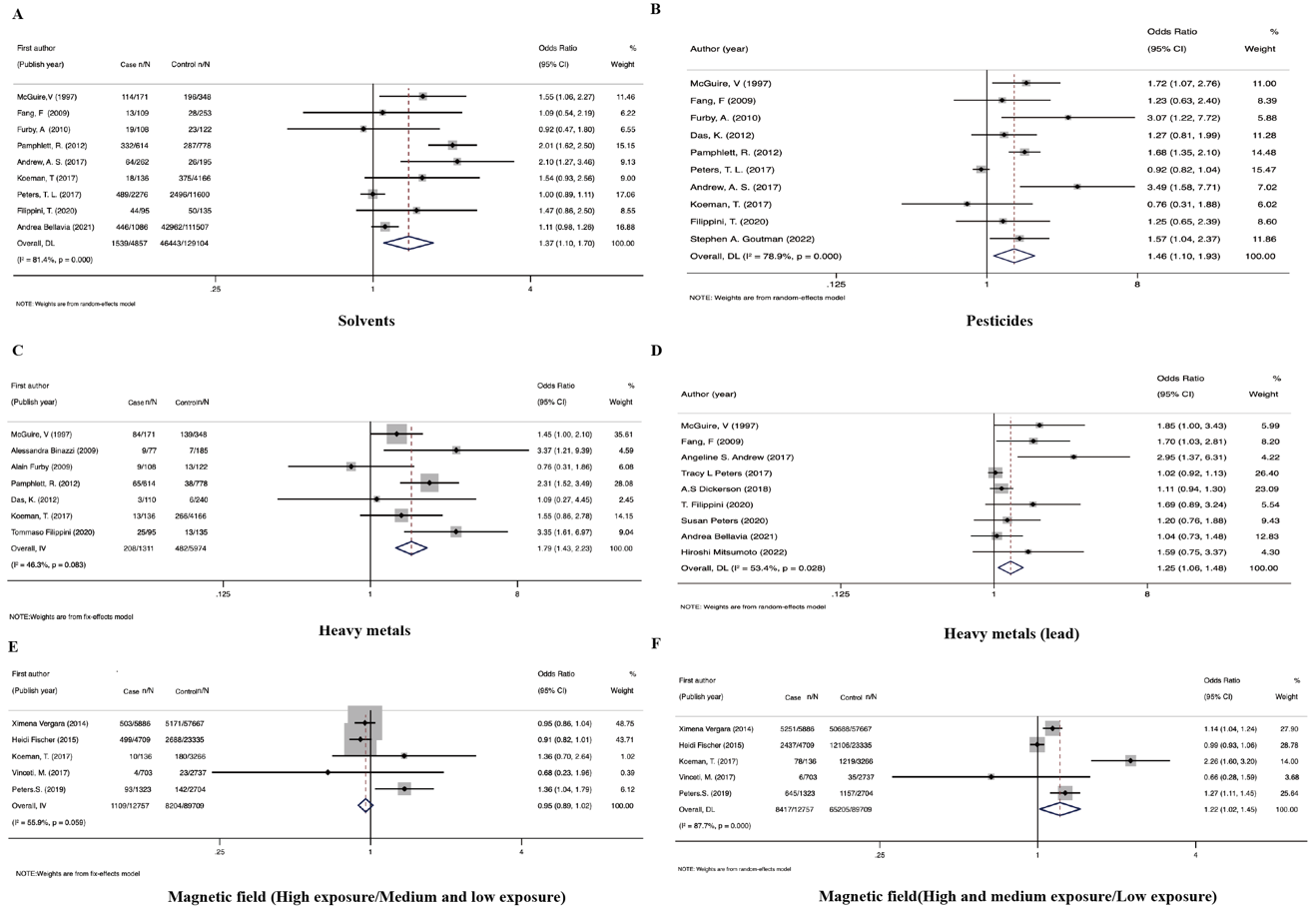
**

**Supp. Fig. 2 Forest plot of lifestyle**

**
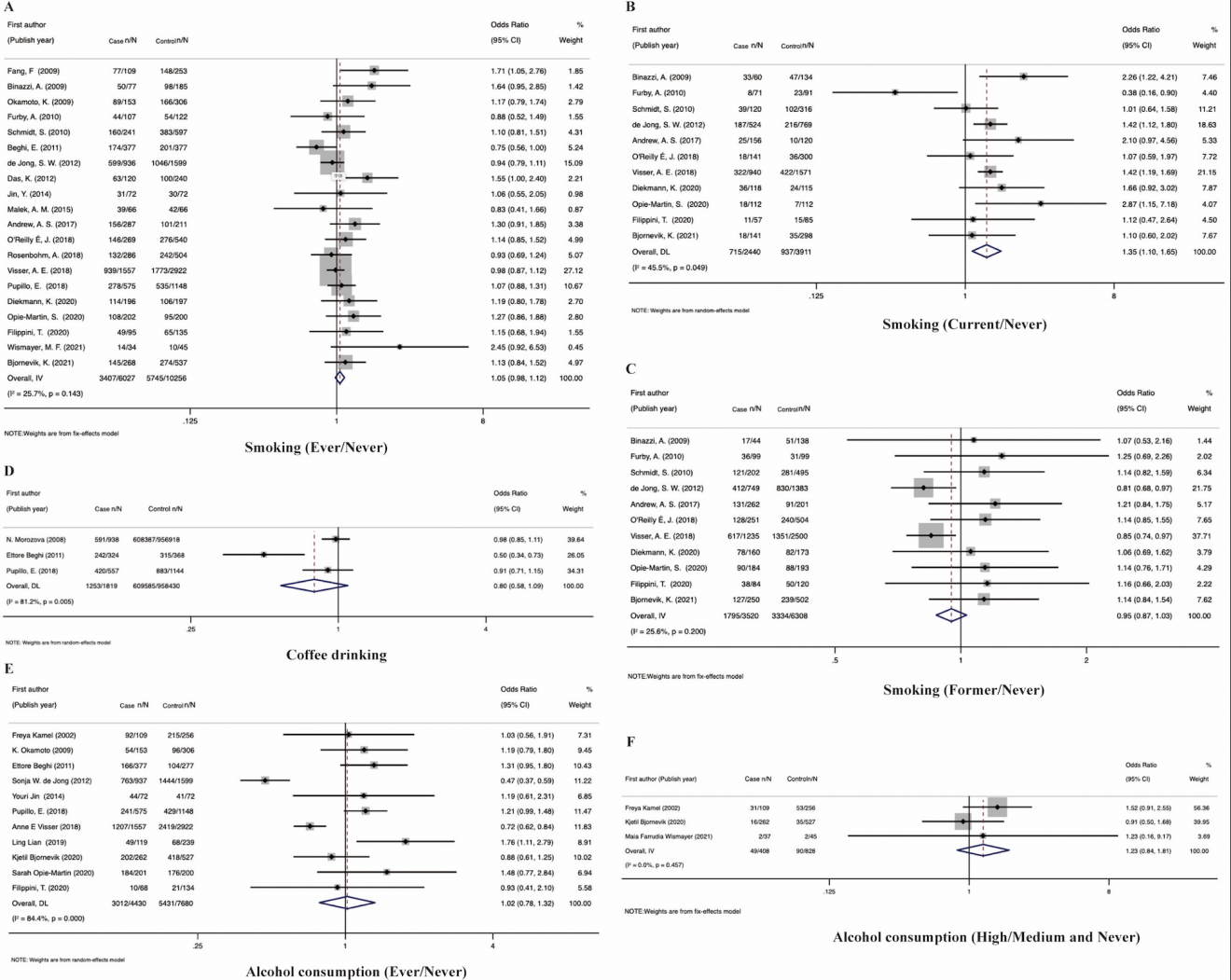
**

**Supp. Fig. 3 Forest plot of pre-existing diseases/comorbidity and medical exposure**

**
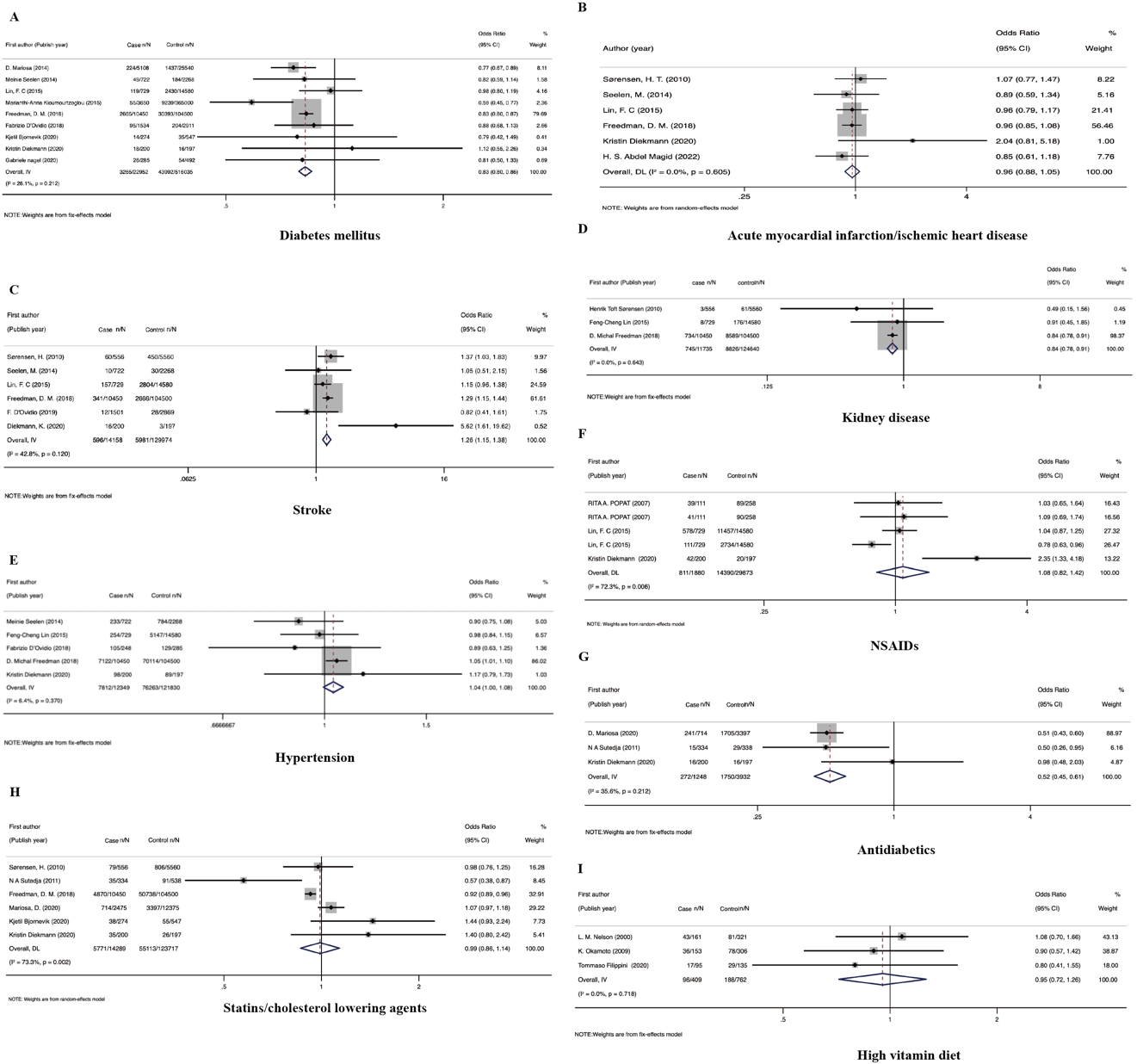
**

**Supp. Fig. 4 Forest plot of other non-genetic factors**

**
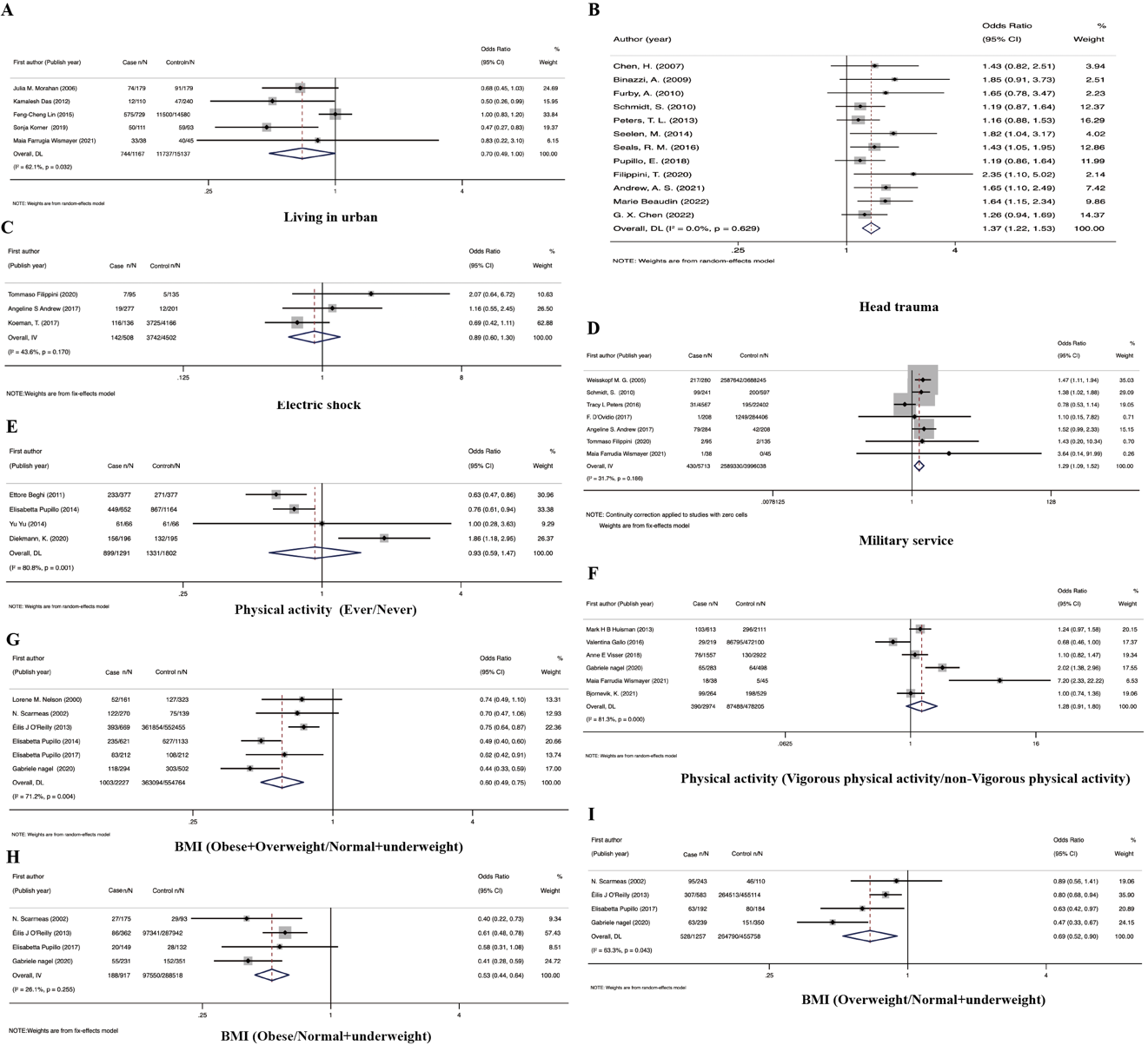
**

**Supp. Fig. 5 Frequency of causative/risk genes**

**
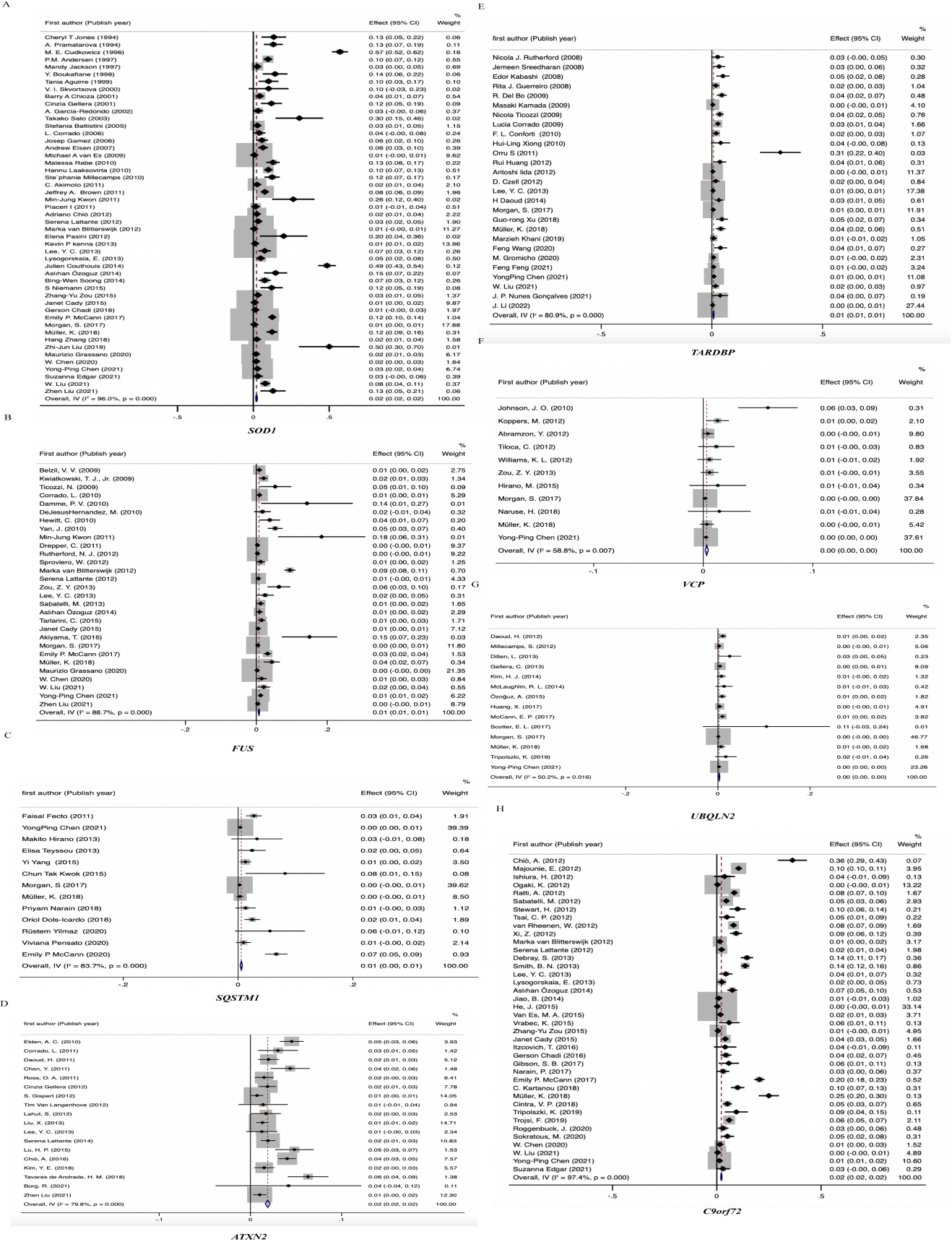
**

**Supp. Fig.6 Frequency of causative/risk genes in SALS**

**
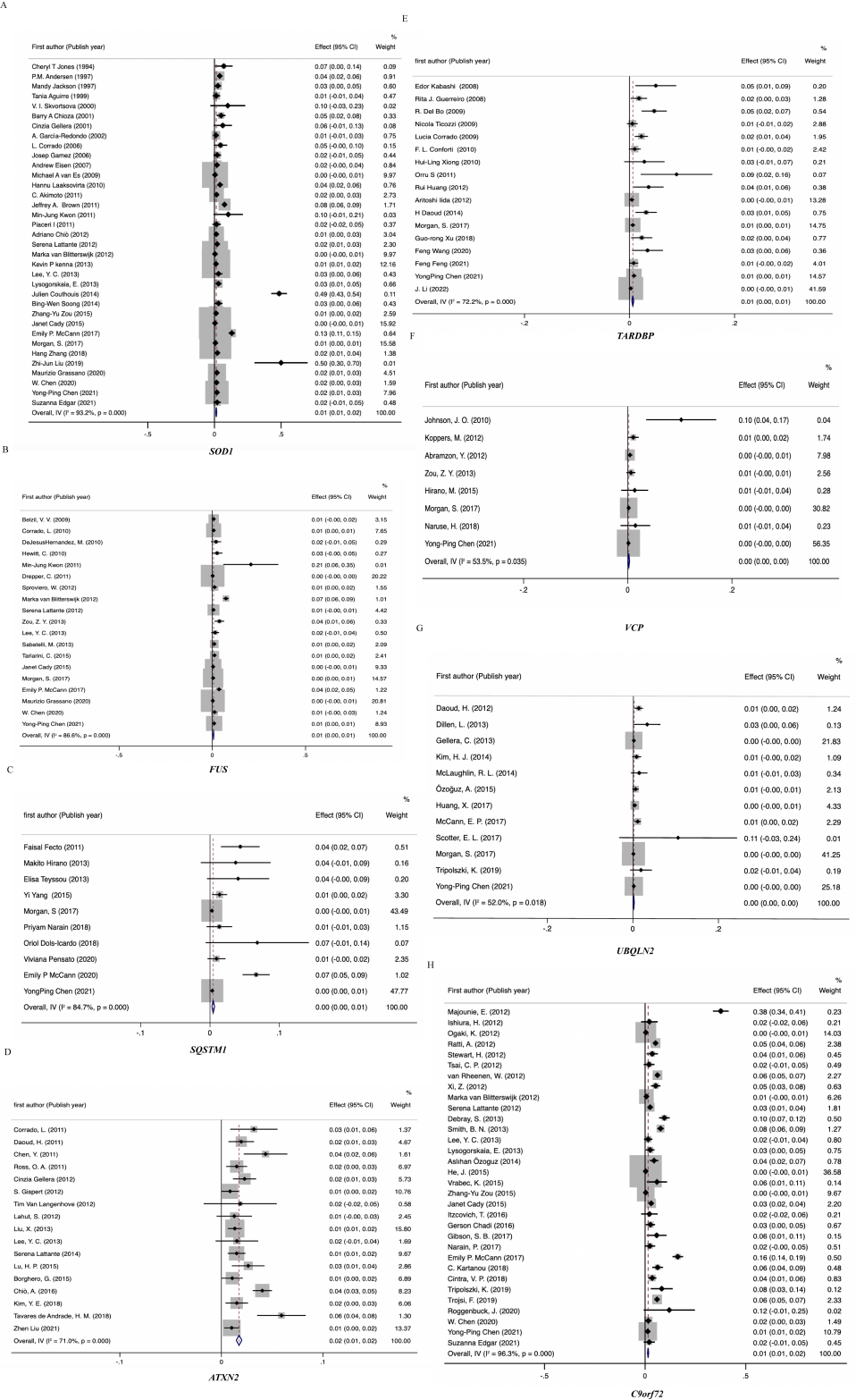
**

**Supp. Fig. 7 Frequency of causative/risk genes in FALS**

**
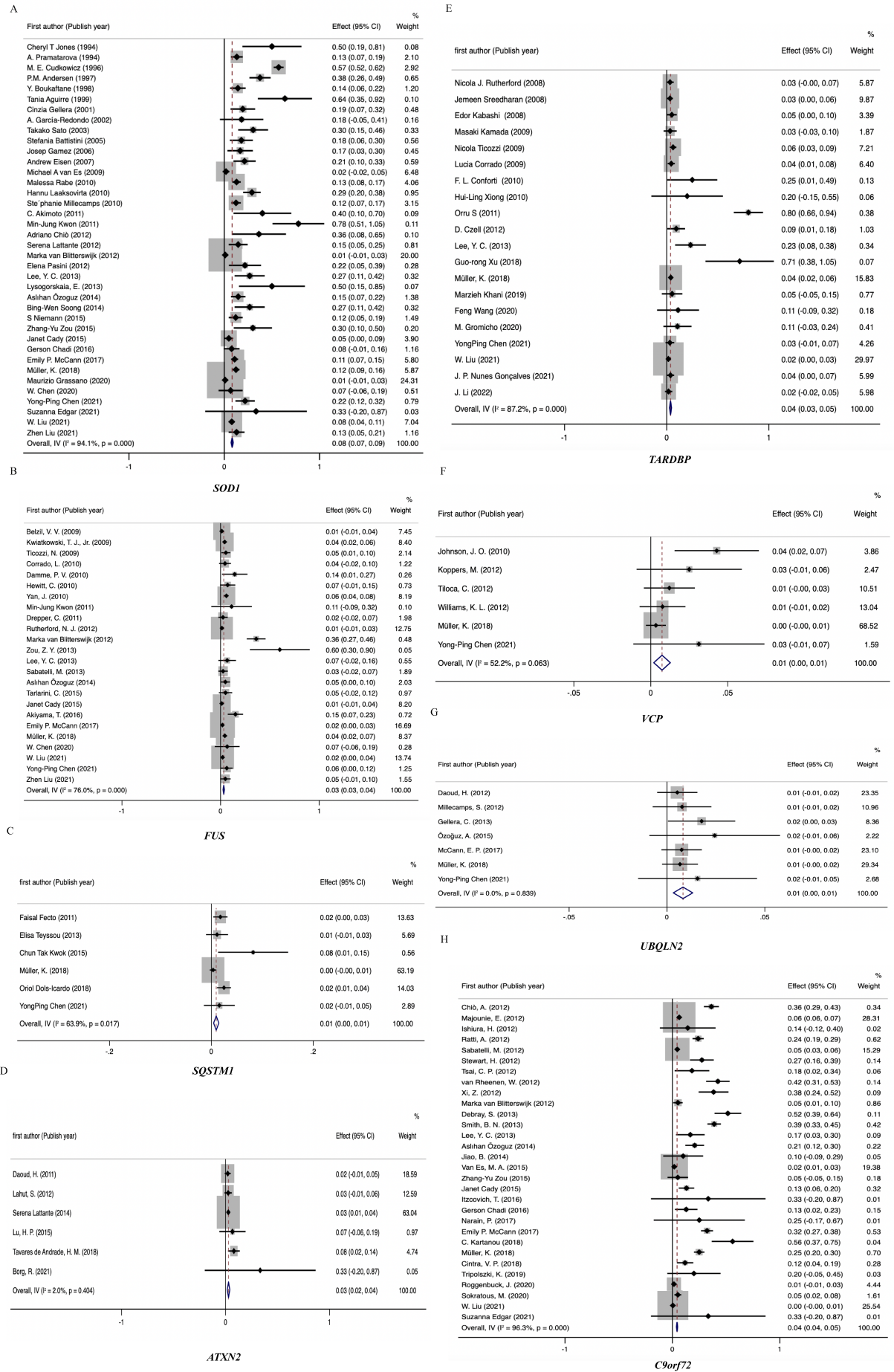
**

**Supp. Fig. 8 Frequency of causative/risk genes in European/Asian**

**
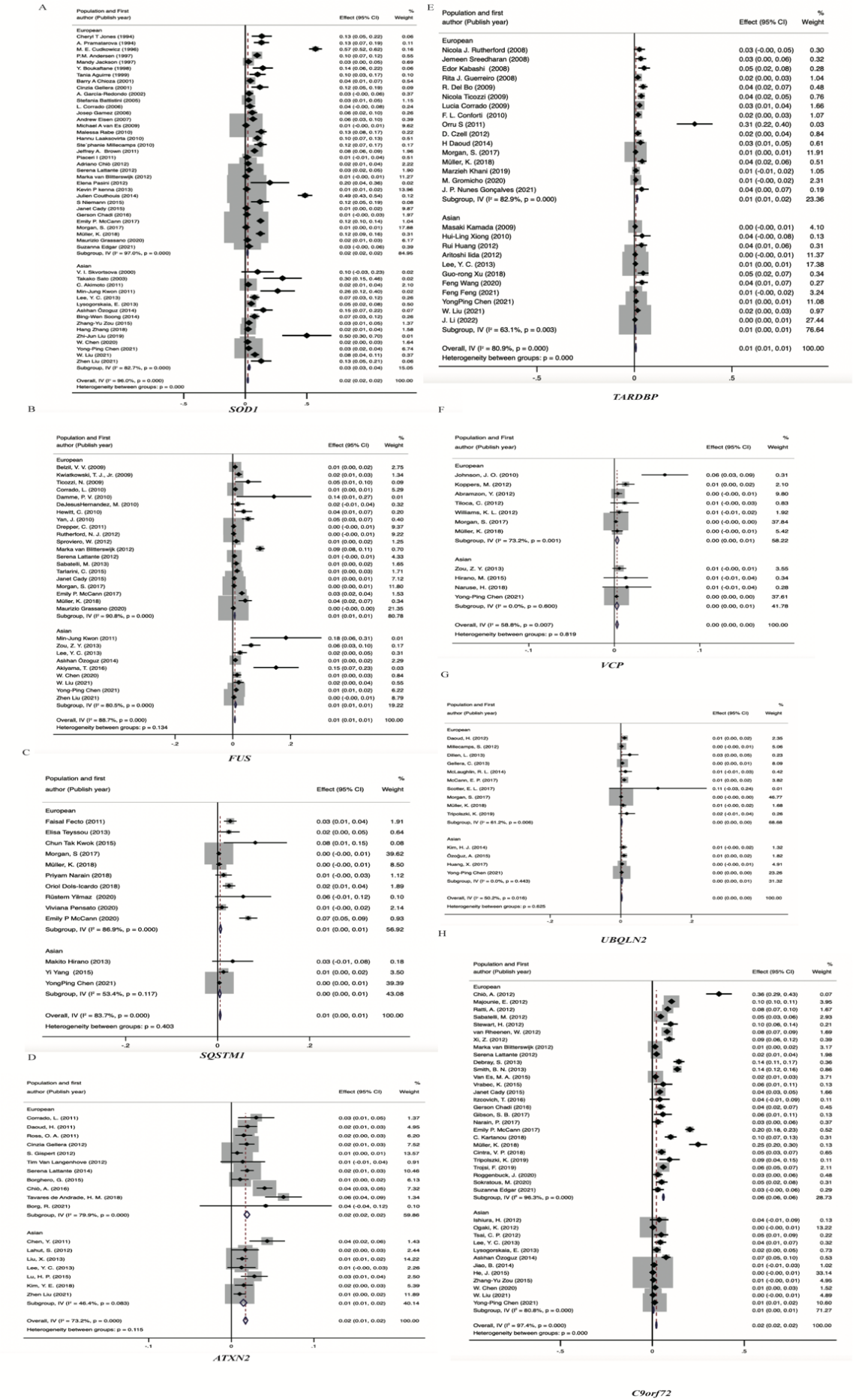
**

**Supp. Fig.9 Begg’s plot of environmental toxicity**

**
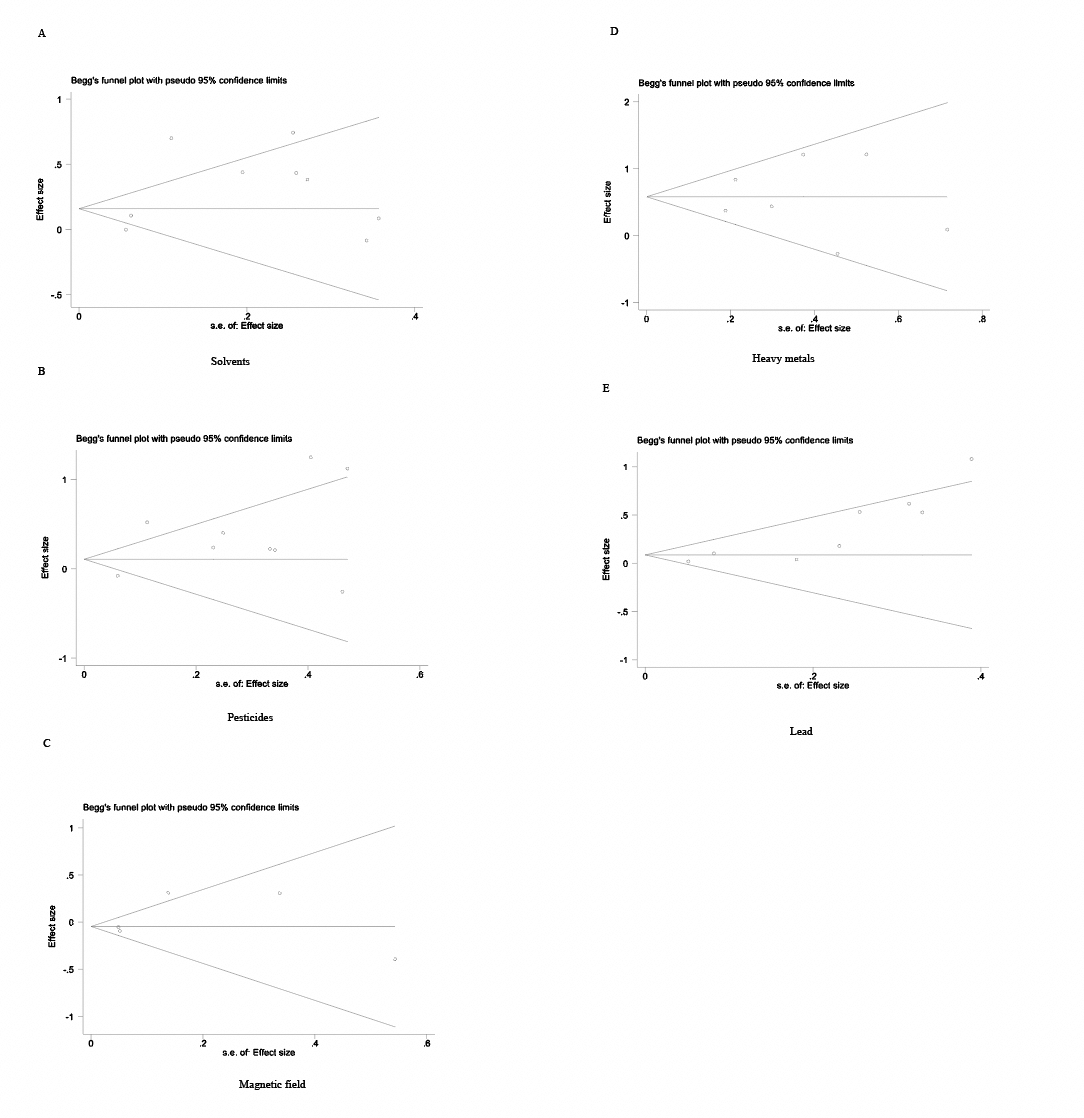
**

A: begg’s plot of solvents; Begg’s test: Pr ＞｜z｜= 0.835; Egger’s test: P ＞｜t｜= 0.977;

B: begg’s plot of pesticides; Begg’s test: Pr ＞｜z｜= 1.000; Egger’s test: P ＞｜t｜= 0.513;

C: begg’s plot of magnetic field; Begg’s test: Pr ＞｜z｜= 1.000; Egger’s test: P ＞｜t｜= 0.293;

D: begg’s plot of heavy metals; Begg’s test: Pr ＞｜z｜= 0.881; Egger’s test: P ＞｜t｜= 0.217;

E: begg’s plot of lead; Begg’s test: Pr ＞｜z｜= 0.013; Egger’s test: P ＞｜t｜= 0.142

**Supp. Fig.10 Begg’s plot of pre-existing diseases/comorbidity and medical exposure**


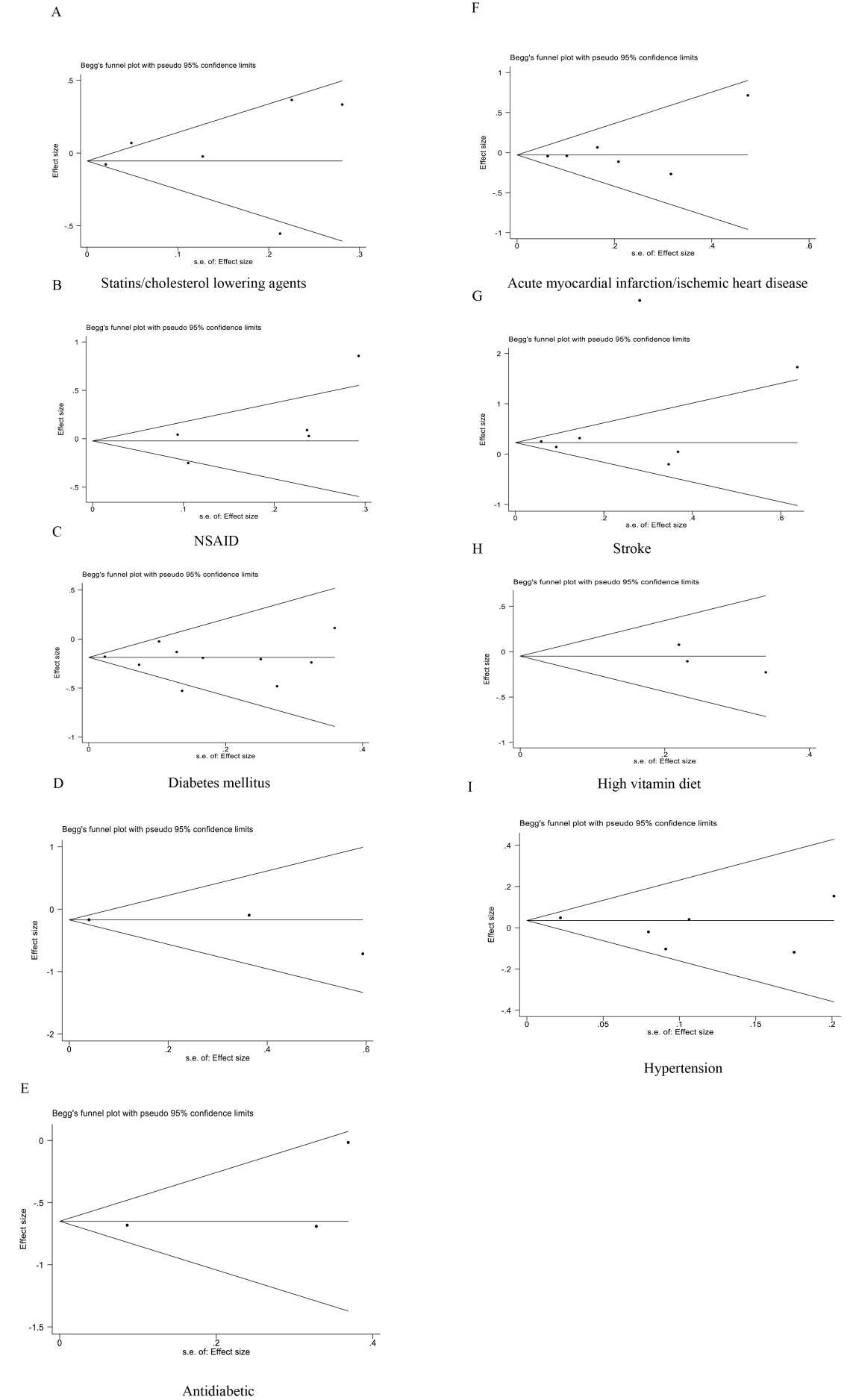


A: begg’s plot of statins/cholesterol lowering agents; Begg’s test: Pr ＞｜z｜= 0.573; Egger’s test: P ＞｜t｜= 0.204;

B: begg’s plot of NSAID; Begg’s test: Pr ＞｜z｜= 0.624; Egger’s test: P ＞｜t｜= 0.289;

C: begg’s plot of diabetes mellitus; Begg’s test: Pr ＞｜z｜= 0.677; Egger’s test: P ＞｜t｜= 0.001;

D: begg’s plot of kidney disease; Begg’s test: Pr ＞｜z｜= 0.117; Egger’s test: P ＞｜t｜= 0.170;

E: begg’s plot of antidiabetics; Begg’s test: Pr ＞｜z｜= 0.117; Egger’s test: P ＞｜t｜= 0.142；

F: begg’s plot of acute myocardial infarction/ischemic heart disease; Begg’s test: Pr ＞｜z｜= 0.327; Egger’s test: P ＞｜t｜= 0.172；

G: begg’s plot of stroke; Begg’s test: Pr ＞｜z｜= 0.851; Egger’s test: P ＞｜t｜= 0.136；

H: begg’s plot of high vitamin diet; Begg’s test: Pr ＞｜z｜= 0.117; Egger’s test: P ＞｜t｜= 0.457；

I: begg’s plot of hypertension; Begg’s test: Pr ＞｜z｜= 0.624; Egger’s test: P ＞｜t｜= 0.118；

**Supp. Fig.11 Begg’s plot of lifestyle**

**
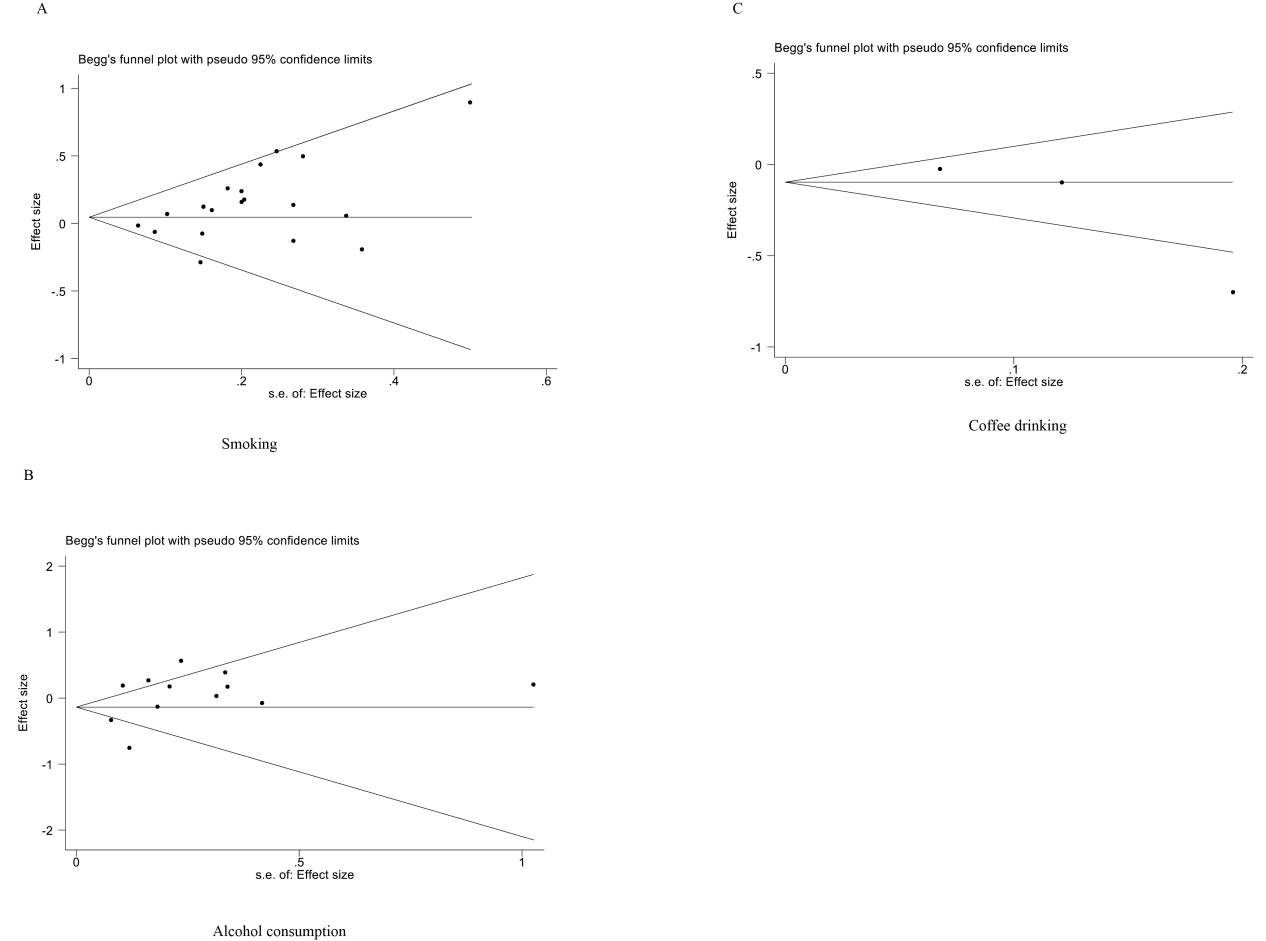
**

A: begg’s plot of smoking; Begg’s test: Pr ＞｜z｜= 0.032; Egger’s test: P ＞｜t｜= 0.102;

B: begg’s plot of alcohol consumption; Begg’s test: Pr ＞｜z｜= 0.891; Egger’s test: P ＞｜t｜= 0.087;

C: begg’s plot of coffee drinking; Begg’s test: Pr ＞｜z｜= 0.117; Egger’s test: P ＞｜t｜= 0.376;

**Supp. Fig.12 Begg’s plot of other non-genetic factors**


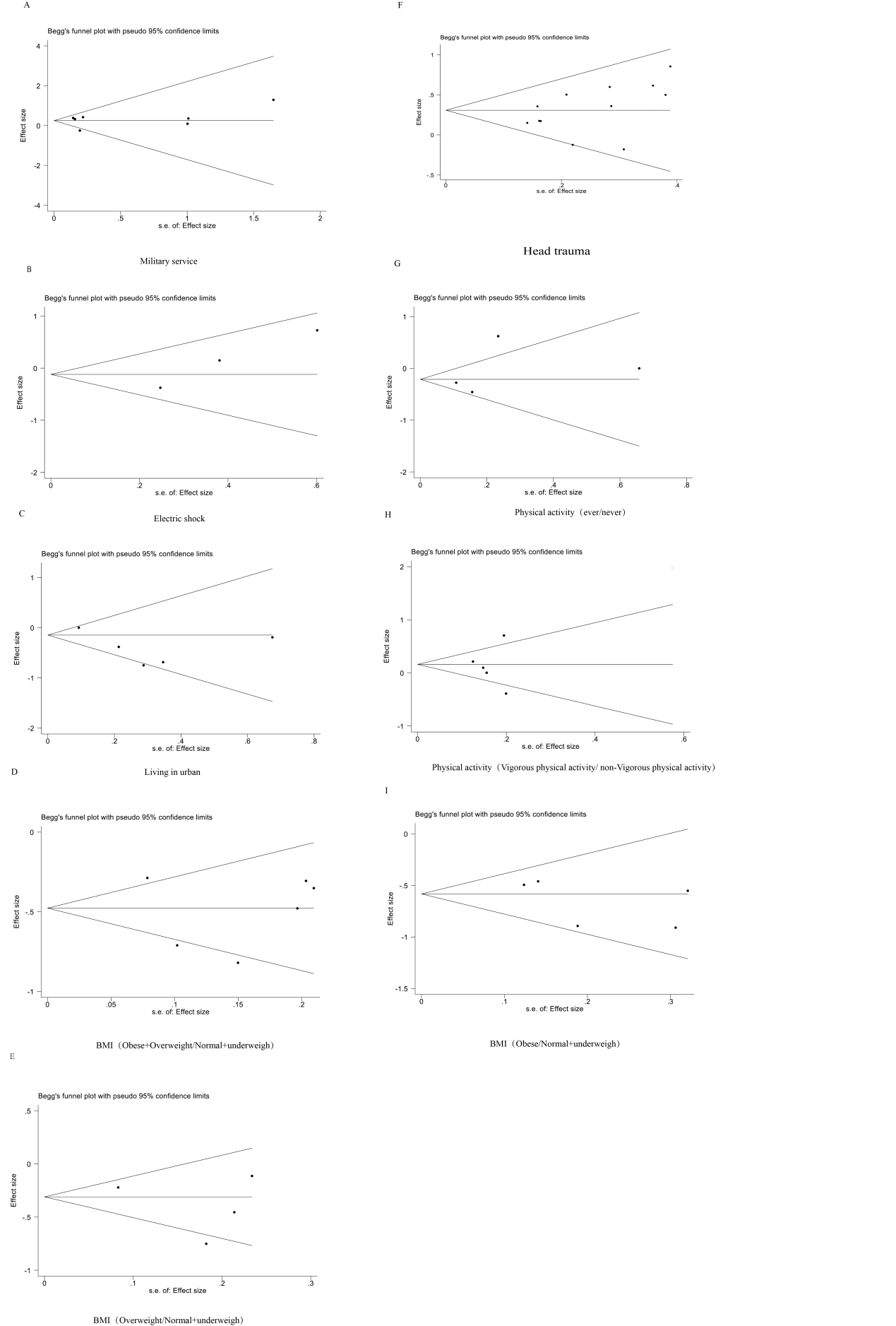


A: begg’s plot of military service; Begg’s test: Pr ＞｜z｜= 0.652; Egger’s test: P ＞｜t｜= 0.283;

B: begg’s plot of electric shock; Begg’s test: Pr ＞｜z｜= 0.117; Egger’s test: P ＞｜t｜= 0.074;

C: begg’s plot of living in urban; Begg’s test: Pr ＞｜z｜= 0.624; Egger’s test: P ＞｜t｜= 0.440;

D: begg’s plot of BMI（Obese+Overweight/Normal+underweight）; Begg’s test: Pr ＞｜z｜= 0.573; Egger’s test: P ＞｜t｜= 0.251;

E: begg’s plot of BMI（Overweight/Normal+underweight）; Begg’s test: Pr ＞｜z｜= 1.000; Egger’s test: P ＞｜t｜= 0.726

F: begg’s plot of head trauma; Begg’s test: Pr ＞｜z｜= 0.040; Egger’s test: P ＞｜t｜= 0.378；

G: begg’s plot of physical activity（ever/never）; Begg’s test: Pr ＞｜z｜= 0.497; Egger’s test: P ＞｜t｜= 0.374；

H: begg’s plot of physical activity（Vigorous physical activity/ non-Vigorous physical activity）t; Begg’s test: Pr ＞｜z｜= 0.851; Egger’s test: P ＞｜t｜= 0.485；

I: begg’s plot of BMI（Obese/Normal+underweight）; Begg’s test: Pr ＞｜z｜= 0.624; Egger’s test: P ＞｜t｜= 0.292.

**Supp. Fig.13 Begg’s plot of genes**


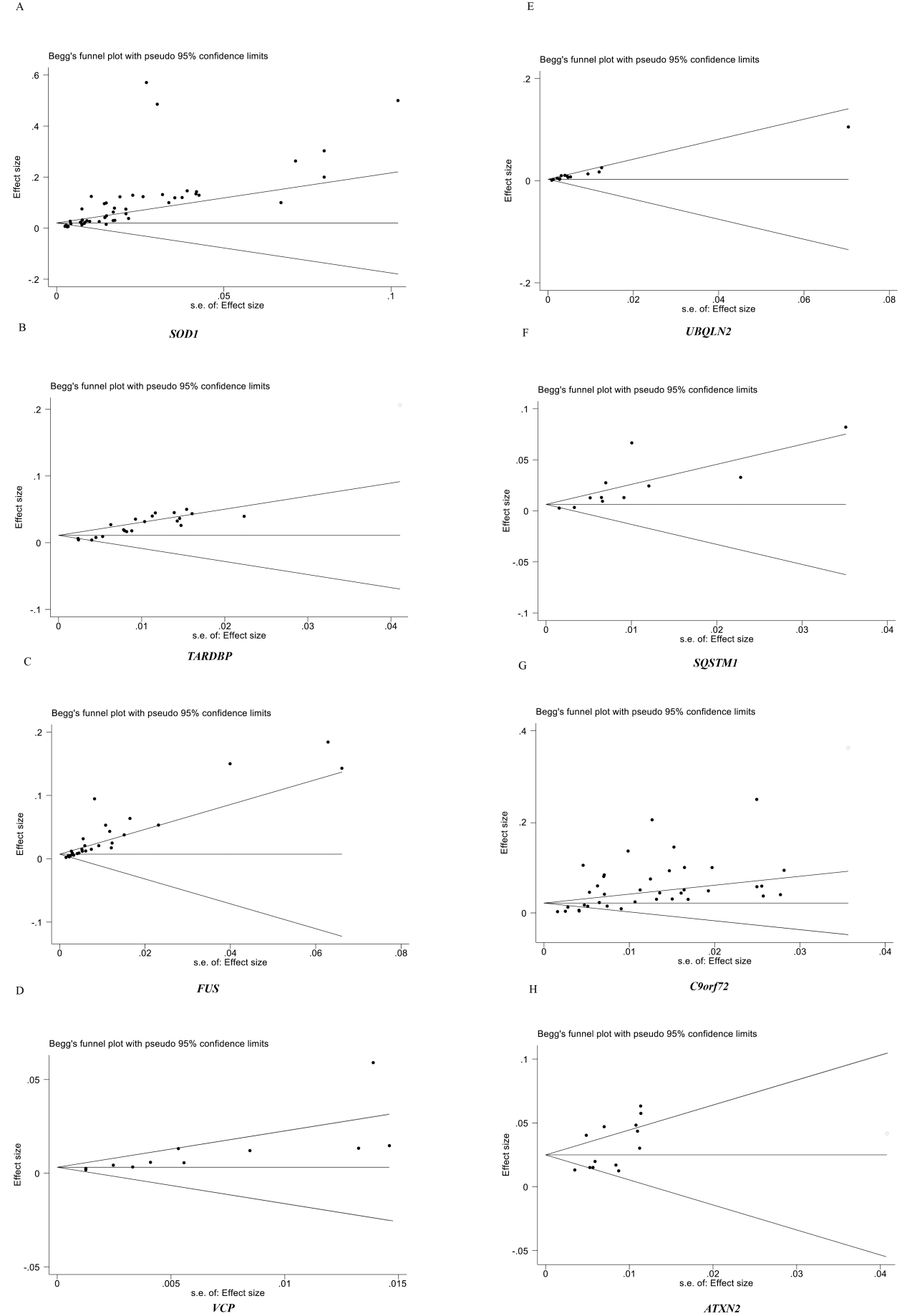


A: begg’s plot of *ATXN2*; Begg’s test: Pr ＞｜z｜= 0.037; Egger’s test: P ＞｜t｜= 0.263.

B: begg’s plot of *TARDBP*; Begg’s test: Pr ＞｜z｜= 0.000; Egger’s test: P ＞｜t｜= 0.287;

C: begg’s plot of *VCP*; Begg’s test: Pr ＞｜z｜= 0.000; Egger’s test: P ＞｜t｜= 0.356;

D: begg’s plot of *FUS*; Begg’s test: Pr ＞｜z｜= 0.000; Egger’s test: P ＞｜t｜= 0.287;

E: begg’s plot of *SQSTM1*; Begg’s test: Pr ＞｜z｜= 0.004; Egger’s test: P ＞｜t｜= 0.744;

F: begg’s plot of *SOD1*; Begg’s test: Pr ＞｜z｜= 0.000; Egger’s test: P ＞｜t｜= 0.394;

G: begg’s plot of *C9orf72;* Begg’s test: Pr ＞｜z｜= 0.022; Egger’s test: P ＞｜t｜= 0.691;

H: begg’s plot of *UBQLN2*; Begg’s test: Pr ＞｜z｜= 0.128; Egger’s test: P ＞｜t｜= 0.571.

**Supp. Fig.14 Sensitivity analysis of environmental coxticy**


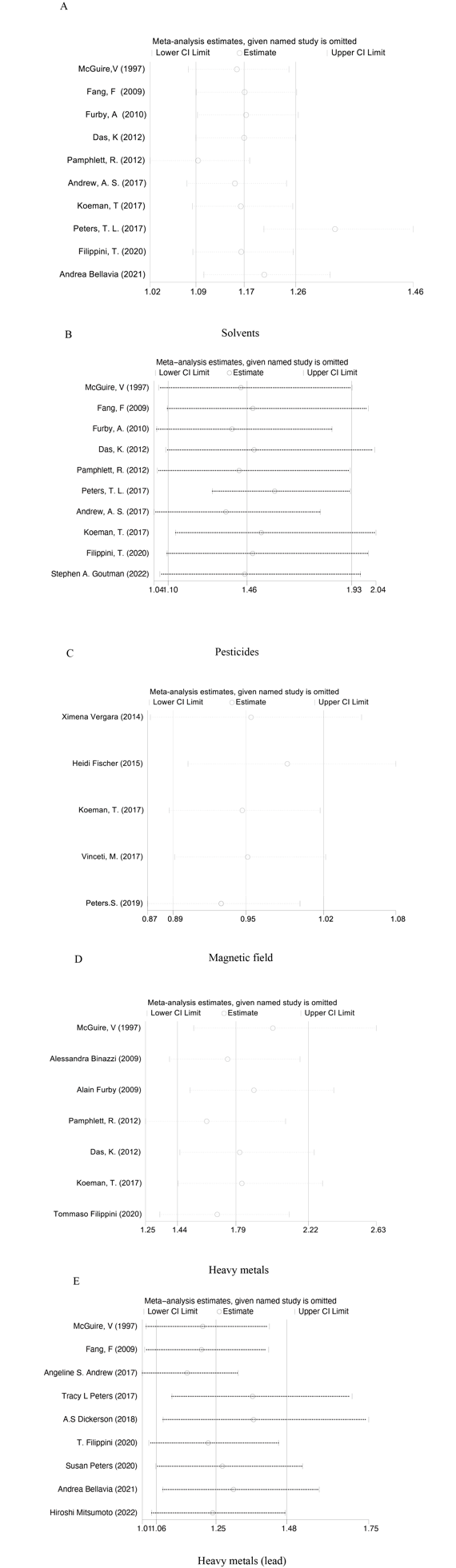


**Supp. Fig.15 Sensitivity analysis of pre-existing diseases/comorbidity and medical exposure**


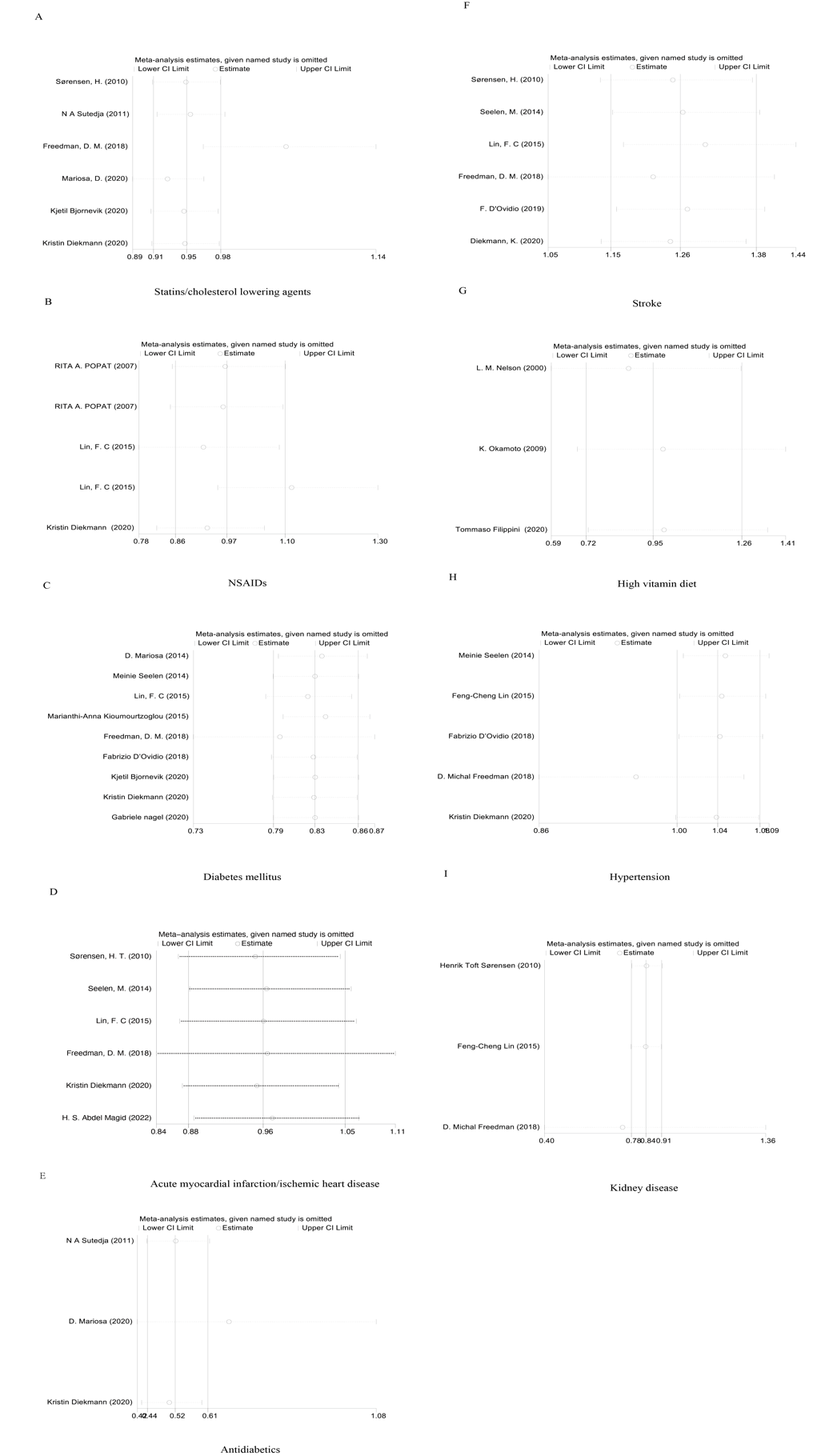


**Supp. Fig.16 Sensitivity analysis of lifestyle**


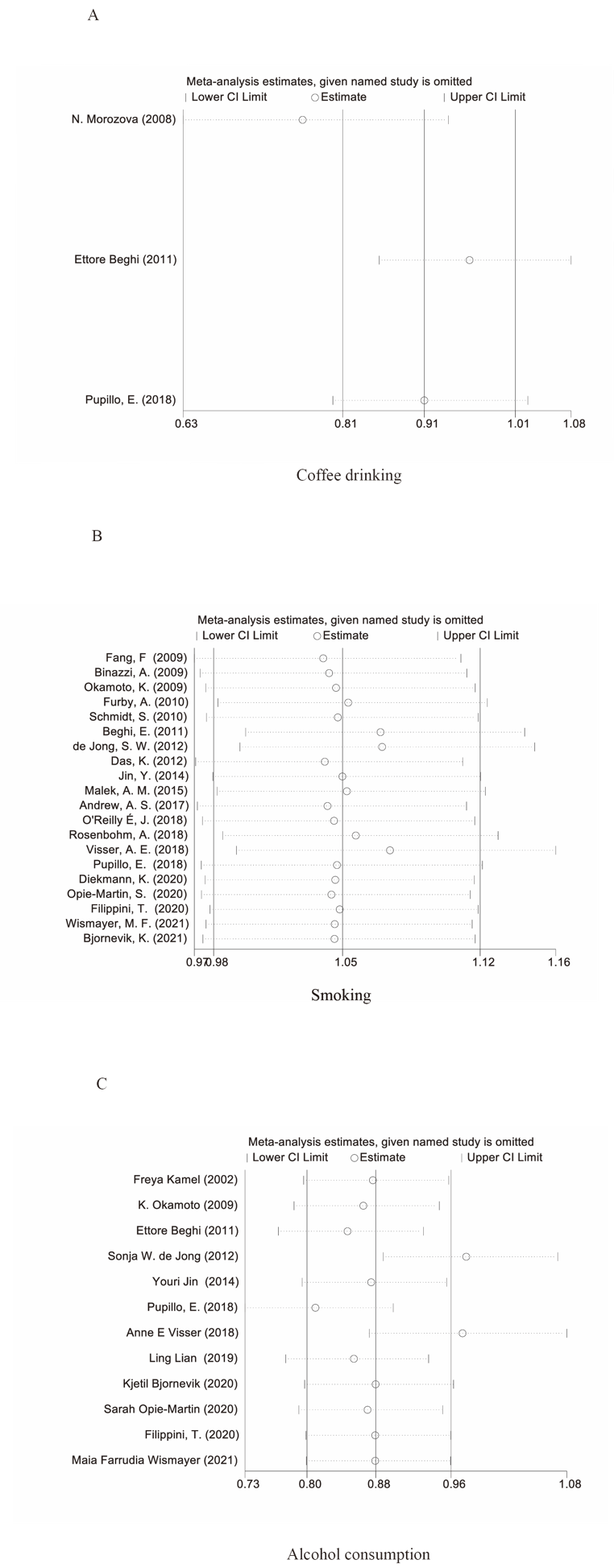


**Supp. Fig.17 Sensitivity analysis of other non-genetic factors**


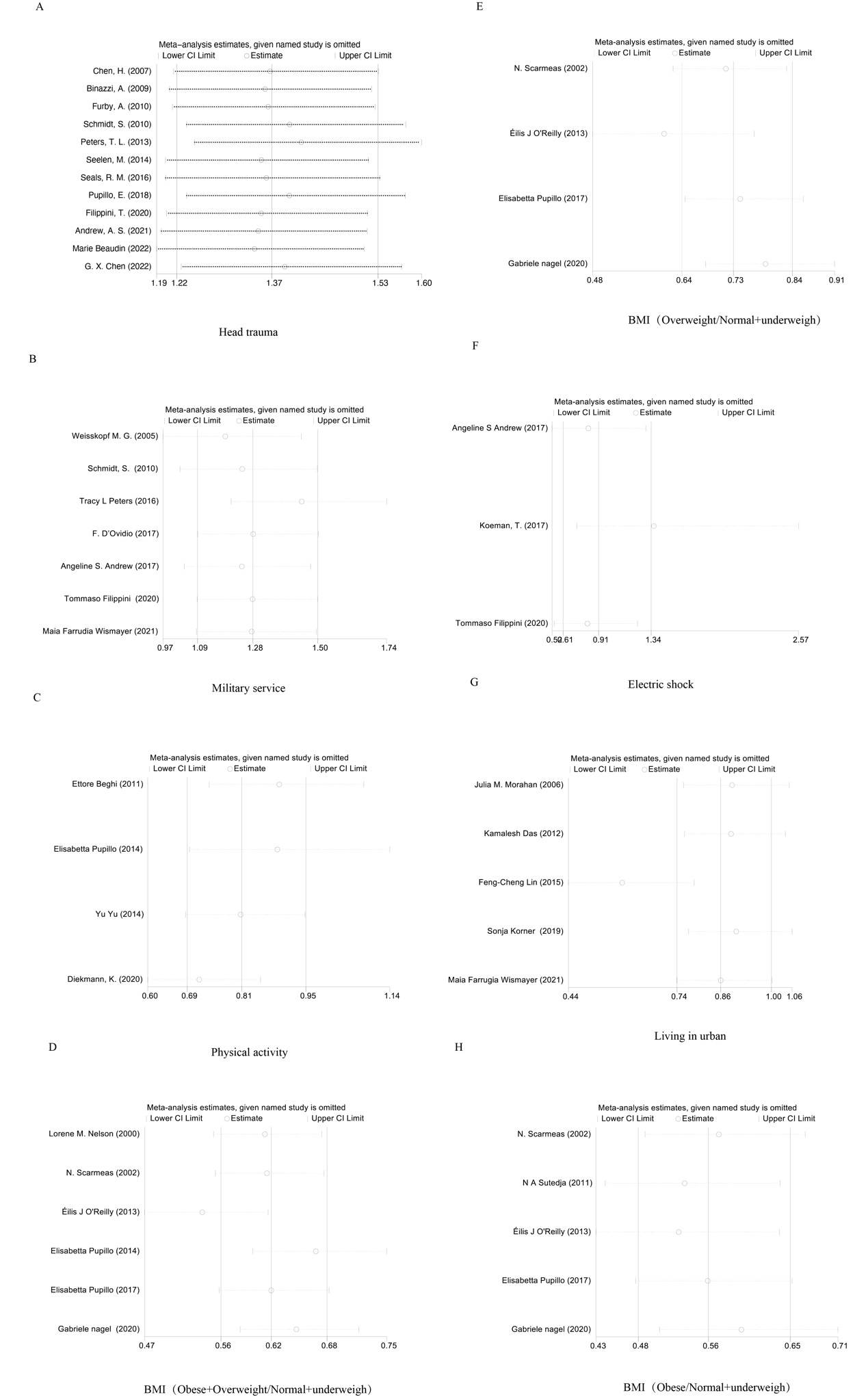


**Supp. Fig.18 Sensitivity analysis of causative/risk genes**


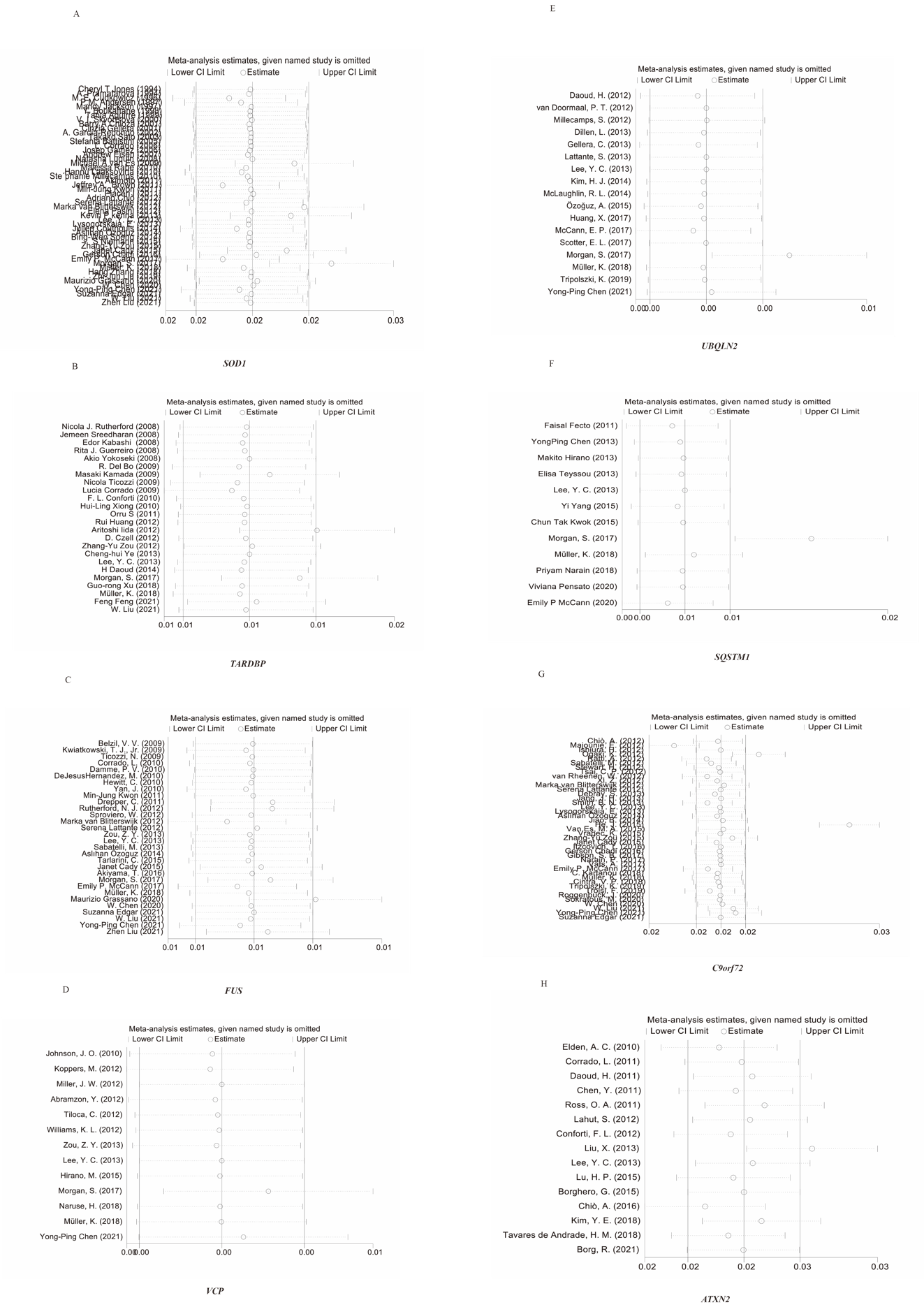

Supplement: Supplementary file 2 [file Data_Sheet_2.docx]
